# Development of SSR Markers and Genetic Diversity in White Birch (*Betula platyphylla*)

**DOI:** 10.1371/journal.pone.0125235

**Published:** 2015-04-29

**Authors:** Wei Hao, Shengji Wang, Huajing Liu, Boru Zhou, Xinwang Wang, Tingbo Jiang

**Affiliations:** 1 State Key Laboratory of Tree Genetics and Breeding, Northeast Forestry University, Harbin, China; 2 College of Resources and Environmental Sciences, Northeast Agricultural University, Harbin, China; 3 Texas A&M AgriLife Research and Extension Center, Texas A&M System, Dallas, Texas, United States of America; Louisiana State University Agricultural Center, UNITED STATES

## Abstract

In order to study genetic diversity of white birch (Betula platyphylla), 544 primer pairs were designed based on the genome-wide Solexa sequences. Among them, 215 primer pairs showed polymorphism between five genotypes and 111 primer pairs that presented clear visible bands in genotyping 41 white birch plants that were collected from 6 different geographical regions. A total of 717 alleles were obtained at 111 loci with a range of 2 to 12 alleles per locus. The results of statistic analysis showed that polymorphic frequency of the alleles ranged from 17% to 100% with a mean of 55.85%; polymorphism information content (PIC) of the loci was from 0.09 to 0.58 with a mean of 0.30; and gene diversity between the tested genotypes was from 0.01 to 0.66 with a mean of 0.36. The results also indicated that major allele frequency ranged from 0.39 to 1.00 with an mean of 0.75; expected heterozygosity from 0.22 to 0.54 with a mean of 0.46; observed heterozygosity from 0.02 to 0.95 with a mean of 0.26; Nei's index from 0.21 to 0.54 with a mean of 0.46; and Shannon's Information from 0.26 to 0.87 with a mean of 0.66. The 41 white birch genotypes at the 111 selected SSR loci showed low to moderate similarity (0.025-0.610), indicating complicated genetic diversity among the white birch collections. The UPGMA-based clustering analysis of the allelic constitution of 41 white birch genotypes at 111 SSR loci suggested that the six different geographical regions can be further separated into four clusters at a similarity coefficient of 0.22. Genotypes from Huanren and Liangshui provenances were grouped into Cluster I, genotypes from Xiaobeihu and Qingyuan provenances into Cluster II, genotypes from Finland provenance into Cluster III, and genotypes from Maoershan into Cluster IV. The information provided in this study could help for genetic improvement and germplasm conservation, evaluation and utilization in white birch tree breeding program.

## Introduction

Simple sequence repeats (SSRs), or microsatellite DNA, are short tandem repeats (1–6 bp long) of DNA sequence motifs that are widely distributed in eukaryotic organisms genomes [1–2]. The number of SSR motifs among different species shows polymorphism because of differences in repeated unit numbers [3]. SSRs are PCR-based markers that require low DNA amounts in the amplification of genomic DNA. Since SSR markers are generally co-dominant, multi-allelic, reproducible, and highly polymorphic [4–6], they have been widely applied in genetic linkage mapping, germplasmic resource investigation, phylogenetic analysis, DNA fingerprinting, and other genetic studies [7–9]. It has been demonstrated that SSR markers are suitable for studying genetic diversity and relationships between plant species, populations, and individuals [10–11].

White birch (*Betula platyphylla* Suk.), a deciduous broadleaf tree species, is widely distributed in the northeast and northwest of China, in where it plays an important role in timber production [12]. Because of its fast growth and easy regeneration, white birch is a typical pioneer tree as the secondary forest in these regions. In addition, white birch trees have an indispensable ecological role in the colonization of forest lands after harvesting and protection of wild fire damages in north China. They are also valuable for timber industries because of the compact and spotless qualities of wood [13].

Like other trees, white birch breeding takes a long time to develop a new variety by using phenotype-based traditional breeding methods because of its long life cycle. Because DNA sequence polymorphisms are directly associated with genotypes, a marker-assisted selection (MAS) strategy has been proposed and could be used to directly select desired progenies with target genotype. This method has incomparable superior to traditional breeding methods that infer genotypes from the phenotypes.

As aforementioned, SSR markers are among the best biomarkers in plant breeding programs. Therefore, white birch breeders attempted to use different methods to explore SSR markers in white birch genome. For example, Wu et al. [14] obtained 13 SSR markers from the genomic DNA library of *B*. *platyphylla* by using a PCR method. Ogyu et al. [15] obtained 184 SSR-contained clones from the SSR-enriched DNA library of *B*. *maximowicziana* and tested 15 SSR primer pairs, of which 8 SSR markers were successfully amplified polymorphic fragments. Kulju et al. [16] screened 38 SSR-contained clones from 17,300 clones in the genomic DNA library of *B*. *pendula* and developed 23 polymorphic SSR markers. Truong et al. [17] obtained 17 SSR-contained clones from 8,000 clones in the genomic DNA library of *B*. *pubescens* and found 3 polymorphic SSR markers. Recently, the expressed sequence tag (EST) has been widely used to develop SSR markers. Wang et al. [18] found 260 SSR motif-contained EST sequences from 2,548 ESTs (10.2%) in *B*. *platyphylla* and designed 45 EST-SSR primers that amplified polymorphic fragments in white birch genome. Lu et al. [19] obtained 331 SSR-contained EST from 3,028 EST sequences of *B*. *platyphylla* and developed 28 EST-SSR primers that successfully amplified polymorphic fragments.

One of the SSR applications is the genetic linkage mapping. By using 19 SSRs and 145 AFLP markers, Pekkinen et al. [20] built the first genetic linkage map of *B*. *pendula* genome. Jiang et al. [21] constructed high density genetic linkage maps in *B*. *platyphylla* and *B*. *pendula* species using AFLP and RAPD markers. To date, the numbers of SSR markers used for linkage mapping in *B*. *platyphylla* are limited. The numbers of SSR markers can saturate a high density genetic map, which is the foundation of cloning important genes of interested agronomic traits in white birch breeding program. High-throughput sequencing technologies make it possible to develop a large number of SSR markers base on the whole genome sequence information. In order to accelerate the process of germplasm evaluation and cultivar/or breeding line identification in white birch breeding program[22], the present study was to develop SSR markers based on white birch genome Solexa sequences and used these markers to genotype 41 white birch plants that were collected from six geographical regions in north China and Finland.

## Materials and Methods

No specific permissions were required for these locations/activities in this paper. And we confirm that the field studies did not involve endangered or protected species.

### 1.1 Materials

Seeds of 41 white birch genotypes were collected from 6 different geographical regions in Heilongjiang and Liaoning provinces in China and Finland (S1 Table) and sown in the greenhouse at the Tree Breeding Base of Northeast Forestry University, Harbin, China. Of them, 36 genotypes were from Huanren (3), Qingyuan (7), Xiaobeihu (7), Maoershan (15), and Liangshui (4) in Heilongjiang and Liaoning provinces, China, and the other five genotypes were imported from Finland. Young leaves were collected from the trees in the growing season and stored at -80°C for DNA extraction.

### 1.2 DNA Extraction

Total genomic DNA was extracted using Universal Genomic DNA Extraction Kit (TaKaRa, Dalian, China) following the manufacture’s instruction. DNA concentration and quality were checked and quantified using a NanoDrop 2000c Spectrophotometer. The DNA was stored at -20°C for sequencing and PCR analysis.

### 1.3 Solexa Sequences and SSR Primer Design

Sequencing of white birch genome was implemented by BGI (Shenzhen Company Ltd., Shenzhen, China) using the Solexa next-generation sequencing technology (Illumina GA). The short sequence reads were cleaned and then assembled by using the SOAPdonova software. The genome of *B*. *platyphylla* was estimated approximately 440 million base pairs across 28 chromosomes. The clean, assembled sequences were used to search SSRs by using software SSRIT [23]. Repeats containing dimer, trimer, tetramer, pentamer, and hexamer motifs which are longer than 20bp in general were selected for SSR primer design using Primer Premier 5.0 [23] by following standard parameters: target amplicon length of 100–500 bp, annealing temperatures of 50°C—70°C, GC contents of 50%- 70%, and primer size of 18–24 bp. The SSR primer pairs were synthesized at Sangon Biotech (Shanghai, China).

### 1.4 PCR assay and Detection

In order to detect SSR polymorphism, a feasible PCR condition was optimized. The total reaction mixture of 20 μl included 50 ng DNA, 1.0 μl of 10 μmol forward primer, 1.0 μl of 10 μmol reverse primer, 0.5μl of 10 mmol dNTP, 2 μl of 10× buffer (100 μmol Tris–HCl, 500 mmol KCl, 0.8% Nonidet P40), 2 μl of 25 mmol MgCl_2_, and 0.2 μl of Taq polymerase (5 U/μl). PCR amplification was performed in an MJ Research PTC-200 thermocycler (MJ Research, MA, USA), starting with an initial denaturation step of 94°C for 4 min, followed by 35 cycles of denaturation at 94°C for 1 min, annealing at appropriate temperature (depending on SSR primers) for 1 min and extension at 72°C for 30 sec, with a final extension step at 72°C for 10 min. The PCR products were subject to electrophoresis on 6% polyacrylamide denaturing gels in 1x TBE buffer and visualized by silver staining.

The PCR products were eluted from the gel using MiniBEST Agarose Gel DNA Extraction Kit Ver.3.0 (TaKaRa, Dalian, China) and cloned into pMD19-T Vector. The recombinant plasmid were transferred into *E*. *coli* by using a hot shock method and sequenced by GENEWIZ (Suzhou, China) using M13F (-47) and M13R (-48) primers.

### 1.5 Statistical Analysis

The visible band of each genotype was recorded as binary data: 1 = present of band and 0 = absent of band. Statistical components, including major allele frequency, polymorphism information content (PIC), gene diversity, observed heterozygosity (Ho), expected heterozygosity (He), Nei's index (1973), and Shannon's Information index, were computed by using the POPGENE (version 1.32) program. In order to generate a dendrogram showing the relationships of genetically diversified samples, cluster analyses were performed using the unweighted pair group method average (UPGMA) method (NTSYS-pc2.11a software) [24]. The dendrogram was visualized with the TreeView 1.6.6 [25].

## Results and Discussion

Molecular markers are widely used in plant genetics, breeding, biological diversity analysis, and cultivar identification since they can directly manifest genetic differences at the DNA level. SSR motifs are polymorphic, abundant, and randomly distributed in eukaryotic genomes [1]. Compared to other biomarkers, such as RAPDs and AFLPs, SSR markers are stable, co-dominant, and low cost. Therefore, they have been widely used in genetic analysis and genomic linkage mapping.

**Fig 1:**
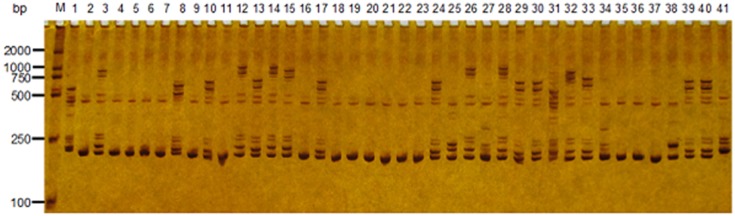
An example of SSR variation at the BP-293 locus across 41 white birch genotypes. M, DL2000 DNA ladder Marker; 1–41, each represents one of the 41 white birch plants.

High-throughput Solexa sequencing technology has provided an efficient tool to develop SSR markers. In the present study, 544 SSR primer pairs were designed from the white birch genomes (S2 Table) and tested polymorphism among five white birch genotypes. Of them, 215 showed polymorphisms with visible bands, indicating that 39.5% of SSR loci could be used for white birch genotyping. It also suggests that development of SSR markers from the high-throughput whole genome sequences is more efficient than from genomic DNA library and EST sequences, because the SSR markers from whole genome sequences are more wide distributed and then show higher rates of polymorphism. Of the 215 polymorphic loci, 111 solid loci were selected to genotype the 41 white birch genotypes (Table 1). As results, a total of 717 alleles were visualized across these 41 genotypes. The SSR allele numbers varied by loci ranged from 2 (Loci BP-016, BP-022, BP-080, BP-121, and BP-301) to 12 (BP-210). Fig 1 showed the PCR amplification profile of the locus BP-293 across the 41 white birch genotypes.

**Table 1:** Summary of polymorphism of 111 SSR markers selected from Solexa sequences of white birch genome.

Primer ID	Forward/Reverse primer sequences	Allele number	Annealing temperature / °C	Expected fragment size/ bp	Allele Frequency	Gene Diversity	PIC	Ho	He	Nei's (1973)	Shannon's Information index
BP-001	GAATGGAGATTGCTTCTCTCAGG	8	60	259	0.6341	0.464	0.3564	0.3659	0.4384	0.4331	0.6246
	GCCCCCAAATGTCCCAAATCCC		64								
BP-002	GGGTATGGGGATGAAATGGTTGG	3	62	192	0.9268	0.1356	0.1264	0.0732	0.5035	0.4973	0.6905
	CCAAATAAGCCCTAAGCCCAC		60								
BP-003	CGCGCTCTGATTGGACCACGCTC	7	67	235	0.8049	0.3141	0.2648	0.1707	0.4914	0.4854	0.6785
	CTGACCCTAACCCCAACCCTGAG		66								
BP-005	CAAAATGTCGGAGGCAGTGTCG	7	62	211	0.8537	0.2499	0.2186	0.1463	0.4953	0.4893	0.6824
	CCCGTTGCAAACCCTAAATCAC		60								
BP-008	GCACCTTTCGCAAGGAGAAACCGG	8	65	363	0.7805	0.3427	0.2839	0.2195	0.4818	0.4759	0.6689
	CTACTGTGGCCCATCAGCATTAGC		64								
BP-009	CAACGGCAATGACCTAGCGATACG	7	64	185	0.7317	0.3926	0.3155	0.2683	0.4697	0.464	0.6567
	CTTGTGTTACGAGGCCATAAGCC		62								
BP-010	GTCTGATAGTCATCGATCGAGCGAGGTTCGCTCTCACCTCCATCAAAGG	9	64	219	0.8049	0.3141	0.2648	0.1951	0.4869	0.481	0.674
			64								
BP-012	GGCTTACACCAAACCACGTTGCAG	5	64	229	0.7317	0.3926	0.3155	0.2683	0.4697	0.464	0.6567
	CTTTTCTCAGTCTCAGAGTGGGG		62								
BP-014	GACCGATTTAAACCCTCGCAGTG	8	62	294	0.5366	0.5378	0.4366	0.561	0.3975	0.3926	0.5816
	CAGCCATGTTTGCCTCATTCCATC		62								
BP-015	CGGTTGGTAGGGTAACCAAAG	8	60	198	0.8293	0.2832	0.2431	0.0976	0.5014	0.4952	0.6884
	CTGTCTCTCAAACCCCTGTTTC		60								
BP-016	GCTTCATTTCCTGGGACCTGATG	2	62	277	0.7317	0.4212	0.3743	0.9512	0.2867	0.2832	0.457
	CCTTCTTCAAGGATCACGGTAGACC		64								
BP-019	GCTTGGTTCGCTTGTTCGTCCATG	4	64	335	0.8537	0.2499	0.2186	0.1463	0.4953	0.4893	0.6824
	CATTCCGATCCGTTTCTCCCACC		64								
BP-022	GCTGGTGGACAACGATGGTTGCAG	2	65	219	0.5122	0.5889	0.5069	0.122	0.4986	0.4926	0.6857
	GTGAAGCGAGAGAACATGGCACC		64								
BP-028	GTTCTGAGTCTTGGGTAGTGGTG	3	62	190	0.7561	0.3795	0.3237	0.2439	0.5056	0.4994	0.766
	CCTTCAGTCCAACAAACCCTTC		60								
BP-029	GAGCCATGGATTCGTTGGTATCG	9	62	188	0.8293	0.2832	0.2431	0.1707	0.4914	0.4854	0.6785
	GCCTCACCATATCTTCACTCTCC		62								
BP-030	GATGAGGAGTAGAGAAAGCTCGG	9	62	189	0.561	0.4926	0.3713	0.3659	0.4384	0.4331	0.6246
	CGCGAAGGAGAGTTAACTGTGAG		62								
BP-034	GGGAAAGGGACAAGTATGAGCTTG	5	62	259	0.4878	0.6163	0.5401	0.1951	0.4869	0.481	0.674
	GAAAAAGAGAGGGTGGGGGGTTTC		64								
BP-041	GTATGAAGTGACTGGATGGGCAG	8	62	285	0.9268	0.1356	0.1264	0.0976	0.505	0.4988	0.692
	CCCATCTCCATCTCATTTGCAG		60								
BP-044	CCCTCACACGAAGCACCATTTAG	8	62	205	0.7317	0.3926	0.3155	0.3659	0.4553	0.4497	0.642
	CAGACACTCCGTCCATTCACAAC		62								
BP-050	CCCCAATCGAATGGAGAGAAAGAG	5	62	191	0.7805	0.3427	0.2839	0.2195	0.4818	0.4759	0.6689
	CTCTACACCCAACCAGTTCTTCCTC		64								
BP-053	GGCATGGCTCTTGTTTGTGCAG	9	62	251	0.9268	0.1356	0.1264	0.122	0.4986	0.4926	0.6857
	CAGGGATTCTGAAAAGTGGTCC		60								
BP-061	CGAGTCTCAGACAGACAGGAAGAG	8	64	206	0.7073	0.414	0.3283	0.2927	0.4628	0.4572	0.6497
	GTGAACTTGGGAAGTCACCCGTC		64								
BP-063	CACGTGCAGTGGATCGATAATC	8	60	134	0.9024	0.1761	0.1606	0.0732	0.5035	0.4973	0.6905
	GATCCACAGAGAGAATTCAGGC		60								
BP-065	GAGGATCCAATGCGGGAATGAAG	6	62	224	0.6829	0.4688	0.4076	0.3171	0.542	0.5354	0.8717
	CCCCAAGGACTGTCTTTGGTGAC		64								
BP-066	GGGTTTTTATGATGGGTTCGGG	3	60	213	0.8293	0.2832	0.2431	0.2439	0.4761	0.4703	0.6631
	GGAGTACATCTGGGTGCCCAATCC		65								
BP-067	GAGCCTGAGAGATGATTTGCAG	3	60	160	0.7317	0.3926	0.3155	0.1707	0.4914	0.4854	0.6785
	CTGGAAAAATCCAACCCCACCG		62								
BP-069	GGATTGAGGGAATGCGGGATTGAG	7	64	191	0.3902	0.6591	0.5849	0.2683	0.4697	0.464	0.6567
	CAATGGGGTGATAGTTTGAGAGGG		62								
BP-071	GCTCAACTCTGGCGGAACCGAACC	9	67	284	0.561	0.5318	0.4349	0.4146	0.4818	0.4759	0.6689
	CCCGTCTAAACTCGGCGATGTTCTC		65								
BP-072	GCCGAACATGAAACCGTACCTG	3	62	291	0.8537	0.2499	0.2186	1	0.2529	0.2499	0.4163
	CCATGTTTGGTTCCCGAGAAACC		62								
BP-073	GGCTTACTCGGGCGCCATGCTTGAG	3	68	178	0.8537	0.2499	0.2186	0.1463	0.4953	0.4893	0.6824
	GGTCCCTTAGGGCGTCTCCTCAGC		69								
BP-075	GCTTGAGTGCCACGAATTTGTCAC	5	62	221	0.9268	0.1356	0.1264	0.0488	0.505	0.4988	0.692
	GGGATGGTAGTTTGAGGGATCTG		62								
BP-076	GAAAGGGGAAAGGGAGTTGGGGATC	8	65	210	0.7073	0.414	0.3283	0.2927	0.4628	0.4572	0.6497
	GTCCCAAGCATTATTGGCGGTGGC		65								
BP-078	GAACCTCAATCCATCGCATACC	7	60	182	0.7073	0.414	0.3283	0.7073	0.2529	0.2499	0.4163
	GTCTTGAAGGCGAAACCACCTC		62								
BP-079	GTTGTTGAGCGTCTCGAACTTGAG	5	62	302	0.7561	0.3688	0.3008	0.3415	0.4472	0.4417	0.6337
	CGCGAAGTTTGACTAAGACCTCTC		62								
BP-080	CTGGTCAGAGGATCAGATGGTG	2	62	249	0.8293	0.2903	0.26	0.1707	0.5191	0.5128	0.7799
	CGGCCAGAGTTCATCTGATTTG		60								
BP-081	GAATCCCACAGTTTCTCCGGTTG	9	62	236	0.8537	0.2499	0.2186	0.1951	0.4869	0.481	0.674
	GCTGTTCTTGAATCTTGACCAGGC		62								
BP-085	CCCAAAGAAAAGACCTCCGCAGTG	5	64	261	0.8049	0.3141	0.2648	0.1951	0.4869	0.481	0.674
	GTTTGCTCGTGAGAGGAACATACC		62								
BP-089	GAAGTTGGCCATGGCCATGAAAG	8	62	167	0.8293	0.2903	0.26	0.1951	0.5014	0.4952	0.6884
	CTCCTTGTTCCTCCTCCTCATTG		62								
BP-097	CTCCCATGAGAATCTCTGCACTG	4	62	175	0.8537	0.2594	0.242	0.122	0.5059	0.4997	0.6928
	GCGTGTTATTGGGAGAAAAGGAGC		62								
BP-098	CACAGAATGCTCCTTTGATGCGAC	8	62	201	0.7317	0.3926	0.3155	0.1951	0.4869	0.481	0.674
	CGAGAGTTAGTGATGGAACGAAGC		62								
BP-102	CTCAGCAACCATACAGGAGGTAC	8	62	141	0.7561	0.3688	0.3008	0.2195	0.4914	0.4854	0.6785
	CAGAAGCCGAAAGAAAGCGTAG		60								
BP-110	GAGCGAGATTTGGTGGTCATACC	7	62	177	0.6098	0.4759	0.3627	0.3902	0.4291	0.4239	0.6149
	GTGGAGTAATGCCCACCTTATGC		62								
BP-111	GGCCAGGAGCAAGAAGAGAGAAAG	8	64	118	0.439	0.6496	0.5765	0.4146	0.4553	0.4497	0.642
	CTTCCCACTTCCCACATCCTCTTC		64								
BP-113	CACACTGCTGCCTGA	6	54	168	0.5122	0.4997	0.3749	0.5122	0.3734	0.3688	0.5555
	TCATAAAACCCTCAAAGAAT		50								
BP-115	TCTACGCTGTGACCAGTC	4	57	187	0.561	0.4926	0.3713	0.439	0.4086	0.4036	0.5934
	AGAATCCTAGCCTTTTCAAT		52								
BP-116	AATGCAGCATCTCTTACC	8	53	139	0.9024	0.1761	0.1606	0.122	0.4986	0.4926	0.6857
	CACGCAATAATATGGAAA		48								
BP-121	CCTTGTGTACTTGAGTAGTGC	2	54	152	0.4878	0.5449	0.4406	0.5122	0.4432	0.4378	0.7364
	TTGATCCCACCAGTTTATTGC		54								
BP-123	TCTCACCAAACCACTCACTCA	3	58	215	0.9512	0.0928	0.0885	0.0488	0.505	0.4988	0.692
	AAGAGCGTGGCAATGAACTC		58								
BP-124	CAGACGACAAAGCAAGCTGA	5	58	213	0.8049	0.3141	0.2648	0.1707	0.4914	0.4854	0.6785
	CATGCTCACATACAAGGCAAA		56								
BP-127	GAGAGAACCAAAACAGTAGACAGAGA	6	60	168	1	0.0092	0.0902	0.0488	0.505	0.4988	0.692
	GGCCTGTTCTTGATGACGAT		58								
BP-128	GGGGGTTGCTCTTCATTTTT	3	56	222	0.6829	0.4593	0.3895	0.9024	0.2168	0.2142	0.3708
	GGTTTCCTCGTCGGTTATGA		58								
BP-130	GTTAAGAAGGTGCGCCAGTC	5	60	254	0.7317	0.414	0.3597	0.2683	0.5276	0.5211	0.8287
	ACTAACCGCGCATAAACTGC		58								
BP-207	CAGCCTTCCTGCCTGCATGTGTG	9	66	167	0.8293	0.2832	0.2431	0.1707	0.4914	0.4854	0.6785
	CGAAGTCAGTTGTCAGCTTGTGG		62								
BP-208	GAGCTAGAGAGATGGGTGTGGCAG	7	65	199	0.6829	0.4331	0.3393	0.2927	0.4628	0.4572	0.6497
	CTCGTAACCAGTAACGTACCCACG		64								
BP-210	CCCTCTCCCCATGGTAATTGCATG	12	64	170	0.561	0.4926	0.3713	0.439	0.4086	0.4036	0.5934
	GGAGCCTCAAGGCAAGGTAGCTTC		65								
BP-212	CACGAGAGAGATCACGCTTTCCC	10	64	195	0.9024	0.1761	0.1606	0.0732	0.5035	0.4973	0.6905
	CCACCGCCAGAAACCCTTTGATC		64								
BP-213	CCATTGCTCTCTGAGATAAGGG	5	60	148	0.8537	0.2499	0.2186	0.1463	0.4953	0.4893	0.6824
	GCTCTAACGCTCTCTGACAGTTAC		62								
BP-214	CCAAAGCGAAGATGCTCACCGCTTG	7	65	282	0.6829	0.4331	0.3393	0.3171	0.4553	0.4497	0.642
	CTGTAGGGTTCAAGGGGCGAGAC		66								
BP-215	GCTACGATGGTGGTGGTTGGGTGG	9	67	191	0.8293	0.2832	0.2431	0.1707	0.4914	0.4854	0.6785
	CCTCTCTCTCTCTCTCCCTCTCTC		65								
BP-219	GAGGAGAAAAGGGGAATTTGCTGG	7	62	194	0.6098	0.5366	0.4674	0.3659	0.4818	0.4759	0.6689
	CTTCCTCCATGAATGAACGTCCC		62								
BP-226	GAGCTCCCAAGCATAACCGATCCTG	9	65	204	0.6585	0.4497	0.3486	0.3415	0.4472	0.4417	0.6337
	CCCTACACACCATACTCTCCCTCTC		65								
BP-229	GTTGAAGTTCGGGCAGACATAC	8	60	259	0.8293	0.2832	0.2431	0.1707	0.4914	0.4854	0.6785
	GACACAGCTGCCAAGCCTTATG		62								
BP-233	GAGAGAGAGGCGGCTCATGAATG	9	64	185	0.7317	0.3926	0.3155	0.2683	0.4697	0.464	0.6567
	GTGACCGGATGAGTTTTCAGTG		60								
BP-235	GAGAGAAAGTGTGGTGACCGTG	6	62	197	0.439	0.6425	0.5677	0.5366	0.3857	0.381	0.5689
	CCATAACTACTAACACCCACCG		60								
BP-236	GTCTTTCCCGGCGGAACACAGG	5	66	261	0.5854	0.4854	0.3676	0.5854	0.3327	0.3287	0.5104
	CTGTCGGCTCTGGGATACGACGC		67								
BP-237	GAGAGCTAACGCACAGTCGGAGAG	8	65	171	0.7317	0.4045	0.3401	0.0244	0.5059	0.4997	0.6928
	GGGTGGAAGGTGGGAAGAGGAATAC		65								
BP-244	GCCTAACAGTGTGGGTATGAAGC	5	62	184	0.9268	0.1356	0.1264	0.0732	0.5035	0.4973	0.6905
	GACAAAGCACTCCACCATAACC		60								
BP-245	CTTAAGTGCACAGTTGCACGCAC	8	62	208	0.9512	0.094	0.0918	0.0976	0.5062	0.5	0.6931
	CGATTCCTTTCTCTCTCCCTCC		62								
BP-248	GAACGCCATGCATATTTCGAGG	9	60	203	0.878	0.2142	0.1912	0.122	0.4986	0.4926	0.6857
	GTGGGGATGTAACATCTACGTG		60								
BP-249	GATAATAGGTTAGAGCTCGAGGGG	4	62	274	0.5854	0.4854	0.3676	0.4146	0.4553	0.4497	0.642
	GTGAACATGTTCTACTTCGGCGG		62								
BP-250	GCAGAGAAGGAGAATTGAGGTC	8	60	182	0.7561	0.3688	0.3008	0.2195	0.4914	0.4854	0.6785
	GTCCTTGAGAGTCATCGTGTTC		60								
BP-251	GTAAATGACACCACTGTGGGCATG	6	63	116	0.7073	0.414	0.3283	0.3415	0.4472	0.4417	0.6337
	CATTGGGTTGTCCTGAGTACCTC		62								
BP-253	GTCTTTCCCTTTAAGGCGGAGC	9	62	189	0.7073	0.414	0.3283	0.3659	0.4553	0.4497	0.642
	GAGCCTGAATCGCTAACGAACC		62								
BP-254	GTACTTCACAGGCCAAGAGAGAG	5	62	300	0.8537	0.2499	0.2186	0.1463	0.4953	0.4893	0.6824
	GATCGATGTTACTAGACAGGCCC		62								
BP-257	GGTGGAAATTGCAGGGGTTTTG	8	60	200	0.8293	0.2832	0.2431	0.1707	0.4986	0.4926	0.6857
	CGCATGCATGCATGCATTAGTG		60								
BP-258	GGTAGTAGCGTCAGTGTGAGAATG	7	62	246	0.9268	0.1356	0.1264	0.0488	0.5062	0.5	0.6931
	CTCATCTGCCTCCTTCACTGCTTC		64								
BP-259	GAGAGTGGGGACGTACATCAATAG	7	62	248	0.8049	0.3141	0.2648	0.2195	0.4914	0.4854	0.6785
	CAGACCCGAAATCCCGAAACTATC		62								
BP-268	GTCAAGCTCAAGAGATCCCTTG	8	60	304	0.7561	0.3688	0.3008	0.2683	0.4697	0.464	0.6567
	GTTTCTGTCGGCAAGGAAAAGG		60								
BP-269	GAGAGGTCTTTGGGTCAAGGAAG	7	62	244	0.6829	0.4331	0.3393	0.3659	0.4553	0.4497	0.642
	GTTCCTCGGCTATGAACCAAAGC		62								
BP-275	CAATGATGAAGCCCTAGCGACC	5	62	162	0.9756	0.0476	0.0465	0.0732	0.5059	0.4997	0.6928
	GTCAGGGGTGGGAGTTTACTTAC		62								
BP-276	CATTAATGGGTTTGGGCAGGCAC	5	62	211	0.7805	0.3427	0.2839	0.1463	0.5014	0.4952	0.6884
	CTAAGGAGGCATCTTATGGGTCC		62								
BP-279	GTCGGTGTAGGGCGACTGAGATATG	8	65	126	0.878	0.2189	0.2033	0.1463	0.4953	0.4893	0.6824
	CCTCTCCCCCATTTCGTCTGAAACC		65								
BP-282	CATTCCGCGTACTAAACGAGTTC	5	60	251	0.7073	0.414	0.3283	0.2439	0.4761	0.4703	0.6631
	CATACGGAATATGAGCAACGGCG		62								
BP-287	CAAACCCAACTAATCCTCGCG	7	60	275	0.878	0.2142	0.1912	0.122	0.4986	0.4926	0.6857
	CGCGTACTGGTTTGAATCCAGC		62								
BP-292	GCGTTTACACAGAGAGAGAGAG	6	60	219	0.9024	0.1761	0.1606	0.0976	0.5014	0.4952	0.6884
	CTTCTGTCTCTCACAGGTACACG		62								
BP-293	GCGAGAGGGAAAGTACACGAAAG	7	62	174	0.6829	0.4331	0.3393	0.3171	0.4553	0.4497	0.642
	GGTAGATCCCAAAGGTCTCTCTC		62								
BP-296	GAGAGAGATTGCAGGGGGGAGAAG	8	65	205	0.8049	0.3141	0.2648	0.1463	0.4953	0.4893	0.6824
	CCACTTCCCCCCATTTTCCCATCTC		65								
BP-298	CTATGGCGCACTCAAATCCTCATC	6	62	239	0.9024	0.1761	0.1606	0.0976	0.5014	0.4952	0.6884
	CACTTTGTGTGAAAGGCGCTTGG		62								
BP-299	CACCACCGAATGCCGTCGAAATCTC	8	65	154	0.8537	0.2499	0.2186	0.1463	0.4953	0.4893	0.6824
	GTGGCGTATTCCGGCGGTAGGTTTC		67								
BP-300	CAGCTCAAGGACACAGCAACCAG	5	64	367	0.6829	0.4331	0.3393	0.3171	0.4553	0.4497	0.642
	CAAGGGGGTGTTTCACAGCCGATC		65								
BP-301	GCGGGAACGGTTATCAGAATTCG	2	62	146	0.5366	0.5544	0.462	0.4634	0.495	0.489	0.8194
	GGATTTCGCCTTCTTTGAACCGC		62								
BP-304	GCTCTAACGAAACCCGCCGAAAG	5	64	188	0.5122	0.4997	0.3749	0.439	0.4086	0.4036	0.5934
	CTCCACCTCATCTTACCATTGGC		62								
BP-305	GCTGGCTTTGTGAACCCATGTG	6	62	227	0.7317	0.3926	0.3155	0.2927	0.4628	0.4572	0.6497
	GAGGTCTTGGCGCTCCAGAAAC		64								
BP-306	CATGCTCCAAGAACACACCTTG	5	60	248	0.7805	0.3427	0.2839	0.2195	0.4818	0.4759	0.6689
	CTGAACTAGACTCCGGGTTTCTC		62								
BP-309	GCTTGGCAGATGACACTTGAAG	9	60	185	0.7805	0.3427	0.2839	0.2195	0.4818	0.4759	0.6689
	GTGCAGACACTTGCATGGGAATG		62								
BP-311	GCGAAGAAGATAGCAAGAACCG	8	60	220	0.9268	0.1356	0.1264	0.0732	0.5035	0.4973	0.6905
	GAACCCCTGAAAGCTCTGTGTTG		62								
BP-313	GAAGGTTGAAACCTTCAGCACC	4	60	208	0.6829	0.4331	0.3393	0.2927	0.4761	0.4703	0.6631
	CCGGATATGGAAAGAACAGCAG		60								
BP-316	CAGGTTGGGAGAATAGATCGGAG	7	62	192	0.6585	0.4783	0.4015	0.6829	0.2701	0.2668	0.4372
	CTCTACATGCCACGTGTTCTCTC		62								
BP-319	GAAAGAAAGCACAGAGGAGCAC	7	60	195	0.439	0.6496	0.5765	0.4878	0.3857	0.381	0.5689
	GGCTGCCAACAAACAGTACTAC		60								
BP-320	GGTGTTGGGTCTCATGCAAATC	7	60	168	0.878	0.2142	0.1912	0.122	0.4986	0.4926	0.6857
	CCCTACCAGATCTTCAAATGGC		60								
BP-325	GAGGAGGATGTAAGCGAGGTAG	7	62	181	0.9512	0.0928	0.0885	0.0488	0.5062	0.5	0.6931
	CTGGACGATGAGAAGGACAACC		62								
BP-329	CTGAAGACCCTCCGATGCTTAAG	10	62	150	0.6098	0.4759	0.3627	0.5366	0.3604	0.356	0.5413
	CCATTTGACGAGGACTTCTGGAC		62								
BP-337	CCACAACGATGTAGGCATGAGAG	6	62	187	0.8293	0.2832	0.2431	0.2439	0.4761	0.4703	0.6631
	GTTTCCTTCCCATGCTGACTCTG		62								
BP-338	CACTTGTGCCCGATAACTCAAG	6	60	168	0.6585	0.4914	0.4259	0.2927	0.4869	0.481	0.674
	GCCACGATTTGGTCGTTCAAAC		60								
BP-344	GTGGATGCGGTTATTGGCCATATC	7	62	161	0.5122	0.4997	0.3749	0.4878	0.4086	0.4036	0.5934
	GATATGGCCAATAACCGCATCCAC		62								
BP-346	GAAAGCATGAGACCCGTCTT	6	58	161	0.6585	0.4497	0.3486	0.3415	0.4472	0.4417	0.6337
	AACCTAAACAGCCTGCCAAA		56								
BP-384	GCGACACACCCTACCATCTT	5	60	219	0.8537	0.2499	0.2186	0.1463	0.4953	0.4893	0.6824
	GGTGCACTTGCAGATGTGAT		58								
BP-389	TCGGATTGGTGGGTCTATTT	7	56	190	0.4146	0.646	0.5706	0.3171	0.4553	0.4497	0.642
	CGAAACCCCTTTGATGAGTT		56								
AF310847	CAGTGTTTGGACGGTGAGAA	6	58	209	0.6098	0.4759	0.3627	0.3902	0.4291	0.4239	0.6149
	CGGGTGAAGTAGACGGAACT		60								
AF310856	ACGCTTTCTTGATGTCAGCC	9	58	189	0.8293	0.2832	0.2431	0.1707	0.4914	0.4854	0.6785
	TCACCAAGTTCCTGGTGGAT		58								
AF310866	GGCCAACAGATATAAAACGACG	6	58	301	0.6341	0.464	0.3564	0.3659	0.4384	0.4331	0.6246
	TTTTAAATGCCCACCTTCCC		56								

The information of polymorphism of 111 SSR markers selected from Solexa sequences of white birch genome included primer ID, forward/reverse primer sequences, allele number, annealing temperature, expected fragment size, allele frequency, gene diversity, PIC, Ho,He, Nei's (1973), Shannon's information index.

**Note:** PIC, Polymorphism Information Content; H_O_, observed heterozygosity; H_E_, expected heterozygosity.

The polymorphic rates of 111 primer pairs across the 41 white birch genotypes ranged from 17% to 100% with an average of 55.85%. Eleven loci including BP-016, BP-019, BP-022, BP-028, BP-044, BP-065, BP-080, BP-097, BP-224, BP-250 and BP-301 presented 100% polymorphism, but AF310866 showed the lowest rate of polymorphism (17%). In this study, 111 selected polymorphic SSR loci amplified an average of 6.46 alleles per locus, which was higher than that reported by Wu et al.[14] (4.69 alleles per locus) and close to Kulju et al.[16]. The polymorphism information content (PIC) is determined by both allele numbers and allele frequency distribution and can be used to evaluate the variation of SSR alleles [26]. The results in this study showed that the 111 loci had low to moderate PIC, ranged from 0.09 (BP-127) to 0.58 (BP-069) with a mean of 0.30 (Table 1). Similarly, these SSR loci showed low to moderate gene diversity in a range of 0.01 (BP-127) to 0.66 (BP-069) with a mean of 0.36. The low to moderate PIC (0.30) and gene diversity (0.36) indicated that white birch genotypes from the six geographical locations had a lower genetic variation. Among the 111 SSR loci, locus BP-069 had the highest PIC (0.58) and gene diversity (0.66), which suggested that this marker can be used to differentiate most white birch genotypes in *Betula* breeding programs. In contrast, locus BP-127 had the lowest PIC (0.09) and gene diversity (0.01), indicating lower polymorphism and less utilization in the *Betula* cultivar identification. In addition, some other statistical analyses in the present study also reflected similar observations, higher major allele frequencies in a range from 0.39 to 1.00 with an average of 0.75, expected heterozygosity (He) from 0.22 to 0.54 with a mean of 0.46, observed heterozygosity (Ho) from 0.02 to 0.95 with a mean of 0.26. In addition, statistical analysis showed that Nei's index from 0.21 to 0.54 with a mean of 0.46 and Shannon's Information index from 0.26 to 0.87 with a mean of 0.66, indicating a moderate genetic distance among the 41 white birch genotypes. These results also were reflected by the genetic diversity analysis below.

**Fig 2:**
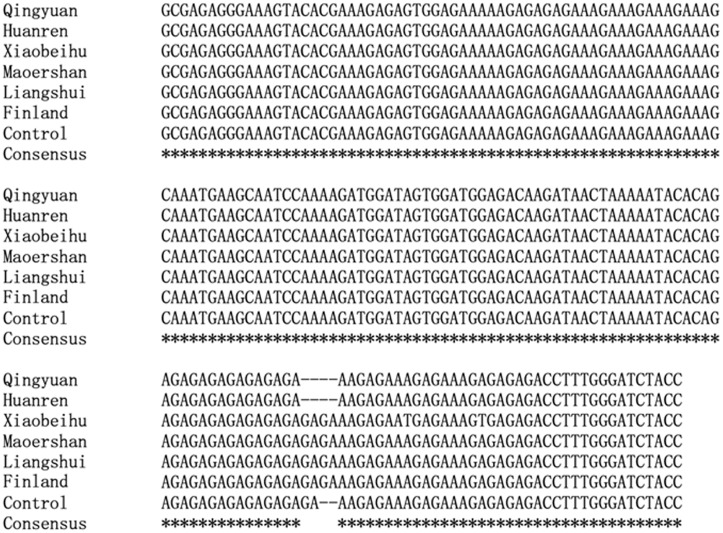
Multiple sequence alignment of BP-293 showing the position of SSR motifs, and expansion and contraction of the motif.

To verify the genetic basis of sequence length variation, The PCR products were re-sequenced. The alignment profile of multiple sequences of BP-293 locus was illustrated in Fig 2. Sequence lengths ranged from 172 bp to 176 bp and the numbers of the SSR motifs ranged from 8 to 10. The results indicate that the PCR truly amplified the targets containing the expected SSR motif.

**Fig 3:**
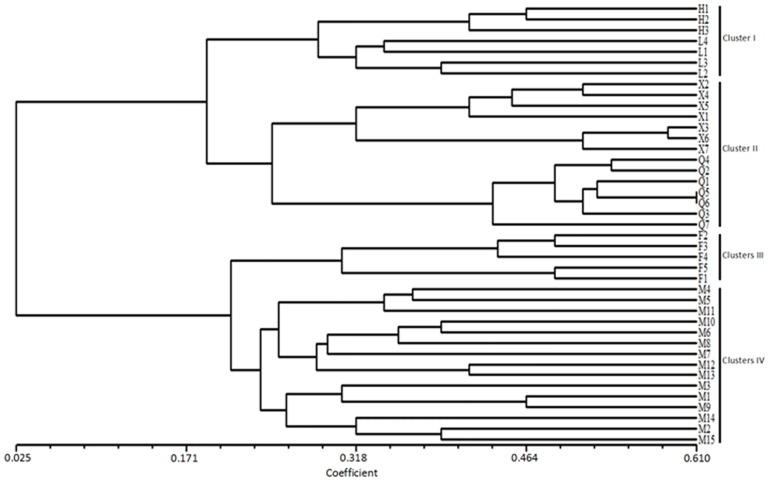
The unweighted pair group method average (UPGMA) based dendrogram of 41 white birch genotypes from six geographical locations, based on their allelic constitution at 111 SSR loci. H1-H3, Huanren provenance, China; L1-L4, Liangshui provenance, China; X1-X7, Xiaobeihu provenance, China; Q1-Q7, Qingyuan provenance, China; M1-M15, Maoershan provenance, China; F1-F5, Finland provenance, Finland.

Genetic diversity is a result of gene evolution in plant species [27] and becomes a foundation of the genetic improvement of species. Analyses of genetic diversity by using molecular markers could provide better understanding of genetic background of white birch cultivars. The results of the present study indicated that the white birch trees from six geographical locations had low to moderate similarity (0.025–0.610) and could be further separated into four clusters at a similarity coefficient of 0.22 (Fig 3). Genotypes from Huanren and Liangshui were closely related and grouped into the cluster I, and the genotypes from Xiaobeihu and Qingyuan into cluster II. Genotypes from Finland, and Maoershan were apparently different from each other and from the other groups as well, and grouped into the clusters III and IV respectively. The clusters of genotypes were apparently agreed with their provenances, suggested that the SSR primers used in this study can effectively distinguish white birch germplasm. The genetic relationships between these genotypes might provide useful information for genetic improvement and germplasm conservation, evaluation and utilization in white birch tree breeding program.

## Supporting Information

S1 Table The tested white birch materials for SSR analysis. The information of tested white birch materials for SSR analysis.
(DOCX)

S2 Table Information of 544 SSR primer pairs. The details of 544 SSR primer pairs included probe accessions, primer ID, repeat motif, forward/reverse primer sequence, annealing temperature (Tm), expected product size, observed fragment sizes in genotypes 1–5, number of alleles obtained.
(DOCX)
