# Correction: Development of SSR Markers and Genetic Diversity in White Birch (*Betula platyphylla*)

**DOI:** 10.1371/journal.pone.0129758

**Published:** 2015-06-01

**Authors:** Wei Hao, Shengji Wang, Huajing Liu, Boru Zhou, Xinwang Wang, Tingbo Jiang

An affiliation is missing for the fifth author. Xinwang Wang is affiliated with #1, #3, and with #4 USDA-ARS, Southern Horticultural Laboratory, Poplarville, Mississippi, United States of America.

## References

[1] Hao W, Wang S, Liu H, Zhou B, Wang X, Jiang T (2015) Development of SSR Markers and Genetic Diversity in White Birch (*Betula platyphylla*). PLoS ONE 10(4): e0125235 doi: 10.1371/journal.pone.0125235 25923698 10.1371/journal.pone.0125235 PMC4414481

