# Supplementary material for: Development of SSR Markers and Genetic Diversity in White Birch (Betula platyphylla)
Source: PLoS One. 2015 Apr 29;10(4):e0125235. doi: 10.1371/journal.pone.0125235 (PMC4414481; doi:10.1371/journal.pone.0125235)
Supplement: S1 Table — The information of tested white birch materials for SSR analysis. (DOCX) [file pone.0125235.s001.docx]

S1 Table. The tested white birch materials for SSR analysis. The information of tested white birch materials for SSR analysis.

S1 Table _Dataset.doc

| **Source** | **Number** | **Type** | **Origin** |
| --- | --- | --- | --- |
| Huanren provenances | 3 | *Betula platyphylla* | Huanren Laotudingzi National Nature Reserve. Liaoning provinces. China |
| Qingyuan provenances | 7 | *Betula platyphylla* | Qingyuan Forest Ecology Experimental Station, Institute of Applied Ecology, Chinese Academy of Sciences. Liaoning provinces. China |
| Xianbeihu provenances | 7 | *Betula platyphylla* | Xiaobeihu National Nature Reserve. Heilongjiang provinces. China |
| Maoershan provenances | 15 | *Betula platyphylla* | Maoershan National Forest Park. Heilongjiang provinces. China |
| Liangshui provenances | 4 | *Betula platyphylla* | Liangshui National Nature Reserve. Heilongjiang provinces. China |
| Finland provenances | 5 | *Betula platyphylla* | Finnish Forest Research Institute (Metla). Finland |

**Huanren Laotudingzi National Nature Reserve** is located 41° 16 '38 "— 41° 21' 10 " north latitude and 124° 49 '06 "— 124 °57 '08 " east longitude.

**Qingyuan Forest Ecology Experimental Station, Institute of Applied Ecology, Chinese Academy of Sciences** is located 41°51′102 ″north latitude and 124°54′543 ″ east longitude.

**Xianbeihu National Nature Reserve** is located 44°03´16"~44°18´59" north latitude and 128°33´07"~128°45´48" east longitude**.**

**Maoershan National Forest Park** is located 45°20'~45°25' north latitude and 127°30'~127°34' east longitude.

**Liangshui National Nature Reserve** is located 47°10′50″ north latitude and 128°53′20″ east longitude.
