# Supplementary material for: Development of SSR Markers and Genetic Diversity in White Birch (Betula platyphylla)
Source: PLoS One. 2015 Apr 29;10(4):e0125235. doi: 10.1371/journal.pone.0125235 (PMC4414481; doi:10.1371/journal.pone.0125235)
Supplement: S2 Table — The details of 544 SSR primer pairs included probe accessions, primer ID, repeat motif, forward/reverse primer sequence, annealing temperature (Tm), expected product size, observed fragment sizes in genotypes 1–5, number of alleles obtained. (DOCX) [file pone.0125235.s002.docx]

**S2 Table. Information of 544 SSR primer pairs. The details of 544 SSR primer pairs included probe accessions, primer ID, repeat motif, forward/reverse primer sequence, annealing temperature (Tm), expected product size, observed fragment sizes in genotypes 1 - 5, number of alleles obtained.**

**S2 Table _Dataset.doc**

| **probe accessions** | **primer ID** | **SSR motif** | **forward/reverse primer sequence** | **Tm (°C)** | **expected product size (bp)** | **size obtained in genotype 1** | **size obtained in genotype 2** | **size obtained in genotype 3** | **size obtained in genotype 4** | **size obtained in genotype 5** | **number of alleles obtained** |
| --- | --- | --- | --- | --- | --- | --- | --- | --- | --- | --- | --- |
| Pr032284614 | BP-001 | (AG) _10_ | GAATGGAGATTGCTTCTCTCAGG | 60 | 259 | 250 | 250 | 260 | 260 | 260 | 6--8 |
|  |  |  | GCCCCCAAATGTCCCAAATCCC | 64 |  |  |  |  |  |  |  |
| Pr032284615 | BP-002 | (AG) _10_ | GGGTATGGGGATGAAATGGTTGG | 62 | 192 | 190 | 190 | 205 | 190 | 190 | 2--3 |
|  |  |  | CCAAATAAGCCCTAAGCCCAC | 60 |  |  |  |  |  |  |  |
| Pr032284616 | BP-003 | (AG) _10_ | CGCGCTCTGATTGGACCACGCTC | 67 | 235 | 230 | 230 | 230 | 235 | 235 | 4--7 |
|  |  |  | CTGACCCTAACCCCAACCCTGAG | 66 |  |  |  |  |  |  |  |
| Pr032284617 | BP-004 | (AG) _10_ | GGCCTCCAGTCATGAGACTGAAGGC | 67 | 198 | 200 | 200 | 200 | 200 | 200 | 5--5 |
|  |  |  | CACAAGAGCCCAAGAGGGACCATG | 65 |  |  |  |  |  |  |  |
| Pr032284618 | BP-005 | (AG) _10_ | CAAAATGTCGGAGGCAGTGTCG | 62 | 211 | 215 | 210 | 210 | 210 | 215 | 4--7 |
|  |  |  | CCCGTTGCAAACCCTAAATCAC | 60 |  |  |  |  |  |  |  |
| Pr032284619 | BP-006 | (AG) _10_ | CGGTCACCCCTTCCAATATTTCCTG | 64 | 179 | 180 | 180 | 180 | 180 | 180 | 4--6 |
|  |  |  | CCCCAACCAGAATTAGCTAACCCG | 64 |  |  |  |  |  |  |  |
| Pr032284620 | BP-007 | (AG) _10_ | GTAACTGTTCTGGAAGGCCCAAAG | 62 | 260 | 0 | 0 | 0 | 0 | 0 |  |
|  |  |  | CTAGTGAGCAAAGAGTTCCCAC | 60 |  |  |  |  |  |  |  |
| Pr032284621 | BP-008 | (AG) _10_ | GCACCTTTCGCAAGGAGAAACCGG | 65 | 363 | 360 | 360 | 360 | 360 | 365 | 5--8 |
|  |  |  | CTACTGTGGCCCATCAGCATTAGC | 64 |  |  |  |  |  |  |  |
| Pr032284622 | BP-009 | (AG) _10_ | CAACGGCAATGACCTAGCGATACG | 64 | 185 | 185 | 185 | 185 | 195 | 185 | 4--7 |
|  |  |  | CTTGTGTTACGAGGCCATAAGCC | 62 |  |  |  |  |  |  |  |
| Pr032284623 | BP-010 | (AG) _10_ | GTCTGATAGTCATCGATCGAGCGAG | 64 | 219 | 220 | 225 | 225 | 225 | 225 | 6--9 |
|  |  |  | GTTCGCTCTCACCTCCATCAAAGG | 64 |  |  |  |  |  |  |  |
| Pr032284624 | BP-011 | (AG) _10_ | GGCCATCGTGTGTAGGGTAAAG | 62 | 225 | 225 | 225 | 225 | 225 | 225 | 3--3 |
|  |  |  | CAGATGTGAGCTAACGCGTTCCC | 64 |  |  |  |  |  |  |  |
| Pr032284625 | BP-012 | (AG) _10_ | GGCTTACACCAAACCACGTTGCAG | 64 | 229 | 229 | 229 | 240 | 229 | 240 | 2--5 |
|  |  |  | CTTTTCTCAGTCTCAGAGTGGGG | 62 |  |  |  |  |  |  |  |
| Pr032284626 | BP-013 | (AG) _10_ | CCGCTGAACTCCTAGTATTGACC | 62 | 255 | 250 | 250 | 250 | 250 | 250 | 3--3 |
|  |  |  | CATGCGGCTTTACTAGAAGAGGTG | 62 |  |  |  |  |  |  |  |
| Pr032284627 | BP-014 | (AG) _10_ | GACCGATTTAAACCCTCGCAGTG | 62 | 294 | 294 | 294 | 285 | 294 | 285 | 5--8 |
|  |  |  | CAGCCATGTTTGCCTCATTCCATC | 62 |  |  |  |  |  |  |  |
| Pr032284628 | BP-015 | (AG) _10_ | CGGTTGGTAGGGTAACCAAAG | 60 | 198 | 200 | 200 | 210 | 200 | 200 | 5--8 |
|  |  |  | CTGTCTCTCAAACCCCTGTTTC | 60 |  |  |  |  |  |  |  |
| Pr032284629 | BP-016 | (AG) _10_ | GCTTCATTTCCTGGGACCTGATG | 62 | 277 | 275 | 275 | 275 | 200 | 200 | 2--2 |
|  |  |  | CCTTCTTCAAGGATCACGGTAGACC | 64 |  |  |  |  |  |  |  |
| Pr032284630 | BP-017 | (AG) _10_ | GGCTCGACTGAGTTTGTTACGGC | 64 | 217 | 0 | 0 | 0 | 0 | 0 |  |
|  |  |  | CCCTGCTAACCCTAATGCCTTC | 64 |  |  |  |  |  |  |  |
| Pr032284631 | BP-018 | (AG) _10_ | GCTTGTGTTCAAATGGGTGTGG | 60 | 195 | 200 | 200 | 200 | 200 | 200 | 5--5 |
|  |  |  | GAAGGCTACAGGCGTTTTGGTAG | 62 |  |  |  |  |  |  |  |
| Pr032284632 | BP-019 | (AG) _10_ | GCTTGGTTCGCTTGTTCGTCCATG | 64 | 335 | 335 | 335 | 350 | 335 | 335 | 4--4 |
|  |  |  | CATTCCGATCCGTTTCTCCCACC | 64 |  |  |  |  |  |  |  |
| Pr032284633 | BP-020 | (AG) _10_ | CGCTATGGCTGGTATTTCATGGCC | 64 | 330 | 0 | 0 | 0 | 0 | 0 |  |
|  |  |  | GTGAATCCCACGCTCATCCACAAG | 64 |  |  |  |  |  |  |  |
| Pr032284634 | BP-021 | (AG) _10_ | CCACTCCTCCACCACTCTCCTTG | 66 | 276 | 0 | 0 | 0 | 0 | 0 |  |
|  |  |  | GGGGTGGAAATCCTGTCCCAAGC | 66 |  |  |  |  |  |  |  |
| Pr032284635 | BP-022 | (AG) _10_ | GCTGGTGGACAACGATGGTTGCAG | 65 | 219 | 220 | 225 | 225 | 220 | 220 | 2--2 |
|  |  |  | GTGAAGCGAGAGAACATGGCACC | 64 |  |  |  |  |  |  |  |
| Pr032284636 | BP-023 | (AG) _10_ | GCTTCTTGCTCCGAGATTTCGCTG | 64 | 116 | 116 | 116 | 116 | 125 | 116 | 4--8 |
|  |  |  | GACCAACGACGTCAACGGCAACAAC | 65 |  |  |  |  |  |  |  |
| Pr032284637 | BP-024 | (AG) _10_ | GGCAATTGGCACTACTTCTGTTGG | 62 | 288 | 0 | 0 | 0 | 0 | 0 |  |
|  |  |  | GACCCCTCCAAGACAACAAAATGC | 62 |  |  |  |  |  |  |  |
| Pr032284638 | BP-025 | (AG) _10_ | CAGAAACAATCACACCGCAGCAAC | 62 | 225 | 225 | 225 | 225 | 225 | 225 | 4--4 |
|  |  |  | CATTCCGTCCCGTAAATTAAGCGC | 62 |  |  |  |  |  |  |  |
| Pr032284639 | BP-026 | (AG) _10_ | GCTCAGTAACTGCTTACAAGCG | 60 | 348 | 0 | 0 | 0 | 0 | 0 |  |
|  |  |  | GATAGCTCACTGTCGTCAGCTTC | 62 |  |  |  |  |  |  |  |
| Pr032284640 | BP-027 | (AG) _10_ | GTGAACAGCCCTCTTGCTCATTCC | 64 | 221 | 225 | 225 | 225 | 225 | 225 | 2--2 |
|  |  |  | GGTTAAGCGTTCGTACACACCACC | 64 |  |  |  |  |  |  |  |
| Pr032284641 | BP-028 | (AG) _10_ | GTTCTGAGTCTTGGGTAGTGGTG | 62 | 190 | 195 | 190 | 190 | 190 | 190 | 3--3 |
|  |  |  | CCTTCAGTCCAACAAACCCTTC | 60 |  |  |  |  |  |  |  |
| Pr032284642 | BP-029 | (AG) _10_ | GAGCCATGGATTCGTTGGTATCG | 62 | 188 | 195 | 195 | 188 | 195 | 195 | 7--9 |
|  |  |  | GCCTCACCATATCTTCACTCTCC | 62 |  |  |  |  |  |  |  |
| Pr032284643 | BP-030 | (AG) _10_ | GATGAGGAGTAGAGAAAGCTCGG | 62 | 189 | 190 | 200 | 200 | 200 | 200 | 6--9 |
|  |  |  | CGCGAAGGAGAGTTAACTGTGAG | 62 |  |  |  |  |  |  |  |
| Pr032284644 | BP-031 | (AG) _10_ | GATATTGCTGGGAACGATCGACG | 62 | 160 | 150 | 150 | 150 | 150 | 150 | 5--11 |
|  |  |  | GTTAACAACCACCAATCCCAACCC | 62 |  |  |  |  |  |  |  |
| Pr032284645 | BP-032 | (AG) _10_ | GCGTGAAAAGGGCATGTATGCAAGG | 64 | 230 | 0 | 0 | 0 | 0 | 0 |  |
|  |  |  | CTGCGGGATTACGTGAATAGGTTCC | 64 |  |  |  |  |  |  |  |
| Pr032284646 | BP-033 | (AG) _10_ | CACTGTGCTTATCAGATGCCTCGTG | 64 | 210 | 0 | 0 | 0 | 0 | 0 |  |
|  |  |  | GGCAAAAACAGAGAAGGCTCACCTG | 64 |  |  |  |  |  |  |  |
| Pr032284647 | BP-034 | (AG) _10_ | GGGAAAGGGACAAGTATGAGCTTG | 62 | 259 | 259 | 265 | 259 | 259 | 265 | 3--5 |
|  |  |  | GAAAAAGAGAGGGTGGGGGGTTTC | 64 |  |  |  |  |  |  |  |
| Pr032284648 | BP-035 | (AG) _10_ | GTTGGAAGCCATCAGAGAGAGAG | 62 | 207 | 0 | 0 | 0 | 0 | 0 |  |
|  |  |  | GAGCGCCCAAAAGGGCCAAAAAC | 64 |  |  |  |  |  |  |  |
| Pr032284649 | BP-036 | (AG) _10_ | CTACAGCCAAACAAACCCTGGG | 62 | 239 | 0 | 0 | 0 | 0 | 0 |  |
|  |  |  | GATCGACGACGCCACCAACAAG | 64 |  |  |  |  |  |  |  |
| Pr032284650 | BP-037 | (AG) _10_ | CGGGAAGCACGGATCAGAAAAAAG | 62 | 195 | 200 | 200 | 200 | 200 | 200 | 2--7 |
|  |  |  | CTTCCCTTCCATTAATGCCCCCTCC | 65 |  |  |  |  |  |  |  |
| Pr032284651 | BP-038 | (AG) _10_ | GAAGCGGATAAACATGAACCCCCG | 64 | 190 | 0 | 0 | 0 | 0 | 0 |  |
|  |  |  | CTGAGTCTCCTCGGACAAACAGAC | 64 |  |  |  |  |  |  |  |
| Pr032284652 | BP-039 | (AG) _10_ | GGCAGGTATGCCCCACATTACAAG | 64 | 257 | 250 | 250 | 250 | 250 | 250 | 3--3 |
|  |  |  | CGCCTACTTTGTGCCCCACAATTG | 64 |  |  |  |  |  |  |  |
| Pr032284653 | BP-040 | (AG) _10_ | GCCAACCTACAAACCAGAAGAACC | 62 | 307 | 0 | 0 | 0 | 0 | 0 |  |
|  |  |  | CTTCTTCTAGACCCTTCTCTGCTC | 62 |  |  |  |  |  |  |  |
| Pr032284654 | BP-041 | (AG) _10_ | GTATGAAGTGACTGGATGGGCAG | 62 | 285 | 285 | 285 | 285 | 285 | 285 | 5--8 |
|  |  |  | CCCATCTCCATCTCATTTGCAG | 60 |  |  |  |  |  |  |  |
| Pr032284655 | BP-042 | (AG) _10_ | GAAGAGCGGACTTACCGTTATG | 60 | 155 | 155 | 155 | 155 | 155 | 155 | 2--5 |
|  |  |  | CCCAATATTTCTTCATGCCCGC | 60 |  |  |  |  |  |  |  |
| Pr032284656 | BP-043 | (AG) _10_ | GGGCATGAGAGAGTTGTAGAGAG | 62 | 168 | 0 | 0 | 0 | 0 | 0 |  |
|  |  |  | CAGATGCAAGACACCCACCAAATC | 62 |  |  |  |  |  |  |  |
| Pr032284657 | BP-044 | (AG) _10_ | CCCTCACACGAAGCACCATTTAG | 62 | 205 | 205 | 205 | 205 | 205 | 205 | 8--8 |
|  |  |  | CAGACACTCCGTCCATTCACAAC | 62 |  |  |  |  |  |  |  |
| Pr032284658 | BP-045 | (AG) _10_ | GCACTTGCTTTCGACCTAAGCCCC | 65 | 173 | 173 | 173 | 173 | 173 | 173 | 3--6 |
|  |  |  | CTGGACATTACGGGCAGAAGAGG | 64 |  |  |  |  |  |  |  |
| Pr032284659 | BP-046 | (AG) _10_ | GCACTAGCACAAGGGAATGTGC | 62 | 145 | 150 | 150 | 150 | 150 | 150 | 4--4 |
|  |  |  | GAGCAAGCCCTAAGAGCAACAC | 62 |  |  |  |  |  |  |  |
| Pr032284660 | BP-047 | (AG) _10_ | GCAGAAGATGGAAATGCATGCAGG | 62 | 160 | 160 | 160 | 160 | 160 | 160 | 1--3 |
|  |  |  | CTACTAAAAGTGTCTTCAGGCCGC | 62 |  |  |  |  |  |  |  |
| Pr032284661 | BP-048 | (AG) _10_ | CCATTGCCTGGCAAATCTCAGTC | 62 | 272 | 275 | 275 | 275 | 275 | 275 | 2--2 |
|  |  |  | GCTTCGTGGGAAGATTTCCTTTGC | 62 |  |  |  |  |  |  |  |
| Pr032284662 | BP-049 | (AG) _10_ | GCTCAGACCCAATTCCAGAAGAAG | 62 | 171 | 0 | 0 | 0 | 0 | 0 |  |
|  |  |  | CTCTCTCCAAGCTTCGACTTTGAC | 62 |  |  |  |  |  |  |  |
| Pr032284663 | BP-050 | (AG) _10_ | CCCCAATCGAATGGAGAGAAAGAG | 62 | 191 | 200 | 200 | 200 | 191 | 200 | 3--5 |
|  |  |  | CTCTACACCCAACCAGTTCTTCCTC | 64 |  |  |  |  |  |  |  |
| Pr032284664 | BP-051 | (AG) _10_ | CCCCTCTACTCTGTACATCTGTAC | 62 | 172 | 0 | 0 | 0 | 0 | 0 |  |
|  |  |  | CTTCTCCTTCTTCCTCTCCATCTC | 62 |  |  |  |  |  |  |  |
| Pr032284665 | BP-052 | (AG) _10_ | CCACACAAGGCTCCATATTGCTAC | 62 | 230 | 230 | 230 | 230 | 230 | 230 | 4--4 |
|  |  |  | CTATAAGGGCCCTCTCACAATTGC | 62 |  |  |  |  |  |  |  |
| Pr032284666 | BP-053 | (AG) _10_ | GGCATGGCTCTTGTTTGTGCAG | 62 | 251 | 250 | 250 | 250 | 250 | 250 | 5--9 |
|  |  |  | CAGGGATTCTGAAAAGTGGTCC | 60 |  |  |  |  |  |  |  |
| Pr032284667 | BP-054 | (AG) _10_ | GCAGAGCCGAAGACAACTATTG | 60 | 113 | 0 | 0 | 0 | 0 | 0 |  |
|  |  |  | CACAAAATACCACAAGCCAGCC | 60 |  |  |  |  |  |  |  |
| Pr032284668 | BP-055 | (AG) _10_ | CCACACATAAACTATCCCCACCTC | 62 | 150 | 125 | 125 | 125 | 125 | 125 | 4--6 |
|  |  |  | CTTCAGAAACCCCCCTTTCTCTC | 62 |  |  |  |  |  |  |  |
| Pr032284669 | BP-056 | (AG) _10_ | GAGAGAGATGGGTGTTTACTGCTG | 62 | 269 | 0 | 0 | 0 | 0 | 0 |  |
|  |  |  | CACGTTGGTTCCTAGTTCCTACTC | 62 |  |  |  |  |  |  |  |
| Pr032284670 | BP-057 | (AG) _10_ | GAACTGTATAGGAAATGCGTGGGC | 62 | 161 | 0 | 0 | 0 | 0 | 0 |  |
|  |  |  | GAAAAGCGTTTCTACCACTGCCAG | 62 |  |  |  |  |  |  |  |
| Pr032284671 | BP-058 | (AG) _10_ | GTGACGATAACGGTCGGTAATCG | 64 | 121 | 120 | 120 | 120 | 120 | 120 | 2--5 |
|  |  |  | CTCTAGCTAGCCCAGCTTGTATG | 62 |  |  |  |  |  |  |  |
| Pr032284672 | BP-059 | (AG) _10_ | GGGGAGAGACATAGCAATGCTC | 62 | 129 | 0 | 0 | 0 | 0 | 0 |  |
|  |  |  | CCCTCGCTGCCTCTTCATTTAG | 62 |  |  |  |  |  |  |  |
| Pr032284673 | BP-060 | (AG) _10_ | GTGCTCGTTCCTACACCCATTTTC | 62 | 339 | 0 | 0 | 0 | 0 | 0 |  |
|  |  |  | CATTGACCATATTGCCCTTACCGC | 62 |  |  |  |  |  |  |  |
| Pr032284674 | BP-061 | (AG) _10_ | CGAGTCTCAGACAGACAGGAAGAG | 64 | 206 | 206 | 206 | 215 | 206 | 215 | 5--8 |
|  |  |  | GTGAACTTGGGAAGTCACCCGTC | 64 |  |  |  |  |  |  |  |
| Pr032284675 | BP-062 | (AG) _10_ | GCTGAGGAAGAAGAACACTGTACC | 62 | 172 | 175 | 175 | 175 | 175 | 175 | 4--8 |
|  |  |  | CTCTCTCTTGTTGCTTTCTCGCAC | 62 |  |  |  |  |  |  |  |
| Pr032284676 | BP-063 | (AG) _10_ | CACGTGCAGTGGATCGATAATC | 60 | 134 | 160 | 134 | 160 | 160 | 160 | 5--8 |
|  |  |  | GATCCACAGAGAGAATTCAGGC | 60 |  |  |  |  |  |  |  |
| Pr032284677 | BP-064 | (GA) _10_ | CACCCAGATCATCAAACTGGACC | 62 | 274 | 275 | 275 | 275 | 275 | 275 | 6--6 |
|  |  |  | GAAACTCTCCCAACCCATGACACC | 64 |  |  |  |  |  |  |  |
| Pr032284678 | BP-065 | (GA) _10_ | GAGGATCCAATGCGGGAATGAAG | 62 | 224 | 235 | 235 | 235 | 235 | 224 | 6--6 |
|  |  |  | CCCCAAGGACTGTCTTTGGTGAC | 64 |  |  |  |  |  |  |  |
| Pr032284679 | BP-066 | (GA) _10_ | GGGTTTTTATGATGGGTTCGGG | 60 | 213 | 230 | 230 | 230 | 230 | 215 | 2--3 |
|  |  |  | GGAGTACATCTGGGTGCCCAATCC | 65 |  |  |  |  |  |  |  |
| Pr032284680 | BP-067 | (GA) _10_ | GAGCCTGAGAGATGATTTGCAG | 60 | 160 | 160 | 160 | 160 | 175 | 175 | 1--3 |
|  |  |  | CTGGAAAAATCCAACCCCACCG | 62 |  |  |  |  |  |  |  |
| Pr032284681 | BP-068 | (GA) _10_ | GCCACCAATGCGAAGTAAGCAAGC | 64 | 213 | 215 | 215 | 215 | 215 | 215 | 2--2 |
|  |  |  | CATTCCCTGACATCAACGCCAGC | 64 |  |  |  |  |  |  |  |
| Pr032284682 | BP-069 | (GA) _10_ | GGATTGAGGGAATGCGGGATTGAG | 64 | 191 | 191 | 191 | 185 | 191 | 185 | 4--7 |
|  |  |  | CAATGGGGTGATAGTTTGAGAGGG | 62 |  |  |  |  |  |  |  |
| Pr032284683 | BP-070 | (GA) _10_ | CCGGGCTTGATTCAAGAAGCC | 62 | 238 | 0 | 0 | 0 | 0 | 0 |  |
|  |  |  | CAAACTCCCCCCCTTAAACCTG | 62 |  |  |  |  |  |  |  |
| Pr032284684 | BP-071 | (GA) _10_ | GCTCAACTCTGGCGGAACCGAACC | 67 | 284 | 285 | 285 | 285 | 300 | 285 | 4--9 |
|  |  |  | CCCGTCTAAACTCGGCGATGTTCTC | 65 |  |  |  |  |  |  |  |
| Pr032284685 | BP-072 | (GA) _10_ | GCCGAACATGAAACCGTACCTG | 62 | 291 | 290 | 290 | 300 | 290 | 290 |  |
|  |  |  | CCATGTTTGGTTCCCGAGAAACC | 62 |  |  |  |  |  |  |  |
| Pr032284686 | BP-073 | (GA) _10_ | GGCTTACTCGGGCGCCATGCTTGAG | 68 | 178 | 175 | 180 | 175 | 175 | 175 | 2--3 |
|  |  |  | GGTCCCTTAGGGCGTCTCCTCAGC | 69 |  |  |  |  |  |  |  |
| Pr032284687 | BP-074 | (GA) _10_ | GTGGCTTGAGAATTAGCCCAAG | 60 | 281 | 0 | 0 | 0 | 0 | 0 |  |
|  |  |  | GTGAGAAATGGTGGCGTAGAGG | 62 |  |  |  |  |  |  |  |
| Pr032284688 | BP-075 | (GA) _10_ | GCTTGAGTGCCACGAATTTGTCAC | 62 | 221 | 220 | 220 | 230 | 220 | 230 | 3--5 |
|  |  |  | GGGATGGTAGTTTGAGGGATCTG | 62 |  |  |  |  |  |  |  |
| Pr032284689 | BP-076 | (GA) _10_ | GAAAGGGGAAAGGGAGTTGGGGATC | 65 | 210 | 215 | 210 | 215 | 215 | 210 | 4--8 |
|  |  |  | GTCCCAAGCATTATTGGCGGTGGC | 65 |  |  |  |  |  |  |  |
| Pr032284690 | BP-077 | (GA) _10_ | GTGTATGAAGAGAACATGCGGTGG | 62 | 254 | 250 | 250 | 250 | 250 | 250 | 6--6 |
|  |  |  | AAGACCCCATACGTTCTACCCTC | 62 |  |  |  |  |  |  |  |
| Pr032284691 | BP-078 | (GA) _10_ | GAACCTCAATCCATCGCATACC | 60 | 182 | 185 | 180 | 180 | 185 | 185 | 3--7 |
|  |  |  | GTCTTGAAGGCGAAACCACCTC | 62 |  |  |  |  |  |  |  |
| Pr032284692 | BP-079 | (GA) _10_ | GTTGTTGAGCGTCTCGAACTTGAG | 62 | 302 | 305 | 302 | 305 | 305 | 305 | 2--5 |
|  |  |  | CGCGAAGTTTGACTAAGACCTCTC | 62 |  |  |  |  |  |  |  |
| Pr032284693 | BP-080 | (GA) _10_ | CTGGTCAGAGGATCAGATGGTG | 62 | 249 | 250 | 250 | 250 | 250 | 250 | 2--2 |
|  |  |  | CGGCCAGAGTTCATCTGATTTG | 60 |  |  |  |  |  |  |  |
| Pr032284694 | BP-081 | (GA) _10_ | GAATCCCACAGTTTCTCCGGTTG | 62 | 236 | 230 | 230 | 225 | 230 | 230 | 4--9 |
|  |  |  | GCTGTTCTTGAATCTTGACCAGGC | 62 |  |  |  |  |  |  |  |
| Pr032284695 | BP-082 | (GA) _10_ | GAGAACAGCCCTGAGATTGAGG | 62 | 188 | 0 | 0 | 0 | 0 | 0 |  |
|  |  |  | CAAGGGCAATCGCGTGATTGTAC | 62 |  |  |  |  |  |  |  |
| Pr032284696 | BP-083 | (GA) _10_ | GTCCTGCCTTGAAGGAATTTCTCG | 62 | 184 | 185 | 185 | 185 | 185 | 185 | 3--7 |
|  |  |  | CTTGAGTGTGTTGGCATTAGGGTG | 62 |  |  |  |  |  |  |  |
| Pr032284697 | BP-084 | (GA) _10_ | GTTCGAGCCCGACTCATGTTCG | 64 | 284 | 285 | 285 | 285 | 285 | 285 | 3--3 |
|  |  |  | GTCCAGATTTCCGGCCTGTTAG | 62 |  |  |  |  |  |  |  |
| Pr032284698 | BP-085 | (GA) _10_ | CCCAAAGAAAAGACCTCCGCAGTG | 64 | 261 | 260 | 260 | 250 | 260 | 260 | 3--5 |
|  |  |  | GTTTGCTCGTGAGAGGAACATACC | 62 |  |  |  |  |  |  |  |
| Pr032284699 | BP-086 | (GA) _10_ | CTGGGCTGTAGTGACCTCCATATC | 64 | 265 | 250 | 250 | 250 | 250 | 250 | 2--7 |
|  |  |  | GCGTCGGATGGTTTCAATGCTTGC | 64 |  |  |  |  |  |  |  |
| Pr032284700 | BP-087 | (GA) _10_ | CTCTGTTGAGATCGCCTTTCGTCG | 64 | 271 | 0 | 0 | 0 | 0 | 0 |  |
|  |  |  | CAGCGCACGTCTTATCACAAACC | 62 |  |  |  |  |  |  |  |
| Pr032284701 | BP-088 | (GA) _10_ | CGTCGAACAGGTTATGGTGTTC | 60 | 194 | 200 | 200 | 200 | 200 | 200 | 2--2 |
|  |  |  | CACTTCTCTTCTTCCTCCAACC | 60 |  |  |  |  |  |  |  |
| Pr032284702 | BP-089 | (GA) _10_ | GAAGTTGGCCATGGCCATGAAAG | 62 | 167 | 165 | 165 | 180 | 165 | 165 | 4--8 |
|  |  |  | CTCCTTGTTCCTCCTCCTCATTG | 62 |  |  |  |  |  |  |  |
| Pr032284703 | BP-090 | (GA) _10_ | GCCAGCATGTGAGAATAACGAACC | 62 | 244 | 245 | 245 | 245 | 245 | 245 | 3--3 |
|  |  |  | CTTGATTGGCCTGATGCGATAACC | 62 |  |  |  |  |  |  |  |
| Pr032284704 | BP-091 | (GA) _10_ | CGATTGAGCGAAGTTGATGTTCGC | 62 | 313 | 0 | 0 | 0 | 0 | 0 |  |
|  |  |  | CCAGTTGGGAAGCTGTTATAGACC | 62 |  |  |  |  |  |  |  |
| Pr032284705 | BP-092 | (GA) _10_ | GCACATATGAAGCCCTAGTGTG | 60 | 195 | 195 | 195 | 195 | 195 | 195 | 4--4 |
|  |  |  | CCTGTTCTAACTCTTGGGTCTC | 60 |  |  |  |  |  |  |  |
| Pr032284706 | BP-093 | (GA) _10_ | GGTAGTGGGTTCCTGAAGCATG | 62 | 222 | 225 | 225 | 225 | 225 | 225 |  |
|  |  |  | CTACCTCGCTTACATCCTCCTC | 62 |  |  |  |  |  |  |  |
| Pr032284707 | BP-094 | (GA) _10_ | GGCTAACCTGATGAGAGTGCTC | 62 | 274 | 275 | 275 | 275 | 275 | 275 | 6--6 |
|  |  |  | CTACACCCAAAAGGCTAGCTGC | 62 |  |  |  |  |  |  |  |
| Pr032284708 | BP-095 | (GA) _10_ | GGCCTCTTCAAACCTCACTAACC | 62 | 166 | 166 | 166 | 166 | 166 | 166 | 3--9 |
|  |  |  | CATGTTCGGACACGCCTTTACTTC | 62 |  |  |  |  |  |  |  |
| Pr032284709 | BP-096 | (GA) _10_ | GTATGGCCATCCTCACATCAATGC | 62 | 315 | 0 | 0 | 0 | 0 | 0 |  |
|  |  |  | CCCCACCAAGCAATTCACTTTG | 60 |  |  |  |  |  |  |  |
| Pr032284710 | BP-097 | (GA) _10_ | CTCCCATGAGAATCTCTGCACTG | 62 | 175 | 175 | 175 | 200 | 200 | 200 | 4--4 |
|  |  |  | GCGTGTTATTGGGAGAAAAGGAGC | 62 |  |  |  |  |  |  |  |
| Pr032284711 | BP-098 | (GA) _10_ | CACAGAATGCTCCTTTGATGCGAC | 62 | 201 | 200 | 200 | 200 | 200 | 200 | 3--8 |
|  |  |  | CGAGAGTTAGTGATGGAACGAAGC | 62 |  |  |  |  |  |  |  |
| Pr032284712 | BP-099 | (GA) _10_ | GTATGCTAAGCACCTGAAAGAGGCG | 64 | 283 | 285 | 285 | 285 | 285 | 285 | 5--5 |
|  |  |  | CATCAGACAAGCAGTTGGCTTCCTG | 64 |  |  |  |  |  |  |  |
| Pr032284713 | BP-100 | (GA) _10_ | GACAGCAGCCAAATAGCTAGCTAG | 62 | 174 | 175 | 175 | 175 | 175 | 175 | 4--4 |
|  |  |  | CTACATATCCCGTTCCCCCTGTTAC | 64 |  |  |  |  |  |  |  |
| Pr032284714 | BP-101 | (GA) _10_ | CTTGGCCACCGAAAATGATGCC | 62 | 197 | 200 | 200 | 200 | 200 | 200 | 2--2 |
|  |  |  | GCATACGGCATATCGTAGCAAG | 60 |  |  |  |  |  |  |  |
| Pr032284715 | BP-102 | (GA) _10_ | CTCAGCAACCATACAGGAGGTAC | 62 | 141 | 140 | 140 | 150 | 140 | 140 | 5--8 |
|  |  |  | CAGAAGCCGAAAGAAAGCGTAG | 60 |  |  |  |  |  |  |  |
| Pr032284716 | BP-103 | (GA) _10_ | GGGTTGTGGTTGTTGCCAATGTTG | 62 | 236 | 240 | 240 | 240 | 240 | 240 | 6--6 |
|  |  |  | GCTGGTTGAAGGAAATTTCGGAGG | 62 |  |  |  |  |  |  |  |
| Pr032284717 | BP-104 | (GA) _10_ | CATGGGCATTTGATGGGTGCTTG | 62 | 164 | 0 | 0 | 0 | 0 | 0 |  |
|  |  |  | GTATAAACAGGGGAACAGCGTC | 60 |  |  |  |  |  |  |  |
| Pr032284718 | BP-105 | (GA) _10_ | CACCGATTTCTCAAACACTGCCAG | 62 | 215 | 215 | 215 | 215 | 215 | 215 | 4--4 |
|  |  |  | CACAAAAAGCAAGGGCAGTATCGG | 62 |  |  |  |  |  |  |  |
| Pr032284719 | BP-106 | (GA) _10_ | GTTCTGTGGCAGTAATGGGTGGAG | 62 | 179 | 0 | 0 | 0 | 0 | 0 |  |
|  |  |  | CGTGCTTAGCTGAACCATGGTTAC | 62 |  |  |  |  |  |  |  |
| Pr032284720 | BP-107 | (GA) _10_ | GCGGAAGGTATATGTGGTGGAATG | 62 | 169 | 169 | 169 | 169 | 169 | 169 | 3--5 |
|  |  |  | GATGTCTCGTACTGGAGAAGAGTC | 62 |  |  |  |  |  |  |  |
| Pr032284721 | BP-108 | (GA) _10_ | CTAAATACGAAGCCCCTCCCAAG | 62 | 129 | 130 | 130 | 130 | 130 | 130 | 2--7 |
|  |  |  | GGGGATGATTACATGCATCTCCC | 62 |  |  |  |  |  |  |  |
| Pr032284722 | BP-109 | (GA) _10_ | GTTGACGGCGTCGAGGGAGATCTAC | 67 | 199 | 200 | 200 | 200 | 200 | 200 | 5--5 |
|  |  |  | CACTTCATGCACGGCATGGACACGC | 67 |  |  |  |  |  |  |  |
| Pr032284723 | BP-110 | (GA) _10_ | GAGCGAGATTTGGTGGTCATACC | 62 | 177 | 180 | 180 | 180 | 175 | 175 | 3--7 |
|  |  |  | GTGGAGTAATGCCCACCTTATGC | 62 |  |  |  |  |  |  |  |
| Pr032284724 | BP-111 | (GA) _10_ | GGCCAGGAGCAAGAAGAGAGAAAG | 64 | 118 | 118 | 118 | 135 | 118 | 135 | 4--8 |
|  |  |  | CTTCCCACTTCCCACATCCTCTTC | 64 |  |  |  |  |  |  |  |
| Pr032284725 | BP-112 | (AG)_16_ | CGGGAAGATATGCAGTGTTT | 56 | 227 | 225 | 225 | 225 | 225 | 225 | 4--4 |
|  |  |  | TTGGCGGGTGAAGTAGAC | 57 |  |  |  |  |  |  |  |
| Pr032284726 | BP-113 | (CT)_13_A(TC)_6_ | CACACTGCTGCCTGA | 54 | 168 | 170 | 170 | 170 | 170 | 170 | 2--6 |
|  |  |  | TCATAAAACCCTCAAAGAAT | 50 |  |  |  |  |  |  |  |
| Pr032284727 | BP-114 | (AG)_4_AA(AG)_11_ | TTTCCAACGCTTTCTTGATG | 54 | 170 | 0 | 0 | 0 | 0 | 0 | 5--9 |
|  |  |  | TGGATAAGGAAGGGCATGTC | 58 |  |  |  |  |  |  |  |
| Pr032284728 | BP-115 | (CT)_14_ | TCTACGCTGTGACCAGTC | 57 | 187 | 185 | 200 | 185 | 200 | 200 | 3--4 |
|  |  |  | AGAATCCTAGCCTTTTCAAT | 52 |  |  |  |  |  |  |  |
| Pr032284729 | BP-116 | (TC)_13_ | AATGCAGCATCTCTTACC | 53 | 139 | 150 | 150 | 139 | 150 | 150 | 3--8 |
|  |  |  | CACGCAATAATATGGAAA | 48 |  |  |  |  |  |  |  |
| Pr032284730 | BP-117 | (TC)_14_ | TGGCAGCACGAAAGT | 51 | 194 | 200 | 200 | 200 | 200 | 200 | 5--5 |
|  |  |  | TGGGAATGAGAGAACAAG | 53 |  |  |  |  |  |  |  |
| Pr032284731 | BP-118 | (TC)_16_(AC)_5_ | TGTGTGGCTCCTTATTCTTA | 54 | 138 | 0 | 0 | 0 | 0 | 0 |  |
|  |  |  | GGCAACAAATTATGAGGTAG | 54 |  |  |  |  |  |  |  |
| Pr032284732 | BP-119 | (GT)_12_... (GA)_5_ | GGCAACCAGCAGCAATCTGAC | 56 | 156 | 0 | 0 | 0 | 0 | 0 |  |
|  |  |  | ATGCCCAAGGACGACTAGACC | 56 |  |  |  |  |  |  |  |
| Pr032284733 | BP-120 | (GT)_10_(GA)_12_ | TCTCAGTTTCCAAGAAGCACG | 54 | 138 | 0 | 0 | 0 | 0 | 0 |  |
|  |  |  | TCAGTCACATCATTTTTAGCC | 54 |  |  |  |  |  |  |  |
| Pr032284734 | BP-121 | A_6_TA_8_...(TA)_13_ | CCTTGTGTACTTGAGTAGTGC | 54 | 152 | 150 | 150 | 150 | 150 | 150 | 1--2 |
|  |  |  | TTGATCCCACCAGTTTATTGC | 54 |  |  |  |  |  |  |  |
| Pr032284735 | BP-122 | (AG) _6_ | GAATGTTCTCTGCTCCTCCAG | 60 | 197 | 0 | 0 | 0 | 0 | 0 |  |
|  |  |  | TCACTATTCGGTGCAACAGG | 58 |  |  |  |  |  |  |  |
| Pr032284736 | BP-123 | (CT) _6_ | TCTCACCAAACCACTCACTCA | 58 | 215 | 225 | 123 | 123 | 225 | 225 | 2--3 |
|  |  |  | AAGAGCGTGGCAATGAACTC | 58 |  |  |  |  |  |  |  |
| Pr032284737 | BP-124 | (TA) _5_ | CAGACGACAAAGCAAGCTGA | 58 | 213 | 124 | 150 | 150 | 124 | 124 | 3--5 |
|  |  |  | CATGCTCACATACAAGGCAAA | 56 |  |  |  |  |  |  |  |
| Pr032284738 | BP-125 | (CT) _8_ | GGTTGCTCAACCTAACCAACA | 58 | 215 | 215 | 215 | 215 | 215 | 215 | 4--4 |
|  |  |  | AGAACACCCACCAAGTCACC | 60 |  |  |  |  |  |  |  |
| Pr032284739 | BP-126 | (AGCG) _5_ | GCCGGGAGAATTACACGTC | 60 | 242 | 245 | 245 | 245 | 245 | 245 | 3--3 |
|  |  |  | CCCCTTTCTTCAGATCAACG | 58 |  |  |  |  |  |  |  |
| Pr032284740 | BP-127 | (AG) _6_ | GAGAGAACCAAAACAGTAGACAGAGA | 60 | 168 | 165 | 165 | 165 | 180 | 165 | 3--6 |
|  |  |  | GGCCTGTTCTTGATGACGAT | 58 |  |  |  |  |  |  |  |
| Pr032284741 | BP-128 | (AG) _10_ | GGGGGTTGCTCTTCATTTTT | 56 | 222 | 128 | 128 | 128 | 135 | 128 | 1--3 |
|  |  |  | GGTTTCCTCGTCGGTTATGA | 58 |  |  |  |  |  |  |  |
| Pr032284742 | BP-129 | (CT) _7_ | CAGCGTATGAAACCAGAACG | 58 | 189 | 0 | 0 | 0 | 0 | 0 |  |
|  |  |  | TAAAACGGACCCACTTGAGC | 58 |  |  |  |  |  |  |  |
| Pr032284743 | BP-130 | (AT) _5_ | GTTAAGAAGGTGCGCCAGTC | 60 | 254 | 250 | 255 | 255 | 255 | 255 | 2--5 |
|  |  |  | ACTAACCGCGCATAAACTGC | 58 |  |  |  |  |  |  |  |
| Pr032284744 | BP-131 | (CT) _13_ | GTTGCTGCTCACCTCAAAAATGT | 58 | 195 | 200 | 200 | 200 | 200 | 200 | 3--3 |
|  |  |  | TGCACGGTTGGAGAATAGAAGAA | 58 |  |  |  |  |  |  |  |
| Pr032284745 | BP-132 | (AT) _16_ | GCCTGGAACCATCAAGAAGA | 58 | 231 | 0 | 0 | 0 | 0 | 0 |  |
|  |  |  | TCCACCTCATCAACCCTCTC | 60 |  |  |  |  |  |  |  |
| Pr032284746 | BP-133 | (AAAT) _5_ | CTAAATTGGACCCGACTAACCCG | 62 | 141 | 140 | 150 | 150 | 150 | 150 | 3--5 |
|  |  |  | GGCCTCGTTCAGTTTCGTCTCCG | 66 |  |  |  |  |  |  |  |
| Pr032284747 | BP-134 | (AAAT) _5_ | CTGAACCTGGTAACCCTTTCCC | 62 | 132 | 140 | 140 | 140 | 130 | 140 | 3--6 |
|  |  |  | GGGAATTCTCTCTCAGGAGTTG | 60 |  |  |  |  |  |  |  |
| Pr032284748 | BP-135 | (AAAT) _5_ | GCTCATCGCGACCATCTAATGAAG | 62 | 219 | 220 | 220 | 220 | 220 | 220 | 2--3 |
|  |  |  | CTTGAGTGGTTGTCCTCTTTGGTC | 62 |  |  |  |  |  |  |  |
| Pr032284749 | BP-136 | (AAAT) _5_ | CTCCTACCTCCTCTTCCGCAAC | 64 | 160 | 160 | 160 | 160 | 160 | 160 | 2--4 |
|  |  |  | GAAACTGTATTTTGGGTGGGGG | 60 |  |  |  |  |  |  |  |
| Pr032284750 | BP-137 | (AAAT) _5_ | CCCAGCAGCAGAAGTGCCTAGT | 64 | 189 | 190 | 190 | 190 | 190 | 190 | 3--5 |
|  |  |  | CCTTATCCGTGAACTTCACCTG | 60 |  |  |  |  |  |  |  |
| Pr032284751 | BP-138 | (AAAT) _5_ | CGCTTCCAACTTAACTCTCACG | 60 | 205 | 205 | 205 | 205 | 205 | 205 | 2--3 |
|  |  |  | TCTATCTCTTCTTGTGCCGACG | 60 |  |  |  |  |  |  |  |
| Pr032284752 | BP-139 | (AAAT) _5_ | CCATTACCACCGGCACAACCTA | 62 | 197 | 195 | 200 | 195 | 200 | 195 | 2--4 |
|  |  |  | TCGATTTCATGAGCCTGTCCTG | 60 |  |  |  |  |  |  |  |
| Pr032284753 | BP-140 | (AAAT) _5_ | CCCACATGCCTGTCGGTGACTC | 66 | 163 | 163 | 163 | 163 | 163 | 163 | 2--4 |
|  |  |  | AGGTGTGCGGAGTGTACTCATG | 62 |  |  |  |  |  |  |  |
| Pr032284754 | BP-141 | (AAAT) _5_ | CGTCGTCAGCTCGTGGAGTCTGC | 67 | 177 | 175 | 175 | 175 | 175 | 190 | 1--4 |
|  |  |  | CTAATGGGTTCACGCGGGATCCG | 66 |  |  |  |  |  |  |  |
| Pr032284755 | BP-142 | (AAAT) _5_ | CATCCCTGCCTAGAACTGATCT | 60 | 208 | 205 | 205 | 205 | 210 | 205 | 2--6 |
|  |  |  | TTGCTCTGCAATGTTGGGCATC | 60 |  |  |  |  |  |  |  |
| Pr032284756 | BP-143 | (AAAT) _5_ | CCCAATGCTGCTGTAAAGGGTG | 62 | 222 | 225 | 225 | 225 | 225 | 225 | 1--3 |
|  |  |  | GTCTCTGCCATGTCCTTGTTGG | 62 |  |  |  |  |  |  |  |
| Pr032284757 | BP-144 | (AAAT) _5_ | CTCCAATGGTGACAGGGGAGAA | 62 | 268 | 265 | 265 | 265 | 265 | 265 | 3--6 |
|  |  |  | AGTGAAGGCTCCAGTTGTTTCG | 60 |  |  |  |  |  |  |  |
| Pr032284758 | BP-145 | (AAAT) _5_ | CAACGGGGGTTGCTAAGCCACCTC | 67 | 197 | 195 | 195 | 195 | 195 | 195 | 3--6 |
|  |  |  | AACCACCCCCCAAATGGGTAGGGG | 67 |  |  |  |  |  |  |  |
| Pr032284759 | BP-146 | (AAAT) _5_ | CTGCGGTTCGCGCTGTAAGATA | 62 | 199 | 200 | 200 | 200 | 200 | 200 | 2--5 |
|  |  |  | GTTCGATGCAAAGAACGGCTTG | 60 |  |  |  |  |  |  |  |
| Pr032284760 | BP-147 | (AAAT) _5_ | CCTCCTCTTCAAAGAGAGGACC | 62 | 230 | 230 | 230 | 230 | 230 | 230 | 3--6 |
|  |  |  | AATGTTGGTGTTCGTGGGCTGC | 62 |  |  |  |  |  |  |  |
| Pr032284761 | BP-148 | (AAAT) _5_ | GAATTGGGGGAAAGTCCGACG | 62 | 213 | 215 | 215 | 215 | 215 | 215 | 2--5 |
|  |  |  | GGAGTTGGATAGATGGGTTGG | 60 |  |  |  |  |  |  |  |
| Pr032284762 | BP-149 | (AAAT) _5_ | CAATCCACAGGCCACTGTACGTCA | 64 | 187 | 185 | 185 | 185 | 185 | 185 | 2--4 |
|  |  |  | CCTCCCAGCTATAAATGCAAGACG | 62 |  |  |  |  |  |  |  |
| Pr032284763 | BP-150 | (AAAT) _5_ | CCGCCACCTCCTTGTCATCAACGT | 65 | 234 | 235 | 235 | 235 | 235 | 235 | 2--3 |
|  |  |  | CTTAAGCGGCGGTCCAAACAACGG | 65 |  |  |  |  |  |  |  |
| Pr032284764 | BP-151 | (AAAT) _5_ | CTGTTCACTCACTATGCCCGTT | 60 | 204 | 205 | 205 | 210 | 210 | 210 | 2--5 |
|  |  |  | AATGGCAGGTTCTGAAACCACG | 60 |  |  |  |  |  |  |  |
| Pr032284765 | BP-152 | (AAAT) _5_ | CTTCCTGTGTACCTAGGGGTGCCT | 65 | 172 | 175 | 175 | 175 | 175 | 175 | 2--3 |
|  |  |  | GTCCCAGAACCTAGGGTCAGCAAG | 65 |  |  |  |  |  |  |  |
| Pr032284766 | BP-153 | (AAAT) _5_ | CCGAAGCATCACAAAGCCTTGA | 60 | 180 | 180 | 180 | 180 | 180 | 180 | 2--3 |
|  |  |  | ACGAATTAGGCCAGTCAGAAGG | 60 |  |  |  |  |  |  |  |
| Pr032284767 | BP-154 | (AAAT) _5_ | CGTCAAACGGCCTCATCCAACCAA | 64 | 157 | 160 | 160 | 170 | 170 | 170 | 3--5 |
|  |  |  | TATCGTTGCATGACACTGGGGCAG | 64 |  |  |  |  |  |  |  |
| Pr032284768 | BP-155 | (AAAT) _5_ | GTGGGCAAGTGCATTAAAGGCACG | 64 | 144 | 145 | 145 | 145 | 145 | 145 | 2--5 |
|  |  |  | GGTGGCCATTTCTTGACTGGTGTG | 64 |  |  |  |  |  |  |  |
| Pr032284769 | BP-156 | (AAAT) _5_ | CTCCAATCTTCTCATGTGCAGGC | 62 | 205 | 205 | 205 | 205 | 205 | 205 | 2--4 |
|  |  |  | AGAATGACTTGGTCCATGGGTGG | 62 |  |  |  |  |  |  |  |
| Pr032284770 | BP-157 | (AAAT) _5_ | CCAAGTTCCCACTCCAAAGGGC | 64 | 210 | 210 | 210 | 210 | 210 | 210 | 2--5 |
|  |  |  | GTGCCCTTGCAGTTGCACAGAG | 64 |  |  |  |  |  |  |  |
| Pr032284771 | BP-158 | (AAAT) _5_ | CCAACCTCCAACCTCAGACCTCT | 64 | 235 | 235 | 235 | 235 | 250 | 250 | 3--5 |
|  |  |  | TTGCTTGACTCCCGTCCTTTGCC | 64 |  |  |  |  |  |  |  |
| Pr032284772 | BP-159 | (AAAT) _5_ | CTGGGTTCCCATCCTAATCGGT | 62 | 216 | 215 | 215 | 215 | 215 | 215 | 2--6 |
|  |  |  | GCTAGGCCCTTTGCCATGAAAG | 62 |  |  |  |  |  |  |  |
| Pr032284773 | BP-160 | (AAAT) _5_ | CCACAGGACACATCAGAGGTAGAA | 62 | 235 | 235 | 235 | 235 | 235 | 235 | 2--5 |
|  |  |  | CCTAACCCTAGTTGAACCAGCAAC | 62 |  |  |  |  |  |  |  |
| Pr032284774 | BP-161 | (AAT) _8_ | GTATCCCGTGCTTTGTGCAGGAAT | 62 | 198 | 200 | 200 | 200 | 200 | 200 | 3--5 |
|  |  |  | CTTGACCACGTTTGGAAGGTTTGG | 62 |  |  |  |  |  |  |  |
| Pr032284775 | BP-162 | (AAT) _8_ | CATCCAGAAGCAGGGCCAAGTCA | 64 | 215 | 215 | 215 | 215 | 215 | 220 | 2--7 |
|  |  |  | CATCACCGATTATGCGACTGCGG | 64 |  |  |  |  |  |  |  |
| Pr032284776 | BP-163 | (AAT) _8_ | GACCGAGATTCCATTACACGCA | 60 | 161 | 160 | 160 | 160 | 160 | 225 | 1--4 |
|  |  |  | CAGTCCAACTTTAGTTAGCGCG | 60 |  |  |  |  |  |  |  |
| Pr032284777 | BP-164 | (AAT) _8_ | CACGGTGGCGTTTAATCCAACA | 60 | 250 | 250 | 250 | 250 | 250 | 250 | 1--3 |
|  |  |  | TTTGCTCCTGGTTTGGTCGATG | 60 |  |  |  |  |  |  |  |
| Pr032284778 | BP-165 | (AAT) _8_ | CCCTCACATGTTTACCCTGAAC | 60 | 150 | 150 | 150 | 150 | 150 | 150 | 1--4 |
|  |  |  | AAGCCACCGTCATACAAGATGC | 60 |  |  |  |  |  |  |  |
| Pr032284779 | BP-166 | (AAT) _8_ | CCATGATCTCCACCCAGATCAA | 60 | 177 | 175 | 175 | 175 | 175 | 175 | 2--4 |
|  |  |  | AGACTGTTGTGTCTCGTAGTCG | 60 |  |  |  |  |  |  |  |
| Pr032284780 | BP-167 | (AAT) _8_ | CCTCCACCACACACAAAGAAAG | 60 | 218 | 220 | 215 | 215 | 215 | 215 | 3--6 |
|  |  |  | GTTTGGAATGGAGGACTCAAGG | 60 |  |  |  |  |  |  |  |
| Pr032284781 | BP-168 | (AAT) _8_ | CGTATGGGAGTGTTAAACCCAG | 60 | 159 | 160 | 165 | 165 | 160 | 165 | 3--5 |
|  |  |  | GATGTTCACTATGCCACTACGG | 60 |  |  |  |  |  |  |  |
| Pr032284782 | BP-169 | (AAT) _8_ | CAGCTCTACGCGAAAGTATACC | 60 | 197 | 200 | 200 | 200 | 200 | 205 | 2--6 |
|  |  |  | AATTGAATGTCGCGTGCATGGG | 60 |  |  |  |  |  |  |  |
| Pr032284783 | BP-170 | (AAT) _8_ | CTGGCCGTAGACAAGGGTCTTTT | 62 | 150 | 150 | 150 | 150 | 150 | 155 | 2--4 |
|  |  |  | CCCTTTTCCCTTAACTTCCTCCG | 62 |  |  |  |  |  |  |  |
| Pr032284784 | BP-171 | (AAT) _8_ | CCCCCTCACCCCCAAAAAAAAA | 60 | 164 | 165 | 165 | 165 | 165 | 165 | 6--9 |
|  |  |  | GAAAGATCAAAGCCGCTACACG | 60 |  |  |  |  |  |  |  |
| Pr032284785 | BP-172 | (AAT) _8_ | CAACTTTGTGCCATAGAGACGC | 60 | 169 | 170 | 170 | 170 | 170 | 170 | 3--8 |
|  |  |  | GGGGACATTGTCAATTGAGTGG | 60 |  |  |  |  |  |  |  |
| Pr032284786 | BP-173 | (AAT) _8_ | GGGCCAATCCAATCCACATCTT | 60 | 230 | 230 | 230 | 230 | 230 | 230 | 3--5 |
|  |  |  | CAAAAGGGTTACTGGGGATGAG | 60 |  |  |  |  |  |  |  |
| Pr032284787 | BP-174 | (AAT) _8_ | CATCATGGGCCATGTCCTGAAA | 60 | 179 | 175 | 175 | 175 | 180 | 175 | 2--6 |
|  |  |  | GGCTACCATGATAATCTTGCCC | 60 |  |  |  |  |  |  |  |
| Pr032284788 | BP-175 | (AAT) _8_ | CCCTCCCTTCCGTTCACAGATT | 62 | 200 | 205 | 205 | 200 | 200 | 205 | 5--6 |
|  |  |  | TTGCAGAGAGGGAGTTGGAGAC | 62 |  |  |  |  |  |  |  |
| Pr032284789 | BP-176 | (AAT) _8_ | CTCCATAGCAAAAGGCGGGCCA | 64 | 166 | 165 | 165 | 165 | 165 | 165 | 4--7 |
|  |  |  | TTCGGCCACTCCCAAAGGGATG | 64 |  |  |  |  |  |  |  |
| Pr032284790 | BP-177 | (AC) _10_ | CCACCCCATTAATCTATGGCAACC | 62 | 165 | 165 | 165 | 165 | 165 | 165 | 4--9 |
|  |  |  | ATCGGCACATGCAACAAGCTTCAC | 62 |  |  |  |  |  |  |  |
| Pr032284791 | BP-178 | (AC) _10_ | CTCTAAAGCCCAAAAGCCTCCT | 60 | 193 | 195 | 195 | 195 | 195 | 195 | 4--6 |
|  |  |  | TCGTACCACCGACTGTTCAATG | 60 |  |  |  |  |  |  |  |
| Pr032284792 | BP-179 | (AC) _10_ | CTGGTAGGGCAAGCCTTGCACT | 64 | 204 | 205 | 205 | 205 | 205 | 205 | 4--5 |
|  |  |  | ATGCTGTATGGGAGGTCCCGTG | 64 |  |  |  |  |  |  |  |
| Pr032284793 | BP-180 | (AC) _10_ | CAGTGCCACCAAGAGACCAAAT | 60 | 210 | 210 | 210 | 210 | 210 | 210 | 4--5 |
|  |  |  | TAATTGTGTGCGCGTCTGTGTG | 60 |  |  |  |  |  |  |  |
| Pr032284794 | BP-181 | (AC) _10_ | CAACCCAAGAATGCATGGGCCT | 62 | 208 | 210 | 210 | 210 | 210 | 210 | 4--6 |
|  |  |  | TACAAAGAGGGGAGGGATGCAG | 62 |  |  |  |  |  |  |  |
| Pr032284795 | BP-182 | (AC) _10_ | CCAGACACTTTCATGGTGGTTG | 60 | 204 | 205 | 205 | 205 | 210 | 205 | 2--4 |
|  |  |  | GGCTTCCATCCAGCAGTTATTG | 60 |  |  |  |  |  |  |  |
| Pr032284796 | BP-183 | (AC) _10_ | CTCACTCTCACTCACACACCAA | 60 | 207 | 205 | 205 | 205 | 205 | 205 | 3--6 |
|  |  |  | TTGATGTCCCCTTGGCATCTTC | 60 |  |  |  |  |  |  |  |
| Pr032284797 | BP-184 | (AC) _10_ | CCTTAAATCGCCTAACCGCCTT | 60 | 227 | 225 | 225 | 225 | 225 | 225 | 1--3 |
|  |  |  | AAAGGGTGGAGCAAGAGTTAGG | 60 |  |  |  |  |  |  |  |
| Pr032284798 | BP-185 | (AC) _10_ | CTATTTCCTCTCCAGCCTCCCC | 64 | 176 | 175 | 180 | 175 | 175 | 175 | 4--9 |
|  |  |  | TGTTGGCGGGATCCTGGGATTG | 64 |  |  |  |  |  |  |  |
| Pr032284799 | BP-186 | (TG) _10_ | CACGGTCACAGGAAGACAGAGC | 64 | 158 | 155 | 155 | 155 | 160 | 155 | 2--6 |
|  |  |  | CCCTCAATATCCGACCCAACCC | 64 |  |  |  |  |  |  |  |
| Pr032284800 | BP-187 | (TG) _10_ | CTGTCAAATCCCTGAAGCATGC | 60 | 176 | 175 | 175 | 175 | 190 | 190 | 2--5 |
|  |  |  | CAGCTCTGTGAAGGATTTGGAG | 60 |  |  |  |  |  |  |  |
| Pr032284801 | BP-188 | (TG) _10_ | CCCAGATATGCACTCTCCATGG | 62 | 194 | 195 | 195 | 195 | 195 | 195 | 3--6 |
|  |  |  | ATGGGGAGAGGGTGCATATCTG | 62 |  |  |  |  |  |  |  |
| Pr032284802 | BP-189 | (AGG)7 | CGGCTATTGGAGGAGAAAGGGA | 62 | 202 | 200 | 200 | 200 | 200 | 200 | 4--8 |
|  |  |  | GTTTCACCACCACCTCCATCAG | 62 |  |  |  |  |  |  |  |
| Pr032284803 | BP-190 | (AGG)7 | CGTCAGGGAGGAAGAGGTTGCG | 66 | 221 | 225 | 225 | 225 | 225 | 225 | 5--6 |
|  |  |  | AGTTCCGACCGAGGACCTCGTC | 66 |  |  |  |  |  |  |  |
| Pr032284804 | BP-191 | (TC) _13_ | GGTTAGTGAATGCCCCTCTCTC | 62 | 191 | 190 | 190 | 190 | 195 | 190 | 2--3 |
|  |  |  | TATAGACCAGAACGCGCTCCAC | 62 |  |  |  |  |  |  |  |
| Pr032284805 | BP-192 | (TC) _13_ | CTCTCTCTCGTCTCCATCTTCT | 60 | 130 | 130 | 130 | 130 | 130 | 175 | 5--7 |
|  |  |  | CAGATGAGAGAGAGAAACGCTG | 60 |  |  |  |  |  |  |  |
| Pr032284806 | BP-193 | (TC) _13_ | CCGTTGTATTACCCCATCTTCACC | 62 | 201 | 200 | 200 | 200 | 200 | 200 | 2--5 |
|  |  |  | ATGTAACCCCCAAGAAGCAGAAGG | 62 |  |  |  |  |  |  |  |
| Pr032284807 | BP-194 | (TC) _13_ | CTCTCCTGCAAGCTGCAACGCC | 66 | 158 | 160 | 160 | 160 | 160 | 160 | 4--6 |
|  |  |  | GTACATGGCGCGCGTGCTACAG | 66 |  |  |  |  |  |  |  |
| Pr032284808 | BP-195 | (TC) _13_ | CCCCACTTACCCTATTTAACGCCC | 64 | 197 | 195 | 200 | 200 | 200 | 200 | 6--7 |
|  |  |  | TGAGGACCCACCTTGGGCATAATC | 64 |  |  |  |  |  |  |  |
| Pr032284809 | BP-196 | (TC) _13_ | CCTCTCTCTCTCTCTCTGTGAT | 60 | 232 | 235 | 235 | 235 | 235 | 235 | 5--7 |
|  |  |  | GTGGATTGTTCATCATGTGGGG | 60 |  |  |  |  |  |  |  |
| Pr032284810 | BP-197 | (TC) _13_ | CTCCGTCTCTCTCATCAACGTC | 62 | 150 | 150 | 150 | 150 | 150 | 150 | 3--3 |
|  |  |  | CGGTGCATGCTTCTCAAAAGGG | 62 |  |  |  |  |  |  |  |
| Pr032284811 | BP-198 | (TC) _13_ | GAGCTGTATGGTCCCCCTATTT | 60 | 178 | 175 | 175 | 175 | 175 | 175 | 7--9 |
|  |  |  | CTGCATTCACAGTAACACGACG | 60 |  |  |  |  |  |  |  |
| Pr032284812 | BP-199 | (TC) _13_ | CGTCCCTCTTTCTCCTACTCCA | 62 | 169 | 165 | 165 | 170 | 165 | 170 | 5--7 |
|  |  |  | TAGGCTGCAAGGCAAGTTGTGG | 62 |  |  |  |  |  |  |  |
| Pr032284813 | BP-200 | (TC) _13_ | CTCAGTCTCTCACAGTCACGAA | 60 | 151 | 150 | 150 | 150 | 150 | 150 | 2--4 |
|  |  |  | TCGGTTAATTCGGTTCAGTCGG | 60 |  |  |  |  |  |  |  |
| Pr032284814 | BP-201 | (TC) _13_ | CAGCGAGGAACCAAACGGACAA | 62 | 195 | 195 | 195 | 195 | 195 | 195 | 3--4 |
|  |  |  | CGTTGATCTCTCTGCGTCTCTG | 62 |  |  |  |  |  |  |  |
| Pr032284815 | BP-202 | (TC) _13_ | CTTTTCATCCGCAGATCCTCTC | 60 | 230 | 235 | 235 | 230 | 235 | 230 | 3--8 |
|  |  |  | CTACCATGCAAGGCTCCATTTG | 60 |  |  |  |  |  |  |  |
| Pr032284816 | BP-203 | (TC) _13_ | CTCTTTCATGGGGTTTGACGCA | 60 | 240 | 245 | 245 | 240 | 245 | 240 | 2--4 |
|  |  |  | CAACAAAGGGCTTTATCCGCTG | 60 |  |  |  |  |  |  |  |
| Pr032284817 | BP-204 | (TC) _13_ | CACCCCAACACCTTATGCAAGT | 60 | 186 | 185 | 185 | 190 | 185 | 190 | 6--7 |
|  |  |  | TCTTGGCGACCTTTAGAAGGAC | 60 |  |  |  |  |  |  |  |
| Pr032284818 | BP-205 | (TC) _13_ | CCCGGCAAGCCACCATGGATTT | 64 | 248 | 245 | 245 | 245 | 245 | 245 | 3--6 |
|  |  |  | CGCTCACTCCCTTCACTCTTGG | 64 |  |  |  |  |  |  |  |
| Pr032284819 | BP-206 | (TC) _13_ | GCGCCACTTGTTTCCTAGCTTT | 60 | 177 | 175 | 190 | 175 | 190 | 175 | 6--8 |
|  |  |  | TAGATTGGATTGTAGGAGGCGG | 60 |  |  |  |  |  |  |  |
| Pr032285987 | BP-207 | (AG)_10_ | CAGCCTTCCTGCCTGCATGTGTG | 66 | 167 | 200 | 200 | 167 | 200 | 167 | 3--9 |
|  |  |  | CGAAGTCAGTTGTCAGCTTGTGG | 62 |  |  |  |  |  |  |  |
| Pr032285988 | BP-208 | (AG)_10_ | GAGCTAGAGAGATGGGTGTGGCAG | 65 | 199 | 200 | 200 | 205 | 200 | 200 | 4--7 |
|  |  |  | CTCGTAACCAGTAACGTACCCACG | 64 |  |  |  |  |  |  |  |
| Pr032285989 | BP-209 | (AG)_10_ | GGTGAGGAGGGAGGTCACAAGG | 66 | 279 | 0 | 0 | 0 | 0 | 0 |  |
|  |  |  | CCCGTCGGATGCATTAGCTGTC | 64 |  |  |  |  |  |  |  |
| Pr032285990 | BP-210 | (AG)_10_ | CCCTCTCCCCATGGTAATTGCATG | 64 | 170 | 170 | 170 | 175 | 170 | 170 | 5--12 |
|  |  |  | GGAGCCTCAAGGCAAGGTAGCTTC | 65 |  |  |  |  |  |  |  |
| Pr032285991 | BP-211 | (AG)_10_ | GAAGATGAGGAAGGGTGGGAATTGG | 64 | 236 | 0 | 0 | 0 | 0 | 0 |  |
|  |  |  | CATCTTCTGTCTTCCTCTGGTAGC | 62 |  |  |  |  |  |  |  |
| Pr032285992 | BP-212 | (AG)_10_ | CACGAGAGAGATCACGCTTTCCC | 64 | 195 | 200 | 200 | 210 | 225 | 210 | 4--10 |
|  |  |  | CCACCGCCAGAAACCCTTTGATC | 64 |  |  |  |  |  |  |  |
| Pr032285993 | BP-213 | (AG)_10_ | CCATTGCTCTCTGAGATAAGGG | 60 | 148 | 210 | 210 | 200 | 200 | 200 | 3--5 |
|  |  |  | GCTCTAACGCTCTCTGACAGTTAC | 62 |  |  |  |  |  |  |  |
| Pr032285994 | BP-214 | (AG)_10_ | CCAAAGCGAAGATGCTCACCGCTTG | 65 | 282 | 285 | 280 | 285 | 280 | 280 | 2--7 |
|  |  |  | CTGTAGGGTTCAAGGGGCGAGAC | 66 |  |  |  |  |  |  |  |
| Pr032285995 | BP-215 | (AG)_10_ | GCTACGATGGTGGTGGTTGGGTGG | 67 | 191 | 190 | 190 | 200 | 190 | 200 | 4--9 |
|  |  |  | CCTCTCTCTCTCTCTCCCTCTCTC | 65 |  |  |  |  |  |  |  |
| Pr032285996 | BP-216 | (AG)_10_ | GGGTAGTAGAGGATGGTGGAAGAG | 64 | 233 | 235 | 235 | 235 | 235 | 235 | 6--6 |
|  |  |  | CTTCCCGGCCACTTTCTCTCGCTC | 67 |  |  |  |  |  |  |  |
| Pr032285997 | BP-217 | (AG)_10_ | CTTTGGCGTCCGTCGTACTAGAG | 64 | 201 | 200 | 200 | 200 | 200 | 200 | 1--3 |
|  |  |  | CCCTTAACCGGTGTCTTTAGAGG | 62 |  |  |  |  |  |  |  |
| Pr032285998 | BP-218 | (AG)_10_ | GACAGCCGTTTTCCACCAGAGC | 64 | 253 | 250 | 250 | 250 | 250 | 250 | 2--2 |
|  |  |  | CCCCTTCTTGGGTCCTCTTTTCC | 64 |  |  |  |  |  |  |  |
| Pr032285999 | BP-219 | (AG)_10_ | GAGGAGAAAAGGGGAATTTGCTGG | 62 | 194 | 190 | 210 | 190 | 190 | 190 | 3--7 |
|  |  |  | CTTCCTCCATGAATGAACGTCCC | 62 |  |  |  |  |  |  |  |
| Pr032286000 | BP-220 | (AG)_10_ | GCCCGACTTGTTCGCACAAAAACC | 64 | 350 | 350 | 350 | 350 | 350 | 350 | 5--5 |
|  |  |  | CTCTTCTCCTCCTCCAAATCCCCG | 65 |  |  |  |  |  |  |  |
| Pr032286001 | BP-221 | (AG)_10_ | GAAAACTCGGCAGAGCCTCATGG | 64 | 255 | 255 | 255 | 255 | 255 | 255 | 4--4 |
|  |  |  | GGCATTGACATTTCTAGCGCTGCAC | 64 |  |  |  |  |  |  |  |
| Pr032286002 | BP-222 | (AG)_10_ | CTCTGTCACGGAGCACCACGATATG | 65 | 274 | 275 | 275 | 275 | 275 | 275 | 2--2 |
|  |  |  | CTCCCTGATCACTTGTGTCCTGAG | 64 |  |  |  |  |  |  |  |
| Pr032286003 | BP-223 | (AG)_10_ | GGTAGTTCCTAAGAGGAGGATG | 60 | 279 | 0 | 0 | 0 | 0 | 0 |  |
|  |  |  | CTATGTCGTTCTCTGTAGACAGCC | 62 |  |  |  |  |  |  |  |
| Pr032286004 | BP-224 | (AG)_10_ | GACCAAACCCACCTCGAGATCG | 64 | 259 | 260 | 260 | 260 | 260 | 260 | 4--4 |
|  |  |  | GTGCTGCTTAAACGGACGATCCG | 64 |  |  |  |  |  |  |  |
| Pr032286005 | BP-225 | (AG)_10_ | CAGGACGCAGCAGAAGATATGG | 62 | 219 | 220 | 220 | 220 | 220 | 220 | 1--2 |
|  |  |  | CTACGTTTTGGATTCGGCTTCC | 60 |  |  |  |  |  |  |  |
| Pr032286006 | BP-226 | (AG)_10_ | GAGCTCCCAAGCATAACCGATCCTG | 65 | 204 | 200 | 200 | 200 | 200 | 205 | 4--9 |
|  |  |  | CCCTACACACCATACTCTCCCTCTC | 65 |  |  |  |  |  |  |  |
| Pr032286007 | BP-227 | (AG)_10_ | CACCCCTTGGCTTCATAGTGGTGAG | 65 | 232 | 235 | 235 | 235 | 235 | 235 | 6--6 |
|  |  |  | GCTTTAGGGGTTGCTAGATGGTAGG | 64 |  |  |  |  |  |  |  |
| Pr032286008 | BP-228 | (AG)_10_ | GCCTATGTGATTCGGAATGCGC | 62 | 164 | 0 | 0 | 0 | 0 | 0 |  |
|  |  |  | GCGCAGTAGATAATGAGCTCTCC | 62 |  |  |  |  |  |  |  |
| Pr032286009 | BP-229 | (AG)_10_ | GTTGAAGTTCGGGCAGACATAC | 60 | 259 | 250 | 225 | 250 | 225 | 250 | 5--8 |
|  |  |  | GACACAGCTGCCAAGCCTTATG | 62 |  |  |  |  |  |  |  |
| Pr032286010 | BP-230 | (AG)_10_ | GTTCAAAAGGAAGGGGTGGGAACG | 64 | 341 | 0 | 0 | 0 | 0 | 0 |  |
|  |  |  | GGATATGTGCTTGTGAGTGGGCTC | 64 |  |  |  |  |  |  |  |
| Pr032286011 | BP-231 | (AG)_10_ | GCTACCTTCCCTCCATGCGTAAG | 64 | 249 | 250 | 250 | 250 | 250 | 250 | 6--6 |
|  |  |  | CATACACGCACCGACACTGTGATG | 64 |  |  |  |  |  |  |  |
| Pr032286012 | BP-232 | (AG)_10_ | GCACAACGCCTCCTTCACATATC | 62 | 232 | 235 | 235 | 235 | 235 | 235 | 5--5 |
|  |  |  | GGTTGGAGGTACAACTACCTTGC | 62 |  |  |  |  |  |  |  |
| Pr032286013 | BP-233 | (AG)_10_ | GAGAGAGAGGCGGCTCATGAATG | 64 | 185 | 200 | 200 | 185 | 200 | 200 | 2--9 |
|  |  |  | GTGACCGGATGAGTTTTCAGTG | 60 |  |  |  |  |  |  |  |
| Pr032286014 | BP-234 | (AG)_10_ | GATTACACCGTGCATTCCTTCGCTG | 64 | 170 | 0 | 0 | 0 | 0 | 0 |  |
|  |  |  | CCCCACAGCGATGTCAATTTTCAC | 62 |  |  |  |  |  |  |  |
| Pr032286015 | BP-235 | (AG)_10_ | GAGAGAAAGTGTGGTGACCGTG | 62 | 197 | 200 | 205 | 200 | 205 | 200 | 3--6 |
|  |  |  | CCATAACTACTAACACCCACCG | 60 |  |  |  |  |  |  |  |
| Pr032286016 | BP-236 | (AG)_10_ | GTCTTTCCCGGCGGAACACAGG | 66 | 261 | 260 | 260 | 260 | 260 | 260 | 2--5 |
|  |  |  | CTGTCGGCTCTGGGATACGACGC | 67 |  |  |  |  |  |  |  |
| Pr032286017 | BP-237 | (AG)_10_ | GAGAGCTAACGCACAGTCGGAGAG | 65 | 171 | 170 | 175 | 175 | 175 | 175 | 3--8 |
|  |  |  | GGGTGGAAGGTGGGAAGAGGAATAC | 65 |  |  |  |  |  |  |  |
| Pr032286018 | BP-238 | (AG)_10_ | CACAGCACAACGTCAAAGTGTC | 60 | 275 | 275 | 275 | 275 | 275 | 275 | 3--3 |
|  |  |  | CATGTCTTCAGCAGTGTTGGAG | 60 |  |  |  |  |  |  |  |
| Pr032286019 | BP-239 | (AG)_10_ | GCTTTCTTATCCTCCTCCGCCAC | 64 | 198 | 200 | 200 | 200 | 200 | 200 | 5--5 |
|  |  |  | CCTTTCCGATCGTCTCTCAGCC | 64 |  |  |  |  |  |  |  |
| Pr032286020 | BP-240 | (AG)_10_ | GCTTCAACGCAGAACCAACTCG | 62 | 263 | 0 | 0 | 0 | 0 | 0 |  |
|  |  |  | GTTGGATCCAGACTGATGGCTTG | 62 |  |  |  |  |  |  |  |
| Pr032286021 | BP-241 | (AG)_10_ | CTACCTTTGCCGGTGGGAGGAG | 66 | 214 | 215 | 215 | 215 | 215 | 215 | 1--3 |
|  |  |  | CGCTAGTCACAGCCAGGTCTGAC | 66 |  |  |  |  |  |  |  |
| Pr032286022 | BP-242 | (AG)_10_ | GGAATAGCGGGAGGAAAAGGAAG | 62 | 286 | 285 | 285 | 285 | 285 | 285 | 2--2 |
|  |  |  | CTCCCTCTACTTCCTACCCTATTC | 62 |  |  |  |  |  |  |  |
| Pr032286023 | BP-243 | (AG)_10_ | GCGACCCTAATGTGGCCACTAAAG | 64 | 336 | 0 | 0 | 0 | 0 | 0 |  |
|  |  |  | CCCCTACGACACCTAAACCAAATCC | 64 |  |  |  |  |  |  |  |
| Pr032286024 | BP-244 | (AG)_10_ | GCCTAACAGTGTGGGTATGAAGC | 62 | 184 | 190 | 185 | 190 | 185 | 190 | 3--5 |
|  |  |  | GACAAAGCACTCCACCATAACC | 60 |  |  |  |  |  |  |  |
| Pr032286025 | BP-245 | (AG)_10_ | CTTAAGTGCACAGTTGCACGCAC | 62 | 208 | 205 | 205 | 200 | 205 | 205 | 3--8 |
|  |  |  | CGATTCCTTTCTCTCTCCCTCC | 62 |  |  |  |  |  |  |  |
| Pr032286026 | BP-246 | (AG)_10_ | CAGACAGAGAGAATGAGACGGTG | 62 | 128 | 0 | 0 | 0 | 0 | 0 |  |
|  |  |  | CCTACCGGCTACCACTGTCTCTC | 66 |  |  |  |  |  |  |  |
| Pr032286027 | BP-247 | (AG)_10_ | CCACATATAGCAGCCGGTCAAAG | 62 | 251 | 250 | 250 | 250 | 250 | 250 | 6--6 |
|  |  |  | CTGCTACAAACCGACCAGACAAG | 62 |  |  |  |  |  |  |  |
| Pr032286028 | BP-248 | (AG)_10_ | GAACGCCATGCATATTTCGAGG | 60 | 203 | 205 | 205 | 205 | 205 | 205 | 4--9 |
|  |  |  | GTGGGGATGTAACATCTACGTG | 60 |  |  |  |  |  |  |  |
| Pr032286029 | BP-249 | (AG)_10_ | GATAATAGGTTAGAGCTCGAGGGG | 62 | 274 | 275 | 275 | 275 | 275 | 275 | 3--4 |
|  |  |  | GTGAACATGTTCTACTTCGGCGG | 62 |  |  |  |  |  |  |  |
| Pr032286030 | BP-250 | (AG)_10_ | GCAGAGAAGGAGAATTGAGGTC | 60 | 182 | 185 | 185 | 185 | 185 | 185 | 8--8 |
|  |  |  | GTCCTTGAGAGTCATCGTGTTC | 60 |  |  |  |  |  |  |  |
| Pr032286031 | BP-251 | (AG)_10_ | GTAAATGACACCACTGTGGGCATG | 63 | 116 | 115 | 115 | 115 | 115 | 115 | 4--6 |
|  |  |  | CATTGGGTTGTCCTGAGTACCTC | 62 |  |  |  |  |  |  |  |
| Pr032286032 | BP-252 | (AG)_10_ | GCCAATCTAAGGAGGAAATCCC | 60 | 339 | 0 | 0 | 0 | 0 | 0 |  |
|  |  |  | CACTGTAAGCCCTTGAACCAAC | 60 |  |  |  |  |  |  |  |
| Pr032286033 | BP-253 | (AG)_10_ | GTCTTTCCCTTTAAGGCGGAGC | 62 | 189 | 190 | 190 | 190 | 190 | 190 | 4--9 |
|  |  |  | GAGCCTGAATCGCTAACGAACC | 62 |  |  |  |  |  |  |  |
| Pr032286034 | BP-254 | (AG)_10_ | GTACTTCACAGGCCAAGAGAGAG | 62 | 300 | 300 | 300 | 300 | 300 | 300 | 3--5 |
|  |  |  | GATCGATGTTACTAGACAGGCCC | 62 |  |  |  |  |  |  |  |
| Pr032286035 | BP-255 | (AG)_10_ | GAATTACAGGCGGTGCATCGTG | 62 | 288 | 0 | 0 | 0 | 0 | 0 |  |
|  |  |  | GAGGATGGTGCGAAGTTTCTTCG | 62 |  |  |  |  |  |  |  |
| Pr032286036 | BP-256 | (AG)_10_ | CCTATATGCACTAACCCTCCCC | 62 | 341 | 0 | 0 | 0 | 0 | 0 |  |
|  |  |  | CTGCTCACGTGGCCATAAAATCC | 62 |  |  |  |  |  |  |  |
| Pr032286037 | BP-257 | (AG)_10_ | GGTGGAAATTGCAGGGGTTTTG | 60 | 200 | 200 | 200 | 200 | 200 | 200 | 3--8 |
|  |  |  | CGCATGCATGCATGCATTAGTG | 60 |  |  |  |  |  |  |  |
| Pr032286038 | BP-258 | (AG)_10_ | GGTAGTAGCGTCAGTGTGAGAATG | 62 | 246 | 250 | 240 | 240 | 240 | 240 | 2--7 |
|  |  |  | CTCATCTGCCTCCTTCACTGCTTC | 64 |  |  |  |  |  |  |  |
| Pr032286039 | BP-259 | (AG)_10_ | GAGAGTGGGGACGTACATCAATAG | 62 | 248 | 250 | 255 | 255 | 255 | 255 | 3--7 |
|  |  |  | CAGACCCGAAATCCCGAAACTATC | 62 |  |  |  |  |  |  |  |
| Pr032286040 | BP-260 | (AG)_10_ | CACAGGAGAAACGCATAGATGGG | 62 | 214 | 215 | 215 | 215 | 215 | 215 | 3--7 |
|  |  |  | GTGTCCTCACCGACTTTGTTGGTG | 64 |  |  |  |  |  |  |  |
| Pr032286041 | BP-261 | (AG)_10_ | CCAGTAGACCAGTGGCCCATATAG | 64 | 199 | 200 | 200 | 200 | 200 | 200 | 5--5 |
|  |  |  | GTAGTTGTAGGCCTGCTGTGCATG | 64 |  |  |  |  |  |  |  |
| Pr032286042 | BP-262 | (AG)_10_ | CTGCACTGAGAGGGCATACTAAAC | 62 | 282 | 0 | 0 | 0 | 0 | 0 |  |
|  |  |  | CAACTTAGCTCGAGCATGTGGTTG | 62 |  |  |  |  |  |  |  |
| Pr032286043 | BP-263 | (AG)_10_ | GATTGAAGAACTTGGTGCAGCTCC | 62 | 224 | 225 | 225 | 225 | 225 | 225 | 3--3 |
|  |  |  | GACAGATGAACGAGCATCTCAAGG | 62 |  |  |  |  |  |  |  |
| Pr032286044 | BP-264 | (AG)_10_ | GTGATGGAGGCTAGTGGTTGTTTG | 62 | 178 | 175 | 175 | 175 | 175 | 175 | 2--6 |
|  |  |  | CCCAAGTTCCAACTCTTAGCTCTC | 62 |  |  |  |  |  |  |  |
| Pr032286045 | BP-265 | (AG)_10_ | GGCTTCAACTTCGTCACATTTCCC | 62 | 167 | 0 | 0 | 0 | 0 | 0 |  |
|  |  |  | CAATCGTCCATGGCAACTCTTCTC | 62 |  |  |  |  |  |  |  |
| Pr032286046 | BP-266 | (AG)_10_ | GAGTCCAGCTTTTGCTCTTTTGCC | 62 | 248 | 245 | 245 | 245 | 245 | 245 | 1--5 |
|  |  |  | GTCACAGCCTCGCAGTCTTAATAC | 62 |  |  |  |  |  |  |  |
| Pr032286047 | BP-267 | (AG)_10_ | CATACAGTATCTGTCGTCTTGGGC | 62 | 328 | 0 | 0 | 0 | 0 | 0 |  |
|  |  |  | GCCTCCTAGAAACTGTGGTTGTG | 62 |  |  |  |  |  |  |  |
| Pr032286048 | BP-268 | (AG)_10_ | GTCAAGCTCAAGAGATCCCTTG | 60 | 304 | 305 | 310 | 310 | 305 | 305 | 4--8 |
|  |  |  | GTTTCTGTCGGCAAGGAAAAGG | 60 |  |  |  |  |  |  |  |
| Pr032286049 | BP-269 | (AG)_10_ | GAGAGGTCTTTGGGTCAAGGAAG | 62 | 244 | 250 | 250 | 250 | 250 | 250 | 3--7 |
|  |  |  | GTTCCTCGGCTATGAACCAAAGC | 62 |  |  |  |  |  |  |  |
| Pr032286050 | BP-270 | (AG)_10_ | CACAGAGAGAAAGAGAACGCCAG | 62 | 170 | 0 | 0 | 0 | 0 | 0 |  |
|  |  |  | CCAAACCCCACCCCAAAATATTCC | 62 |  |  |  |  |  |  |  |
| Pr032286051 | BP-271 | (AG)_10_ | GGTTGAGATCGGACCTATTTCG | 60 | 148 | 150 | 150 | 150 | 150 | 150 | 2--2 |
|  |  |  | GGCGTGTAGAAAGTTGGAAGTC | 60 |  |  |  |  |  |  |  |
| Pr032286052 | BP-272 | (AG)_10_ | GGAAAGCAAGTACACTGGCATG | 60 | 243 | 245 | 245 | 245 | 245 | 245 | 6--6 |
|  |  |  | CTTTCTTCTGTGGAATCCGTGC | 60 |  |  |  |  |  |  |  |
| Pr032286053 | BP-273 | (AG)_10_ | GAATCAGTTCTCTCGGCCAAAAGC | 62 | 296 | 300 | 300 | 300 | 300 | 300 | 5--5 |
|  |  |  | GCCCAGTTAACTCAGGGTAAATCC | 62 |  |  |  |  |  |  |  |
| Pr032286054 | BP-274 | (AG)_10_ | GGGCTCATAAAGATCTACAGGC | 60 | 266 | 250 | 250 | 250 | 250 | 250 | 3--5 |
|  |  |  | CAATACATCATTTGGTGGGGCC | 60 |  |  |  |  |  |  |  |
| Pr032286055 | BP-275 | (AG)_10_ | CAATGATGAAGCCCTAGCGACC | 62 | 162 | 165 | 165 | 165 | 165 | 165 | 2--5 |
|  |  |  | GTCAGGGGTGGGAGTTTACTTAC | 62 |  |  |  |  |  |  |  |
| Pr032286056 | BP-276 | (AG)_10_ | CATTAATGGGTTTGGGCAGGCAC | 62 | 211 | 220 | 220 | 220 | 215 | 220 | 3--5 |
|  |  |  | CTAAGGAGGCATCTTATGGGTCC | 62 |  |  |  |  |  |  |  |
| Pr032286057 | BP-277 | (AG)_10_ | GGTGCCTGATGATCAATTGTCCC | 62 | 370 | 370 | 370 | 370 | 370 | 370 | 1--4 |
|  |  |  | GATTTCCAACAGCCCAAAGGGAC | 64 |  |  |  |  |  |  |  |
| Pr032286058 | BP-278 | (AG)_10_ | GTGTTTTGGAGGTTGGAGACAGG | 62 | 187 | 185 | 185 | 185 | 185 | 185 | 4--4 |
|  |  |  | CTGCAACCAGGCAACACGATAAG | 62 |  |  |  |  |  |  |  |
| Pr032286059 | BP-279 | (AG)_10_ | GTCGGTGTAGGGCGACTGAGATATG | 65 | 126 | 125 | 125 | 125 | 125 | 125 | 3--8 |
|  |  |  | CCTCTCCCCCATTTCGTCTGAAACC | 65 |  |  |  |  |  |  |  |
| Pr032286060 | BP-280 | (AG)_10_ | GTACACCGGACTCGCAGGCCTC | 68 | 302 | 300 | 300 | 300 | 300 | 300 | 3--3 |
|  |  |  | GATCCAGCGATGACTGGTGGGCC | 67 |  |  |  |  |  |  |  |
| Pr032286061 | BP-281 | (AG)_10_ | GGCTTCGACGACATAGTTGGATCC | 64 | 236 | 235 | 235 | 235 | 235 | 235 | 5--5 |
|  |  |  | GAACAGACAGTTGCGTGCTCAGAG | 64 |  |  |  |  |  |  |  |
| Pr032286062 | BP-282 | (AG)_10_ | CATTCCGCGTACTAAACGAGTTC | 60 | 251 | 250 | 250 | 260 | 250 | 250 | 2--5 |
|  |  |  | CATACGGAATATGAGCAACGGCG | 62 |  |  |  |  |  |  |  |
| Pr032286063 | BP-283 | (AG)_10_ | GTTCCCATGGGCTGTAGCAGTTC | 64 | 183 | 185 | 185 | 185 | 185 | 185 | 3--3 |
|  |  |  | GAAGGACCATCTGAGAGCTAGGG | 64 |  |  |  |  |  |  |  |
| Pr032286064 | BP-284 | (GA)_10_ | GGTAAAACCGCCTTAACTGGCAGCC | 65 | 282 | 0 | 0 | 0 | 0 | 0 |  |
|  |  |  | GGACCTGGTTTGGTTAAGGTGGG | 64 |  |  |  |  |  |  |  |
| Pr032286065 | BP-285 | (GA)_10_ | GGTGATGTTGGTTGGCAATGCCGG | 65 | 199 | 200 | 200 | 200 | 200 | 200 | 2--2 |
|  |  |  | CGTTTCAAACCCATACAGGCGACGC | 65 |  |  |  |  |  |  |  |
| Pr032286066 | BP-286 | (GA)_10_ | GCCTTAGCCACCCTACCTCTCCAG | 67 | 185 | 185 | 185 | 185 | 185 | 185 | 3--9 |
|  |  |  | CCAGACCAGATCACTGGCCACAGCC | 69 |  |  |  |  |  |  |  |
| Pr032286067 | BP-287 | (GA)_10_ | CAAACCCAACTAATCCTCGCG | 60 | 275 | 300 | 300 | 300 | 275 | 300 | 3--7 |
|  |  |  | CGCGTACTGGTTTGAATCCAGC | 62 |  |  |  |  |  |  |  |
| Pr032286068 | BP-288 | (GA)_10_ | GAATGTGCCCTTCACCAACCTC | 62 | 253 | 250 | 250 | 250 | 250 | 250 | 6--6 |
|  |  |  | CTTCTCCACAAGTAGGGTTCCG | 62 |  |  |  |  |  |  |  |
| Pr032286069 | BP-289 | (GA)_10_ | GTTAGGGCAGGAGAAATGTCGAG | 61 | 305 | 300 | 300 | 300 | 300 | 300 | 3--3 |
|  |  |  | CAAGCTCCGAATTCCACTTGGATCG | 64 |  |  |  |  |  |  |  |
| Pr032286070 | BP-290 | (GA)_10_ | CCCAAACGATATCCTCTCCCTG | 62 | 267 | 0 | 0 | 0 | 0 | 0 |  |
|  |  |  | CAAGGAGAAAACCTCACCAGGG | 62 |  |  |  |  |  |  |  |
| Pr032286071 | BP-291 | (GA)_10_ | GCACTCTAAGGACACAAGTCTGC | 62 | 294 | 300 | 300 | 300 | 300 | 300 | 4--4 |
|  |  |  | CTTTTATTCCCCAGCTCACAGCC | 62 |  |  |  |  |  |  |  |
| Pr032286072 | BP-292 | (GA)_10_ | GCGTTTACACAGAGAGAGAGAG | 60 | 219 | 220 | 230 | 230 | 230 | 230 | 2--6 |
|  |  |  | CTTCTGTCTCTCACAGGTACACG | 62 |  |  |  |  |  |  |  |
| Pr032286073 | BP-293 | (GA)_10_ | GCGAGAGGGAAAGTACACGAAAG | 62 | 174 | 175 | 180 | 175 | 180 | 180 | 4--7 |
|  |  |  | GGTAGATCCCAAAGGTCTCTCTC | 62 |  |  |  |  |  |  |  |
| Pr032286074 | BP-294 | (GA)_10_ | GTGTACTAGGGTTGCGTCCCTCTG | 65 | 219 | 0 | 0 | 0 | 0 | 0 |  |
|  |  |  | CAGCCTCTCTCTCCCTTCTGCTTC | 65 |  |  |  |  |  |  |  |
| Pr032286075 | BP-295 | (GA)_10_ | GTTCACTGGTCATCGCCAACGTAC | 64 | 241 | 0 | 0 | 0 | 0 | 0 |  |
|  |  |  | GATGTGAATGGTCCTTCCACAC | 60 |  |  |  |  |  |  |  |
| Pr032286076 | BP-296 | (GA)_10_ | GAGAGAGATTGCAGGGGGGAGAAG | 65 | 205 | 205 | 205 | 215 | 205 | 215 | 5--8 |
|  |  |  | CCACTTCCCCCCATTTTCCCATCTC | 65 |  |  |  |  |  |  |  |
| Pr032286077 | BP-297 | (GA)_10_ | GAAATGCGAACTTCCCCCCAGATG | 64 | 183 | 0 | 0 | 0 | 0 | 0 |  |
|  |  |  | CAGGCAGCTGAGAATAGGGTTGG | 64 |  |  |  |  |  |  |  |
| Pr032286078 | BP-298 | (GA)_10_ | CTATGGCGCACTCAAATCCTCATC | 62 | 239 | 240 | 240 | 245 | 240 | 240 | 4--6 |
|  |  |  | CACTTTGTGTGAAAGGCGCTTGG | 62 |  |  |  |  |  |  |  |
| Pr032286079 | BP-299 | (GA)_10_ | CACCACCGAATGCCGTCGAAATCTC | 65 | 154 | 160 | 160 | 155 | 160 | 155 | 6--8 |
|  |  |  | GTGGCGTATTCCGGCGGTAGGTTTC | 67 |  |  |  |  |  |  |  |
| Pr032286080 | BP-300 | (GA)_10_ | CAGCTCAAGGACACAGCAACCAG | 64 | 367 | 367 | 367 | 375 | 367 | 367 | 3--5 |
|  |  |  | CAAGGGGGTGTTTCACAGCCGATC | 65 |  |  |  |  |  |  |  |
| Pr032286081 | BP-301 | (GA)_10_ | GCGGGAACGGTTATCAGAATTCG | 62 | 146 | 150 | 135 | 135 | 135 | 150 | 2--2 |
|  |  |  | GGATTTCGCCTTCTTTGAACCGC | 62 |  |  |  |  |  |  |  |
| Pr032286082 | BP-302 | (GA)_10_ | CACACCTCGTAGGAACTTGTGTG | 62 | 244 | 0 | 0 | 0 | 0 | 0 |  |
|  |  |  | CGGTCACTGAGTCCATTTCACATTC | 62 |  |  |  |  |  |  |  |
| Pr032286083 | BP-303 | (GA)_10_ | GCAACTGGATTATTACCGACGC | 60 | 240 | 0 | 0 | 0 | 0 | 0 |  |
|  |  |  | GAGCAATTTGCTCTACTGGAGC | 60 |  |  |  |  |  |  |  |
| Pr032286084 | BP-304 | (GA)_10_ | GCTCTAACGAAACCCGCCGAAAG | 64 | 188 | 190 | 190 | 200 | 190 | 190 | 3--5 |
|  |  |  | CTCCACCTCATCTTACCATTGGC | 62 |  |  |  |  |  |  |  |
| Pr032286085 | BP-305 | (GA)_10_ | GCTGGCTTTGTGAACCCATGTG | 62 | 227 | 225 | 230 | 225 | 225 | 225 | 4--6 |
|  |  |  | GAGGTCTTGGCGCTCCAGAAAC | 64 |  |  |  |  |  |  |  |
| Pr032286086 | BP-306 | (GA)_10_ | CATGCTCCAAGAACACACCTTG | 60 | 248 | 250 | 250 | 250 | 245 | 250 | 3--5 |
|  |  |  | CTGAACTAGACTCCGGGTTTCTC | 62 |  |  |  |  |  |  |  |
| Pr032286087 | BP-307 | (GA)_10_ | CTGGAAACACCAGATCGGTGAG | 62 | 224 | 250 | 250 | 250 | 250 | 250 | 1--5 |
|  |  |  | CACGCATTGCTGGGGCTTCATTG | 64 |  |  |  |  |  |  |  |
| Pr032286088 | BP-308 | (GA)_10_ | GACTCTCCCTCTAGGGTTTGTG | 62 | 200 | 0 | 0 | 0 | 0 | 0 |  |
|  |  |  | CAACAGCCACAGGCTCACACAG | 64 |  |  |  |  |  |  |  |
| Pr032286089 | BP-309 | (GA)_10_ | GCTTGGCAGATGACACTTGAAG | 60 | 185 | 185 | 190 | 190 | 190 | 185 | 4--9 |
|  |  |  | GTGCAGACACTTGCATGGGAATG | 62 |  |  |  |  |  |  |  |
| Pr032286090 | BP-310 | (GA)_10_ | GGGCGGCCCTTAACTAGGTTTAAG | 64 | 220 | 0 | 0 | 0 | 0 | 0 |  |
|  |  |  | CGAAATGCGGATACCGAGGAGAAG | 64 |  |  |  |  |  |  |  |
| Pr032286091 | BP-311 | (GA)_10_ | GCGAAGAAGATAGCAAGAACCG | 60 | 220 | 220 | 220 | 230 | 230 | 230 | 3--8 |
|  |  |  | GAACCCCTGAAAGCTCTGTGTTG | 62 |  |  |  |  |  |  |  |
| Pr032286092 | BP-312 | (GA)_10_ | GATCAACCCGTCCTACGTACATC | 62 | 368 | 0 | 0 | 0 | 0 | 0 |  |
|  |  |  | GGTTGCCTCTAGCGCTTTTACC | 62 |  |  |  |  |  |  |  |
| Pr032286093 | BP-313 | (GA)_10_ | GAAGGTTGAAACCTTCAGCACC | 60 | 208 | 210 | 210 | 210 | 210 | 210 | 3--4 |
|  |  |  | CCGGATATGGAAAGAACAGCAG | 60 |  |  |  |  |  |  |  |
| Pr032286094 | BP-314 | (GA)_10_ | GATGGTGTTCGTGGCCATAGAGG | 64 | 192 | 0 | 0 | 0 | 0 | 0 |  |
|  |  |  | CGTCGTTGAAAGCACCAGCGATG | 64 |  |  |  |  |  |  |  |
| Pr032286095 | BP-315 | (GA)_10_ | GAAGAATCGGGTTTGGACAACG | 60 | 422 | 425 | 425 | 425 | 425 | 425 | 1--4 |
|  |  |  | CGATCACAAAACCAATCTCCCC | 60 |  |  |  |  |  |  |  |
| Pr032286096 | BP-316 | (GA)_10_ | CAGGTTGGGAGAATAGATCGGAG | 62 | 192 | 200 | 200 | 200 | 190 | 200 | 4--7 |
|  |  |  | CTCTACATGCCACGTGTTCTCTC | 62 |  |  |  |  |  |  |  |
| Pr032286097 | BP-317 | (GA)_10_ | GATTTACGGTCTTCTTAGCGGCC | 62 | 163 | 0 | 0 | 0 | 0 | 0 |  |
|  |  |  | CTCTCATCCAAAGCACCCAACC | 62 |  |  |  |  |  |  |  |
| Pr032286098 | BP-318 | (GA)_10_ | CTTTCCACGAAACAGACACAGG | 60 | 173 | 175 | 175 | 175 | 175 | 175 | 3--6 |
|  |  |  | CACTCTCACGCAAGACACACAAC | 62 |  |  |  |  |  |  |  |
| Pr032286099 | BP-319 | (GA)_10_ | GAAAGAAAGCACAGAGGAGCAC | 60 | 195 | 200 | 200 | 200 | 200 | 200 | 4--7 |
|  |  |  | GGCTGCCAACAAACAGTACTAC | 60 |  |  |  |  |  |  |  |
| Pr032286100 | BP-320 | (GA)_10_ | GGTGTTGGGTCTCATGCAAATC | 60 | 168 | 170 | 170 | 170 | 175 | 175 | 4--7 |
|  |  |  | CCCTACCAGATCTTCAAATGGC | 60 |  |  |  |  |  |  |  |
| Pr032286101 | BP-321 | (GA)_10_ | GGTTGGGTAATTGCATCACTGGTG | 62 | 288 | 300 | 300 | 300 | 300 | 300 | 5--5 |
|  |  |  | CTCAGCCATGCTGGATATCATCTG | 62 |  |  |  |  |  |  |  |
| Pr032286102 | BP-322 | (GA)_10_ | GCTTACCAGCTAAGGGAGAATCAG | 62 | 262 | 0 | 0 | 0 | 0 | 0 |  |
|  |  |  | GACCCGCAGATGGGAAGTTATTTG | 62 |  |  |  |  |  |  |  |
| Pr032286103 | BP-323 | (GA)_10_ | GGGGATTACAATCTCCCTCTTTGG | 62 | 350 | 350 | 350 | 350 | 350 | 350 | 4--4 |
|  |  |  | CTTCCTCTCTCTTGTCATCTCCAC | 62 |  |  |  |  |  |  |  |
| Pr032286104 | BP-324 | (GA)_10_ | GCTATTTGGGTCACTTTGCCAG | 60 | 243 | 245 | 245 | 245 | 245 | 245 | 2--2 |
|  |  |  | GGGGACATGTATCACTTGCTTG | 60 |  |  |  |  |  |  |  |
| Pr032286105 | BP-325 | (GA)_10_ | GAGGAGGATGTAAGCGAGGTAG | 62 | 181 | 180 | 180 | 180 | 180 | 180 | 3--7 |
|  |  |  | CTGGACGATGAGAAGGACAACC | 62 |  |  |  |  |  |  |  |
| Pr032286106 | BP-326 | (GA)_10_ | CAAAAGTCCGGAAGATGTTGGGG | 62 | 249 | 250 | 250 | 250 | 250 | 250 | 3--3 |
|  |  |  | GATGGGGGCAGAAGGATATTGTG | 62 |  |  |  |  |  |  |  |
| Pr032286107 | BP-327 | (GA)_10_ | CGCTACCTCTCCTGCAAAGTATAG | 62 | 298 | 300 | 300 | 300 | 300 | 300 | 2--2 |
|  |  |  | GTACTTTCACAGTGACAGGGAAGG | 62 |  |  |  |  |  |  |  |
| Pr032286108 | BP-328 | (GA)_10_ | GCTCCTCTACATAGACCCGTAT | 60 | 138 | 0 | 0 | 0 | 0 | 0 |  |
|  |  |  | CTTTCAGAGGCGAAAGGCCATG | 62 |  |  |  |  |  |  |  |
| Pr032286109 | BP-329 | (GA)_10_ | CTGAAGACCCTCCGATGCTTAAG | 62 | 150 | 150 | 150 | 150 | 175 | 150 | 4--10 |
|  |  |  | CCATTTGACGAGGACTTCTGGAC | 62 |  |  |  |  |  |  |  |
| Pr032286110 | BP-330 | (GA)_10_ | CATAGAGGGAACCATACCAGCG | 62 | 227 | 225 | 225 | 225 | 225 | 225 | 7--7 |
|  |  |  | GACAGAGGTCAGCGTCACAATG | 62 |  |  |  |  |  |  |  |
| Pr032286111 | BP-331 | (GA)_10_ | CATATGTTTGAGGAGGATGGAG | 58 | 157 | 0 | 0 | 0 | 0 | 0 |  |
|  |  |  | CTGATTCTCCTTCGTCTCTCTC | 60 |  |  |  |  |  |  |  |
| Pr032286112 | BP-332 | (GA)_10_ | GCTGGGAATAATCCTCTGAAGCTG | 62 | 243 | 245 | 245 | 245 | 245 | 245 | 5--5 |
|  |  |  | GAGATAGGGTACCCTATCTCTCC | 62 |  |  |  |  |  |  |  |
| Pr032286113 | BP-333 | (GA)_10_ | GTTCCAGTTCCCAACTGGTCCAAC | 64 | 137 | 135 | 135 | 135 | 135 | 135 | 2--8 |
|  |  |  | GCATTTCATAGAGGTCGTGCACC | 62 |  |  |  |  |  |  |  |
| Pr032286114 | BP-334 | (GA)_10_ | CGGTTAAACCCAGTGGCATTGG | 62 | 191 | 190 | 190 | 190 | 190 | 190 | 5--5 |
|  |  |  | GACATCTATTGCCTTGCTCTGG | 60 |  |  |  |  |  |  |  |
| Pr032286115 | BP-335 | (GA)_10_ | GGTTGCATTCAGTCTCACAGACAG | 62 | 198 | 200 | 200 | 200 | 200 | 200 | 2--2 |
|  |  |  | GATGTTGAAGCTGAGGCTAGCTGTG | 64 |  |  |  |  |  |  |  |
| Pr032286116 | BP-336 | (GA)_10_ | GTGTTCTCTTCGCATCTCTTCACG | 62 | 292 | 300 | 300 | 300 | 300 | 300 | 6--6 |
|  |  |  | GTTTTCTCGTAGACGGTGTCAGC | 62 |  |  |  |  |  |  |  |
| Pr032286117 | BP-337 | (GA)_10_ | CCACAACGATGTAGGCATGAGAG | 62 | 187 | 185 | 185 | 185 | 185 | 185 | 4--6 |
|  |  |  | GTTTCCTTCCCATGCTGACTCTG | 62 |  |  |  |  |  |  |  |
| Pr032286118 | BP-338 | (GA)_10_ | CACTTGTGCCCGATAACTCAAG | 60 | 168 | 170 | 170 | 170 | 170 | 170 | 3--6 |
|  |  |  | GCCACGATTTGGTCGTTCAAAC | 60 |  |  |  |  |  |  |  |
| Pr032286119 | BP-339 | (GA)_10_ | GTTACACACCCACAAGCCACAGAC | 64 | 186 | 185 | 185 | 185 | 185 | 185 | 4--4 |
|  |  |  | CTGGCAGTATCTCTCTCTCTCTCC | 62 |  |  |  |  |  |  |  |
| Pr032286120 | BP-340 | (GA)_10_ | GTTGCGAAGAGCAGGTTCAAGGTC | 64 | 190 | 190 | 190 | 190 | 190 | 190 | 3--3 |
|  |  |  | CGTCTTCTTATCCGCCAATCTCTC | 62 |  |  |  |  |  |  |  |
| Pr032286121 | BP-341 | (GA)_10_ | GATGTGCATGCTCTTCGGCTGG | 64 | 344 | 0 | 0 | 0 | 0 | 0 |  |
|  |  |  | CAACAGCAGCGGCCGAAGATTG | 64 |  |  |  |  |  |  |  |
| Pr032286122 | BP-342 | (GA)_10_ | CGGAAACCTCCATTTGGACTCC | 62 | 287 | 285 | 285 | 285 | 285 | 285 | 7--7 |
|  |  |  | CCACTTGGAATGCACTGCATCC | 62 |  |  |  |  |  |  |  |
| Pr032286123 | BP-343 | (GA)_10_ | GGGGCCTGGAATAACATGCACG | 64 | 227 | 225 | 225 | 225 | 225 | 225 | 2--2 |
|  |  |  | CCACTTCGGCGAAGGATCCAATG | 64 |  |  |  |  |  |  |  |
| Pr032286124 | BP-344 | (GA)_10_ | GTGGATGCGGTTATTGGCCATATC | 62 | 161 | 160 | 160 | 160 | 160 | 160 | 3--7 |
|  |  |  | GATATGGCCAATAACCGCATCCAC | 62 |  |  |  |  |  |  |  |
| Pr032286125 | BP-345 | (CT)_9_ | CTATATTGGCTCCAAGCAC | 55 | 105 | 100 | 100 | 100 | 100 | 100 | 3--3 |
|  |  |  | ACACCCACACTGACAGATAA | 56 |  |  |  |  |  |  |  |
| Pr032286126 | BP-346 | (TC)_26_ | GAAAGCATGAGACCCGTCTT | 58 | 172 | 165 | 165 | 165 | 165 | 165 | 4--6 |
|  |  |  | AACCTAAACAGCCTGCCAAA | 56 |  |  |  |  |  |  |  |
| Pr032286127 | BP-347 | (GT)_10_ | GAAAGGATCTGTATAGCCAAC | 56 | 160 | 0 | 0 | 0 | 0 | 0 |  |
|  |  |  | ACCACATGGCAGCAATTCTAG | 56 |  |  |  |  |  |  |  |
| Pr032286128 | BP-348 | (GT)_6_ | CTGATTCCTGAGAATGTGAAG | 54 | 122 | 0 | 0 | 0 | 0 | 0 |  |
|  |  |  | AGCACTACTCAAGTACACAAG | 54 |  |  |  |  |  |  |  |
| Pr032286129 | BP-349 | (GT)_28_ | GTTGTAATGCAAACACATGGG | 54 | 167 | 165 | 165 | 165 | 165 | 165 | 3--3 |
|  |  |  | TCTGTGTCATAATTGGGTAGG | 54 |  |  |  |  |  |  |  |
| Pr032286130 | BP-350 | (GT)_8_...(AT)_4_ | GGTATTCAGACTGCTCATACC | 56 | 114 | 0 | 0 | 0 | 0 | 0 |  |
|  |  |  | TATCCTATGCAAGGGACGAAG | 56 |  |  |  |  |  |  |  |
| Pr032286131 | BP-351 | (GT)_9_ | GCCTGCTTTCCATTCGTACAC | 56 | 151 | 150 | 150 | 150 | 150 | 150 | 6--6 |
|  |  |  | TCCCGGTTAAGTCAAAGTTCC | 56 |  |  |  |  |  |  |  |
| Pr032286132 | BP-352 | (GT)_11_C(AG)_5_ | TTAGCAGTTACTTACTTTACTGTATCC | 56 | 165 | 165 | 165 | 165 | 165 | 165 | 4--4 |
|  |  |  | TTGGTCGTGGGTACTGTATTG | 56 |  |  |  |  |  |  |  |
| Pr032286133 | BP-353 | (GT)_3_GC(GT)_5_G_12_ | GTCTTTAAACCAAGGCTACGC | 56 | 134 | 0 | 0 | 0 | 0 | 0 |  |
|  |  |  | ATCAACTGTGTCATGCCCATG | 56 |  |  |  |  |  |  |  |
| Pr032286134 | BP-354 | T_9_(GT)_13_ | CCATTTTCCCTTGAGTAGGCC | 56 | 105 | 100 | 100 | 100 | 100 | 100 | 2--2 |
|  |  |  | GATCAATGGCAATTGTACCAC | 56 |  |  |  |  |  |  |  |
| Pr032286135 | BP-355 | (GT)_9_ | GCAATCACATTCAAACCCCATAGCCAC | 56 | 169 | 0 | 0 | 0 | 0 | 0 |  |
|  |  |  | CAATGAACCGTAGGAGAA CTG | 56 |  |  |  |  |  |  |  |
| Pr032286136 | BP-356 | (AT)_6_ | CGGCGTATTCTGGTGTTATTC | 56 | 141 | 0 | 0 | 0 | 0 | 0 |  |
|  |  |  | TGACCAATGAAGCAATGCGTG | 56 |  |  |  |  |  |  |  |
| Pr032286137 | BP-357 | (CT)_5_ | GCCGGGGAAGAAAGTTACC | 60 | 250 | 250 | 250 | 250 | 250 | 250 | 5--5 |
|  |  |  | CACGTTGGGAATGTGATGAT | 56 |  |  |  |  |  |  |  |
| Pr032286138 | BP-358 | (CT)_9_ | CCAACAGGCTTTCATTTGCT | 56 | 213 | 215 | 215 | 215 | 215 | 215 | 1--1 |
|  |  |  | ATCAGGGGCATCAACAAGAG | 58 |  |  |  |  |  |  |  |
| Pr032286139 | BP-359 | (CTT)_5_ | TCTCTTCCCGCAAACTCTCT | 58 | 232 | 230 | 230 | 230 | 230 | 230 | 3--3 |
|  |  |  | ATAAACCGCCCAGGAAAAAC | 56 |  |  |  |  |  |  |  |
| Pr032286140 | BP-360 | (ATC)_7_ | CCCCCTCCCTTTTACTCTTTC | 60 | 214 | 215 | 215 | 215 | 215 | 215 | 4--4 |
|  |  |  | TTCTGCTCCCGTCTCATCTT | 58 |  |  |  |  |  |  |  |
| Pr032286141 | BP-361 | (CT)_6_ | GCGACACACCCTACCATCTT | 60 | 219 | 220 | 220 | 220 | 220 | 220 | 2--2 |
|  |  |  | GGTGCACTTGCAGATGTGAT | 58 |  |  |  |  |  |  |  |
| Pr032286142 | BP-362 | (AAG)_6_ | AGGGTGTTCAAACCGACGA | 58 | 231 | 0 | 0 | 0 | 0 | 0 |  |
|  |  |  | CGGTCTCAATCTCCACGTTT | 58 |  |  |  |  |  |  |  |
| Pr032286143 | BP-363 | (AG)_8_ | GCACTCACTCGGATACTCGTC | 62 | 197 | 200 | 200 | 200 | 200 | 200 | 5--5 |
|  |  |  | CTTTTGCACCATGTTTGTGG | 56 |  |  |  |  |  |  |  |
| Pr032286144 | BP-364 | (AG)_11_ | TAGAGCGTTGCGCAGATAGA | 58 | 187 | 190 | 190 | 190 | 190 | 190 | 4--4 |
|  |  |  | CAGGTTCCTCTCCTCCACTG | 62 |  |  |  |  |  |  |  |
| Pr032286145 | BP-365 | (AG)_5_ | TTTATTTTTCAATTTTTCCTAGAGAGG | 54 | 250 | 250 | 250 | 250 | 250 | 250 | 2--2 |
|  |  |  | ACCACACCGAGGCATACAAT | 58 |  |  |  |  |  |  |  |
| Pr032286146 | BP-366 | (CT)_11_ | GCGACAGGAAATTCAACCAC | 58 | 243 | 250 | 250 | 250 | 250 | 250 | 4--4 |
|  |  |  | CTGCGTCAGACTGCACATTT | 58 |  |  |  |  |  |  |  |
| Pr032286147 | BP-367 | (AG)_6_ | TGAATAGACCGTTGCGCTTA | 56 | 110 | 110 | 110 | 110 | 110 | 110 | 3--3 |
|  |  |  | CGTATCTCTCGGCTTGCTCT | 60 |  |  |  |  |  |  |  |
| Pr032286148 | BP-368 | (CTT)_6_ | CGCCAAATCTTTACCCAGAA | 56 | 164 | 0 | 0 | 0 | 0 | 0 |  |
|  |  |  | GGACGATGATGATCCATGAG | 58 |  |  |  |  |  |  |  |
| Pr032286149 | BP-369 | (AG)_6_ | CGGGGGACATTACAAATAGC | 58 | 202 | 200 | 200 | 200 | 200 | 200 | 2--2 |
|  |  |  | TCGCATCTTCATCTGTGAGG | 58 |  |  |  |  |  |  |  |
| Pr032286150 | BP-370 | (AG)_7_ | CCCTGCCTCTTCTCTGTCAC | 62 | 233 | 0 | 0 | 0 | 0 | 0 |  |
|  |  |  | GCCATAAGCCTCCAATCTCA | 58 |  |  |  |  |  |  |  |
| Pr032286151 | BP-371 | (CAT)_5_ | GGACTTCTTCGGAGACATGG | 60 | 215 | 215 | 215 | 215 | 215 | 215 | 4--4 |
|  |  |  | CCCCAGAAAATAACGGCATA | 56 |  |  |  |  |  |  |  |
| Pr032286152 | BP-372 | (CTT)_10_ | CCTCTCTGGCTTCTCCTCCT | 62 | 180 | 0 | 0 | 0 | 0 | 0 |  |
|  |  |  | TCGAATCCATATCCACCAAAA | 54 |  |  |  |  |  |  |  |
| Pr032286153 | BP-373 | (CT)_8_ | CGGGGGAACCATCAAAAA | 55 | 187 | 0 | 0 | 0 | 0 | 0 |  |
|  |  |  | CGGGAAGTCGCATATAGGAA | 58 |  |  |  |  |  |  |  |
| Pr032286154 | BP-374 | (GAA)_5_ | GGCGTTTAATCTGGGTGAGA | 58 | 157 | 160 | 160 | 160 | 160 | 160 | 6--6 |
|  |  |  | ACGCCAGAATGGTAGACACC | 60 |  |  |  |  |  |  |  |
| Pr032286155 | BP-375 | (AG)_7_ | TCAGCTTCGTTCCAAAACCT | 56 | 210 | 210 | 210 | 210 | 210 | 210 | 3--3 |
|  |  |  | CCCATTTGGAGATGGAGAAA | 56 |  |  |  |  |  |  |  |
| Pr032286156 | BP-376 | (CT)_13_ | ACTGCAGCCCTTCAACTACG | 60 | 209 | 210 | 210 | 210 | 210 | 210 | 2--2 |
|  |  |  | AACGCCTACCTCATGTTTGG | 58 |  |  |  |  |  |  |  |
| Pr032286157 | BP-377 | (CT)_13_ | GTCAGGTAGTTAGGGGCATT | 58 | 168 | 165 | 165 | 165 | 165 | 165 | 2--2 |
|  |  |  | AAGCGGGTAAAAGGAGTGTG | 58 |  |  |  |  |  |  |  |
| Pr032286158 | BP-378 | (CT)_13_ | CCTCCTTTCTCTTTCCCACA | 58 | 202 | 0 | 0 | 0 | 0 | 0 |  |
|  |  |  | ACATCCCCCGCATCTCTTCT | 60 |  |  |  |  |  |  |  |
| Pr032286159 | BP-379 | (AC)_9_ | ACTCTCCACTAAATAATGCC | 54 | 112 | 0 | 0 | 0 | 0 | 0 |  |
|  |  |  | CGCCGATAACACTAAACCCT | 58 |  |  |  |  |  |  |  |
| Pr032286160 | BP-380 | (CT)_11_ | AAAAACCAACCAGCCAAGTG | 56 | 232 | 235 | 235 | 235 | 235 | 235 | 2--2 |
|  |  |  | CTGCACCTCCCAACAGTTCT | 60 |  |  |  |  |  |  |  |
| Pr032286161 | BP-381 | (CT)_14_ | GCAATTTCTACCAAACAGAACC | 56 | 185 | 185 | 185 | 185 | 185 | 185 | 4--4 |
|  |  |  | CTCTGGATCCAACGGTCAAG | 60 |  |  |  |  |  |  |  |
| Pr032286162 | BP-382 | (AG)_17_ | CTTACCGTCCTGCCAAGGT | 60 | 235 | 235 | 235 | 235 | 235 | 235 | 6--6 |
|  |  |  | ACCACCACAGCCACAACC | 60 |  |  |  |  |  |  |  |
| Pr032286163 | BP-383 | (CT)_11_ | GCGACAGGAAATTCAACCAC | 58 | 174 | 175 | 175 | 175 | 175 | 175 | 6--6 |
|  |  |  | CACCTTCCATTTTTCCCAGA | 56 |  |  |  |  |  |  |  |
| Pr032286164 | BP-384 | (ATG)_7_ | TTTTTGATGCACGGATGAGA | 54 | 174 | 175 | 175 | 175 | 175 | 175 | 2--5 |
|  |  |  | ACACGTGTCAACACGAAAGC | 58 |  |  |  |  |  |  |  |
| Pr032286165 | BP-385 | (AT)_14_ | TGGCAGTTCAATCATGTGGT | 56 | 205 | 205 | 205 | 205 | 205 | 205 | 3--3 |
|  |  |  | CCATGTACCCATCAAAACTTCA | 56 |  |  |  |  |  |  |  |
| Pr032286166 | BP-386 | (CCT)_10_ | GGGGATCGTTTTCCTCTCTG | 60 | 271 | 0 | 0 | 0 | 0 | 0 |  |
|  |  |  | GCTGCTCAAAGCCAAGAGTT | 58 |  |  |  |  |  |  |  |
| Pr032286167 | BP-387 | (ACG)_6_ | GGTCGCCTATAGGTACGTAGAAAA | 60 | 219 | 220 | 220 | 220 | 220 | 220 | 2--2 |
|  |  |  | CCAGAAACTACCACGTGAACC | 60 |  |  |  |  |  |  |  |
| Pr032286168 | BP-388 | (AG)_11_ | GAATAGAGCGTTGCGCAGAT | 58 | 190 | 190 | 190 | 190 | 190 | 190 | 5--5 |
|  |  |  | CAGGTTCCTCTCCTCCACTG | 62 |  |  |  |  |  |  |  |
| Pr032286169 | BP-389 | (AG)_18_ | TCGGATTGGTGGGTCTATTT | 56 | 190 | 190 | 190 | 190 | 190 | 190 | 4--7 |
|  |  |  | CGAAACCCCTTTGATGAGTT | 56 |  |  |  |  |  |  |  |
| Pr032286170 | BP-390 | (CT)_10_ | CTTACCAGATTTGCCGCACT | 58 | 248 | 250 | 250 | 250 | 250 | 250 | 4--4 |
|  |  |  | AAGAGGGAGCACCACATGAC | 60 |  |  |  |  |  |  |  |
| Pr032286171 | BP-391 | (CT)_10_ | GGGGACTCTCAAACCTCCTT | 60 | 170 | 0 | 0 | 0 | 0 | 0 |  |
|  |  |  | TTCCAACGACCATTCGATTT | 54 |  |  |  |  |  |  |  |
| Pr032286172 | BP-392 | (AG)_17_ | ATCTCCTCTGCTTCTTCACA | 56 | 248 | 250 | 250 | 250 | 250 | 250 | 1--1 |
|  |  |  | ATCTCACACCTCCACTCCTC | 60 |  |  |  |  |  |  |  |
| Pr032286173 | BP-393 | (CT)_15_ | CCTGCAGTCGACCTAGAGGA | 62 | 161 | 0 | 0 | 0 | 0 | 0 |  |
|  |  |  | CCAAAAGTTTGACAGCATCAGA | 56 |  |  |  |  |  |  |  |
| Pr032286174 | BP-394 | (AT)_16_ | ATGGGAATGGACATGGACAC | 58 | 215 | 215 | 215 | 215 | 215 | 215 | 3--3 |
|  |  |  | GCCACATAAGACGACTTAGACACA | 60 |  |  |  |  |  |  |  |
| Pr032286175 | BP-395 | (AAAT)_5_ | CCCTTCCTCCCTTTGCTTACAT | 60 | 147 | 145 | 145 | 145 | 145 | 145 | 3--3 |
|  |  |  | AGATGTGTGGCCCAAAGTTAGG | 60 |  |  |  |  |  |  |  |
| Pr032286176 | BP-396 | (AAAT)_5_ | CTGATTGACATCAGCCCAACAG | 60 | 169 | 0 | 0 | 0 | 0 | 0 |  |
|  |  |  | TGATAAAGGTCTGCCCAGGCTG | 62 |  |  |  |  |  |  |  |
| Pr032286177 | BP-397 | (AAAT)_5_ | CCCTCTGCAATGGACATCCTAG | 62 | 242 | 245 | 245 | 245 | 245 | 245 | 2--2 |
|  |  |  | AGAGCACTCCTAGCAGCTTCCC | 64 |  |  |  |  |  |  |  |
| Pr032286178 | BP-398 | (AAAT)_5_ | CCAATCTGGAGGGGAAAGTGTG | 62 | 161 | 0 | 0 | 0 | 0 | 0 |  |
|  |  |  | TCCTCCTCCCTCCCCTTTTTTG | 62 |  |  |  |  |  |  |  |
| Pr032286179 | BP-399 | (AAAT)_5_ | CGATCCGCTCCTTCATCGTTAC | 62 | 148 | 150 | 150 | 150 | 150 | 150 | 4--4 |
|  |  |  | GAGACTTGCGGCTTGTTCGTGG | 64 |  |  |  |  |  |  |  |
| Pr032286180 | BP-400 | (AAAT)_5_ | CCCCTTATGAGTCAAATGCGCA | 60 | 190 | 190 | 190 | 190 | 190 | 190 | 6--6 |
|  |  |  | TGCCTCGGACCCACCACCGCTG | 70 |  |  |  |  |  |  |  |
| Pr032286181 | BP-401 | (AAAT)_5_ | CCCAACACTCAAACATGGCTGA | 60 | 191 | 190 | 190 | 190 | 190 | 190 | 2--2 |
|  |  |  | TGGTGGACATGGGCCAATGATG | 62 |  |  |  |  |  |  |  |
| Pr032286182 | BP-402 | (AAAT)_5_ | CTCACCTCCTTCACATAACCTG | 60 | 235 | 235 | 235 | 235 | 235 | 235 | 3--3 |
|  |  |  | TTTCCTGGATTGGTCATGAGGG | 60 |  |  |  |  |  |  |  |
| Pr032286183 | BP-403 | (AAAT)_5_ | CGAGTCAAACCAGGAACTGATG | 60 | 172 | 0 | 0 | 0 | 0 | 0 |  |
|  |  |  | AACCTTGCCTGCACACAAAACG | 60 |  |  |  |  |  |  |  |
| Pr032286184 | BP-404 | (AAAT)_5_ | GGGAAAAGGGGTCTGCTGTCAA | 62 | 177 | 0 | 0 | 0 | 0 | 0 |  |
|  |  |  | TCTAACCGGCCTCCTTTGACTG | 62 |  |  |  |  |  |  |  |
| Pr032286185 | BP-405 | (AAAT)_5_ | GACAAGCAGCTAGCCACGTTTT | 60 | 242 | 240 | 240 | 240 | 240 | 240 | 5--5 |
|  |  |  | AGGAAGAAGGGGGATGACTTAG | 60 |  |  |  |  |  |  |  |
| Pr032286186 | BP-406 | (AAAT)_5_ | CTAGGGACAAGATTGACTGGGCTT | 62 | 215 | 215 | 215 | 215 | 215 | 215 | 3--3 |
|  |  |  | GCCTTAGTTACAACCCCCCAAAAG | 62 |  |  |  |  |  |  |  |
| Pr032286187 | BP-407 | (AAAT)_5_ | CCTTGGTAGCAAGCATTGATGGG | 62 | 166 | 0 | 0 | 0 | 0 | 0 |  |
|  |  |  | GCGGGAATGATTTCTGTGGCAGG | 64 |  |  |  |  |  |  |  |
| Pr032286188 | BP-408 | (AAAT)_5_ | CCGGTACAAAGCAACCTCTATG | 60 | 233 | 235 | 235 | 235 | 235 | 235 | 4--4 |
|  |  |  | AAGTCTTAGGCTCTTCGCCGAG | 62 |  |  |  |  |  |  |  |
| Pr032286189 | BP-409 | (AAAT)_5_ | CCGTTGGACTGCAAGAGGTTTT | 60 | 239 | 240 | 240 | 240 | 240 | 240 | 3--3 |
|  |  |  | GGTGCATGCGGAACTTTCACCG | 64 |  |  |  |  |  |  |  |
| Pr032286190 | BP-410 | (AAAT)_5_ | CGGAGACAATTCGGCCATCTACAG | 64 | 251 | 250 | 250 | 250 | 250 | 250 | 4--4 |
|  |  |  | CGAGTTGAAAGGAGTGGCATTCTG | 62 |  |  |  |  |  |  |  |
| Pr032286191 | BP-411 | (AAAT)_5_ | CAGCCTGTACATTCAGCTGCTGT | 62 | 151 | 150 | 150 | 150 | 150 | 150 | 6--6 |
|  |  |  | TCGGCCTGAGTCATTCATCCCAG | 64 |  |  |  |  |  |  |  |
| Pr032286192 | BP-412 | (AAAT)_5_ | CTTGGAGAGTCCTCCTGCACATTG | 64 | 180 | 180 | 180 | 180 | 180 | 180 | 3--3 |
|  |  |  | CGAATGGACTGTCGAACAGACTGG | 64 |  |  |  |  |  |  |  |
| Pr032286193 | BP-413 | (AAAT)_5_ | CCTATCGAGCTATAACCTCGGTC | 62 | 162 | 0 | 0 | 0 | 0 | 0 |  |
|  |  |  | CAACCGAAGATGGCTCATTAGGG | 62 |  |  |  |  |  |  |  |
| Pr032286194 | BP-414 | (AAAT)_5_ | CACGTCTAGGCATAACCCAGTAC | 62 | 213 | 215 | 215 | 215 | 215 | 215 | 2--2 |
|  |  |  | TTTTGCATGGTCGGGAGCTCTAG | 62 |  |  |  |  |  |  |  |
| Pr032286195 | BP-415 | (AAAT)_5_ | CTCATCTTACACCCTTAGCTCCC | 62 | 261 | 260 | 260 | 260 | 260 | 260 | 3--3 |
|  |  |  | ACAAGGATGCTTCCAGACCTCTC | 62 |  |  |  |  |  |  |  |
| Pr032286196 | BP-416 | (AAAT)_5_ | CGGGCATGCGTTGAGAATGAGA | 62 | 225 | 225 | 225 | 225 | 225 | 225 | 4--4 |
|  |  |  | GGTAGTTTGGAGAGCCGACATC | 62 |  |  |  |  |  |  |  |
| Pr032286197 | BP-417 | (AAAT)_5_ | CAAGTACTCAACCGCAGGTTCA | 60 | 200 | 200 | 200 | 200 | 200 | 200 | 3--3 |
|  |  |  | TCATGCAAGCTTGGAGTTCTGG | 60 |  |  |  |  |  |  |  |
| Pr032286198 | BP-418 | (AAAT)_5_ | CTCGAGCATTTTGCTTGGCGTT | 60 | 159 | 0 | 0 | 0 | 0 | 0 |  |
|  |  |  | ATCAGAGCTATAGGCTAACGCC | 60 |  |  |  |  |  |  |  |
| Pr032286199 | BP-419 | (AAAT)_5_ | CACTTTCACACCGGCCATATAGTG | 62 | 189 | 190 | 190 | 190 | 190 | 190 | 3--3 |
|  |  |  | TAAGAGTTGGTCAAATGGGGGTGG | 62 |  |  |  |  |  |  |  |
| Pr032286200 | BP-420 | (AAAT)_5_ | CCTCTACGCTCGGTATCCAGCC | 66 | 233 | 235 | 235 | 235 | 235 | 235 | 4--4 |
|  |  |  | CTGGATACCGAGCGTAGAGGCC | 66 |  |  |  |  |  |  |  |
| Pr032286201 | BP-421 | (AAAT)_5_ | GTAGTGGTGGAGCTAGGATCTCAT | 62 | 224 | 225 | 225 | 225 | 225 | 225 | 3--3 |
|  |  |  | GCTATTCAGCATTCTTAAGGGCCC | 62 |  |  |  |  |  |  |  |
| Pr032286202 | BP-422 | (AAAT)_5_ | GGAGAGAAATGAGGGAGCTCAA | 60 | 286 | 290 | 290 | 290 | 290 | 290 | 5--5 |
|  |  |  | CAGGTACATTTGGTGTGTGGTG | 60 |  |  |  |  |  |  |  |
| Pr032286203 | BP-423 | (AAAT)_5_ | CGAGCCCTCATTGCTCCAAAAGCC | 65 | 255 | 255 | 255 | 255 | 255 | 255 | 4--4 |
|  |  |  | TCGGGGTGGGTGCATATAAGGTGC | 65 |  |  |  |  |  |  |  |
| Pr032286204 | BP-424 | (AAAT)_5_ | CCTGGGGTGTTTATGGAGATAG | 60 | 160 | 160 | 160 | 160 | 160 | 160 | 2--2 |
|  |  |  | TACAAGTATCTGTTGCCCCCAC | 60 |  |  |  |  |  |  |  |
| Pr032286205 | BP-425 | (AAAT)_5_ | CCCTGGGCGTCCATGAGAGTTAGG | 67 | 196 | 200 | 200 | 200 | 200 | 200 | 2--2 |
|  |  |  | AATGAAGTGGGCGCTGAGGGATCG | 65 |  |  |  |  |  |  |  |
| Pr032286206 | BP-426 | (AAAT)_5_ | CAACAAGGTGCCCTAAACAAGG | 60 | 162 | 0 | 0 | 0 | 0 | 0 |  |
|  |  |  | CTAAGAGACTAATTAGGCGGCG | 60 |  |  |  |  |  |  |  |
| Pr032286207 | BP-427 | (AAAT)_5_ | CCCGATCCTGAAAAATAAGGTCGG | 62 | 245 | 250 | 250 | 250 | 250 | 250 | 6--6 |
|  |  |  | TGCAACTTGTGCACAGCTTAACGC | 62 |  |  |  |  |  |  |  |
| Pr032286208 | BP-428 | (AAAT)_5_ | GGGAGCACGTTTGGCACACTTTTT | 62 | 177 | 0 | 0 | 0 | 0 | 0 |  |
|  |  |  | CAAATGGCCATGTTCTGTACTGGG | 62 |  |  |  |  |  |  |  |
| Pr032286209 | BP-429 | (AAAT)_5_ | CGGTCTCACATTTGGGAAGAGG | 62 | 197 | 200 | 200 | 200 | 200 | 200 | 3--3 |
|  |  |  | GTGCTGGTCTTTTCCCCACTAG | 62 |  |  |  |  |  |  |  |
| Pr032286210 | BP-430 | (AAAT)_5_ | CCCACACATACACACTCTCTATGC | 62 | 213 | 215 | 215 | 215 | 215 | 215 | 3--3 |
|  |  |  | TGGTATGAGTGTGGGTCGTTTGAG | 62 |  |  |  |  |  |  |  |
| Pr032286211 | BP-431 | (AAAT)_5_ | CCTTAGTCATGGGTCAGCTGACCC | 65 | 276 | 275 | 275 | 275 | 275 | 275 | 2--2 |
|  |  |  | AGGCCTTGTGTTTGCCGACCCATG | 65 |  |  |  |  |  |  |  |
| Pr032286212 | BP-432 | (AAAT)_5_ | GGATAGCCACCTTAGCACAACA | 60 | 275 | 275 | 275 | 275 | 275 | 275 | 5--5 |
|  |  |  | GGTCAAAACCTATAAGCGGCAG | 60 |  |  |  |  |  |  |  |
| Pr032286213 | BP-433 | (AAAT)_5_ | CCATGTCTCCCCAGCCCTACTCT | 66 | 216 | 215 | 215 | 215 | 215 | 215 | 3--3 |
|  |  |  | AGGGTCTACCTGCATCCCTCCAG | 66 |  |  |  |  |  |  |  |
| Pr032286214 | BP-434 | (AAAT)_5_ | CCGTGAACGGTACAAGCCCAAGC | 66 | 186 | 0 | 0 | 0 | 0 | 0 |  |
|  |  |  | CATGGCCTCATGGGTCTCACAGG | 66 |  |  |  |  |  |  |  |
| Pr032286215 | BP-435 | (AAAT)_5_ | CCGATCCACACCCCAATTTCTT | 60 | 195 | 195 | 195 | 195 | 195 | 195 | 3--3 |
|  |  |  | GAGCCCCTCGAAATGACTAGTA | 60 |  |  |  |  |  |  |  |
| Pr032286216 | BP-436 | (AAAT)_5_ | CCAGGGATTTCCCCAAAGAGAT | 60 | 271 | 270 | 270 | 270 | 270 | 270 | 2--2 |
|  |  |  | TGAAGTGGCCTTATCTTGGAGG | 60 |  |  |  |  |  |  |  |
| Pr032286217 | BP-437 | (AAAT)_5_ | CTCTGATGAACACAACCTGCCTTG | 62 | 171 | 0 | 0 | 0 | 0 | 0 |  |
|  |  |  | AAATTGATAAGCTGCGTGGGTCGC | 62 |  |  |  |  |  |  |  |
| Pr032286218 | BP-438 | (AAAT)_5_ | CTGTGGTGACGGGTTTGCCTTTT | 62 | 187 | 190 | 190 | 190 | 190 | 190 | 3--3 |
|  |  |  | CCGAAGAAATAGGACCTGGGTTG | 62 |  |  |  |  |  |  |  |
| Pr032286219 | BP-439 | (AAAT)_5_ | CAGAGACTCAAAACTCTGGGGC | 62 | 272 | 275 | 275 | 275 | 275 | 275 | 5--5 |
|  |  |  | CTGCAACACCACCACCACCAAG | 64 |  |  |  |  |  |  |  |
| Pr032286220 | BP-440 | (AAAT)_5_ | CAACTCTTCCATTGCAGACCCGTG | 64 | 216 | 0 | 0 | 0 | 0 | 0 |  |
|  |  |  | AATGCTTGAGGAGGCAGGCCTTTG | 64 |  |  |  |  |  |  |  |
| Pr032286221 | BP-441 | (AAAT)_5_ | CGCAAGCTGTGTGACTAGCCCAAG | 65 | 254 | 250 | 250 | 250 | 250 | 250 | 2--2 |
|  |  |  | GGGGTGCATAGGCGTGAGACTTAC | 65 |  |  |  |  |  |  |  |
| Pr032286222 | BP-442 | (AAAT)_5_ | CAGCTCACCGGTTGACTAAGCT | 62 | 214 | 215 | 215 | 215 | 215 | 215 | 3--3 |
|  |  |  | AGGTCGACCTTGAGAAGCGTAG | 62 |  |  |  |  |  |  |  |
| Pr032286223 | BP-443 | (AAAT)_5_ | CATCTAGGCCAAGAAATAGGCT | 58 | 270 | 270 | 270 | 270 | 270 | 270 | 3--3 |
|  |  |  | CAAATCGCGTGCTTACTCAAGG | 60 |  |  |  |  |  |  |  |
| Pr032286224 | BP-444 | (AAAT)_5_ | GTAGGTAAGCATGTGCGGATAGGT | 62 | 139 | 0 | 0 | 0 | 0 | 0 |  |
|  |  |  | CCTTTTACCATACATACCCGGTGG | 62 |  |  |  |  |  |  |  |
| Pr032286225 | BP-445 | (AAAT)_5_ | CTCGTGGTTGCATCTCTTTCCA | 60 | 219 | 220 | 220 | 220 | 220 | 220 | 4--4 |
|  |  |  | TTGACCAAACCTGCCGAATGTG | 60 |  |  |  |  |  |  |  |
| Pr032286226 | BP-446 | (AAAT)_5_ | CCTTTACGTTCTTGTTCGGGCA | 60 | 264 | 0 | 0 | 0 | 0 | 0 |  |
|  |  |  | TCCATCTTCTTCTCGGCAATCC | 60 |  |  |  |  |  |  |  |
| Pr032286227 | BP-447 | (AAAT)_5_ | CAGGGTGGTGTGGTCCGTGATG | 66 | 231 | 0 | 0 | 0 | 0 | 0 |  |
|  |  |  | AGCTCCACCCGAGCAAGCAAGG | 66 |  |  |  |  |  |  |  |
| Pr032286228 | BP-448 | (AAAT)_5_ | CTTCTCCCCCAAGCCTTTCTTCTC | 64 | 241 | 240 | 240 | 240 | 240 | 240 | 5--5 |
|  |  |  | AGGATCCGGCTTTCTTCCTTCCTG | 64 |  |  |  |  |  |  |  |
| Pr032286229 | BP-449 | (AAAT)_5_ | CACAGACTTCTCCACTATCAGC | 60 | 249 | 250 | 250 | 250 | 250 | 250 | 3--3 |
|  |  |  | TTGCTGCACAAAATGAGCGTCC | 60 |  |  |  |  |  |  |  |
| Pr032286230 | BP-450 | (AAAT)_5_ | CGAAGCCTATCACAACCACAAG | 60 | 184 | 0 | 0 | 0 | 0 | 0 |  |
|  |  |  | TGGACGGTAAGTTGATCAAGGG | 60 |  |  |  |  |  |  |  |
| Pr032286231 | BP-451 | (AAAT)_5_ | GCGTGATACCCTCTCTACCTAA | 60 | 191 | 0 | 0 | 0 | 0 | 0 |  |
|  |  |  | TGTTGTCATCACCTGCATCTGC | 60 |  |  |  |  |  |  |  |
| Pr032286232 | BP-452 | (AAAT)_5_ | GGGGTAATGCTCCAGTGCTATA | 60 | 210 | 210 | 210 | 210 | 210 | 210 | 3--3 |
|  |  |  | TTTCCACACTTCATCTCCCCAG | 60 |  |  |  |  |  |  |  |
| Pr032286233 | BP-453 | (AAT)_8_ | CGGTGAGGTGGGGAACCCATTG | 66 | 223 | 225 | 225 | 225 | 225 | 225 | 2--2 |
|  |  |  | TGGAAACTGGAACCCCCACCCG | 66 |  |  |  |  |  |  |  |
| Pr032286234 | BP-454 | (AAT)_8_ | CGAAGCTACTTGCTGGCCATTA | 62 | 280 | 280 | 280 | 280 | 280 | 280 | 3--3 |
|  |  |  | AAAGATGGTTCGGCCACCTTCG | 62 |  |  |  |  |  |  |  |
| Pr032286235 | BP-455 | (AAT)_8_ | CCTTATCGGGCCTCGTTCATTA | 60 | 187 | 0 | 0 | 0 | 0 | 0 |  |
|  |  |  | GATACGGAAGTGACGTGTCAAC | 60 |  |  |  |  |  |  |  |
| Pr032286236 | BP-456 | (AAT)_8_ | CGAGTAAGGGTGACATATGGTC | 60 | 182 | 0 | 0 | 0 | 0 | 0 |  |
|  |  |  | CATCTACTCTTCTCCGCTTCTC | 60 |  |  |  |  |  |  |  |
| Pr032286237 | BP-457 | (AAT)_8_ | CCAACTTCTGTTTGGGACATGG | 60 | 176 | 0 | 0 | 0 | 0 | 0 |  |
|  |  |  | ATGGTTCTAATCCTTGGGGCAG | 60 |  |  |  |  |  |  |  |
| Pr032286238 | BP-458 | (AAT)_8_ | CACTGTACATCGTCCCTAGTCA | 60 | 202 | 200 | 200 | 200 | 200 | 200 | 1--1 |
|  |  |  | CCGTTAGATGCCACATCAACAC | 60 |  |  |  |  |  |  |  |
| Pr032286239 | BP-459 | (AAT)_8_ | CCCTCTTCCCTTCCCCAGTCTT | 64 | 171 | 0 | 0 | 0 | 0 | 0 |  |
|  |  |  | GAAGAGGGCGGTGCACAGCATC | 66 |  |  |  |  |  |  |  |
| Pr032286240 | BP-460 | (AAT)_8_ | GGGTGGTTCCGCTACTCTAAAA | 60 | 152 | 150 | 150 | 150 | 150 | 150 | 2--2 |
|  |  |  | CGCATGTGAATCTAACGGCTTG | 60 |  |  |  |  |  |  |  |
| Pr032286241 | BP-461 | (AAT)_8_ | GCCGGCCGGAATCTGCTGAAAT | 64 | 247 | 250 | 250 | 250 | 250 | 250 | 2--2 |
|  |  |  | GGATTGAGCCTCTTGCCCTCTC | 64 |  |  |  |  |  |  |  |
| Pr032286242 | BP-462 | (AAT)_8_ | GAAGAAGAAAGACCCCCTCCCT | 62 | 194 | 200 | 200 | 200 | 200 | 200 | 4--4 |
|  |  |  | ACAATGGTGGGGATGTCACGTG | 62 |  |  |  |  |  |  |  |
| Pr032286243 | BP-463 | (AAT)_8_ | CCATGATGATCCCTGCATATTGCC | 62 | 218 | 220 | 220 | 220 | 220 | 220 | 5--5 |
|  |  |  | GATTGCACGGTTTGGGTCAAGATC | 62 |  |  |  |  |  |  |  |
| Pr032286244 | BP-464 | (AAT)_8_ | GAACTGGTGCACCTTTTAGGGT | 60 | 212 | 0 | 0 | 0 | 0 | 0 |  |
|  |  |  | TTCCCCTATCAAAAGCCACCAC | 60 |  |  |  |  |  |  |  |
| Pr032286245 | BP-465 | (AAT)_8_ | CCAACAAAGGGGTGGCTTGTAA | 60 | 204 | 200 | 200 | 200 | 200 | 200 | 3--3 |
|  |  |  | AGACCCCGTTTTCAATTTGGCC | 60 |  |  |  |  |  |  |  |
| Pr032286246 | BP-466 | (AAT)_8_ | CTGCTCTCTGCACACACAAGTA | 60 | 199 | 200 | 200 | 200 | 200 | 200 | 4--4 |
|  |  |  | TGATTTGTGGGCCCATGAACAG | 60 |  |  |  |  |  |  |  |
| Pr032286247 | BP-467 | (AAT)_8_ | CGTAAGCCTTGGAAGCTTCGAA | 60 | 238 | 0 | 0 | 0 | 0 | 0 |  |
|  |  |  | GCTGCTGACATAGCACATAACG | 60 |  |  |  |  |  |  |  |
| Pr032286248 | BP-468 | (AAT)_8_ | CAAGCCAGAAAGGTTGAACACG | 60 | 248 | 250 | 250 | 250 | 250 | 250 | 3--3 |
|  |  |  | ATCAAATTGGGGCTCACCTCTC | 60 |  |  |  |  |  |  |  |
| Pr032286249 | BP-469 | (AAT)_8_ | GGAGGGAGTTAGTCGGTGAAAA | 60 | 207 | 210 | 210 | 210 | 210 | 210 | 6--6 |
|  |  |  | TTTACTTTTCTCCGGCCGACAG | 60 |  |  |  |  |  |  |  |
| Pr032286250 | BP-470 | (AAT)_8_ | GTTTCAGCACTGGTTCAAGCCA | 60 | 141 | 0 | 0 | 0 | 0 | 0 |  |
|  |  |  | GGGTGGGAATGTGAATATTGGG | 60 |  |  |  |  |  |  |  |
| Pr032286251 | BP-471 | (AAT)_8_ | CGTGAGAGATAGTCTCTCACAC | 60 | 206 | 205 | 205 | 205 | 205 | 205 | 5--5 |
|  |  |  | ATAAAGTCTCGCGTACCGAAGC | 60 |  |  |  |  |  |  |  |
| Pr032286252 | BP-472 | (AAT)_8_ | CGATGACACGTGTTGTACCCTA | 60 | 227 | 230 | 230 | 230 | 230 | 230 | 3--3 |
|  |  |  | GGTGGCAACATCACCTTTAAGG | 60 |  |  |  |  |  |  |  |
| Pr032286253 | BP-473 | (AAT)_8_ | CCATTCATCGCGGTCGACTGTTG | 64 | 247 | 250 | 250 | 250 | 250 | 250 | 7--7 |
|  |  |  | AGAGGCGGTCAGCAACAGTGATC | 64 |  |  |  |  |  |  |  |
| Pr032286254 | BP-474 | (AAT)_8_ | CTGATGCTGGCTTTAAGTGGGA | 60 | 144 | 0 | 0 | 0 | 0 | 0 |  |
|  |  |  | AACAATCTCTGCCTGATGTCGG | 60 |  |  |  |  |  |  |  |
| Pr032286255 | BP-475 | (AAT)_8_ | CGCGTGTTAGGTCGAAGCATAT | 60 | 165 | 165 | 165 | 165 | 165 | 165 | 3--3 |
|  |  |  | TTCGCAACGACTTCGTATGTCG | 60 |  |  |  |  |  |  |  |
| Pr032286256 | BP-476 | (AAT)_8_ | CATTTGCGTAGTACTGGGCCTT | 60 | 166 | 165 | 165 | 165 | 165 | 165 | 4--4 |
|  |  |  | TCGCGCCCTATTAAGTAGAGAG | 60 |  |  |  |  |  |  |  |
| Pr032286257 | BP-477 | (AAT)_8_ | CCTCTCTCGTTAGGGTTTGGGG | 64 | 277 | 0 | 0 | 0 | 0 | 0 |  |
|  |  |  | ATCGCTCTTGCAATCACCCCCC | 64 |  |  |  |  |  |  |  |
| Pr032286258 | BP-478 | (AAT)_8_ | GCTAGACCTTTGGAGTAAGGGT | 60 | 198 | 200 | 200 | 200 | 200 | 200 | 3--3 |
|  |  |  | GCCGAATGGGTATATTGCTTGC | 60 |  |  |  |  |  |  |  |
| Pr032286259 | BP-479 | (AAT)_8_ | CCCATCTCGCTTCCTTCCTCAA | 62 | 187 | 185 | 185 | 185 | 185 | 185 | 2--2 |
|  |  |  | CAAAAGTGGGCTTGCGTACGTG | 62 |  |  |  |  |  |  |  |
| Pr032286260 | BP-480 | (AAT)_8_ | CCGACGGTTGGTAAGCATGAGA | 62 | 222 | 0 | 0 | 0 | 0 | 0 |  |
|  |  |  | ATGGGATCGTGCCACATGTCAC | 62 |  |  |  |  |  |  |  |
| Pr032286261 | BP-481 | (AAT)_8_ | CCCCGCCGGTAAGAAATATCTC | 62 | 251 | 250 | 250 | 250 | 250 | 250 | 3--3 |
|  |  |  | ACTCCCATTGGCCAAATGGGTG | 62 |  |  |  |  |  |  |  |
| Pr032286262 | BP-482 | (AAT)_8_ | CCTCGTACTTGGGTATTGCCAA | 60 | 218 | 0 | 0 | 0 | 0 | 0 |  |
|  |  |  | TCACACTTTGCTGACGTGACAC | 60 |  |  |  |  |  |  |  |
| Pr032286263 | BP-483 | (AAT)_8_ | GGCCCAATTCACCTGTCAGCAA | 62 | 210 | 210 | 210 | 210 | 210 | 210 | 5--5 |
|  |  |  | TGACGGGATAGTGCCACATCAG | 62 |  |  |  |  |  |  |  |
| Pr032286264 | BP-484 | (AC)_10_ | GGGCTATGAGCCCAGACATTTA | 60 | 264 | 0 | 0 | 0 | 0 | 0 |  |
|  |  |  | TATGCATGAAGGAGGAGCAGAG | 60 |  |  |  |  |  |  |  |
| Pr032286265 | BP-485 | (AC)_10_ | CCAGCTGCAGCAGCAAGCTACT | 64 | 248 | 250 | 250 | 250 | 250 | 250 | 3--3 |
|  |  |  | GCAATGTGCCGGCTATGGTAGG | 64 |  |  |  |  |  |  |  |
| Pr032286266 | BP-486 | (AC)_10_ | CACCACCTTCTTACACCCCATC | 62 | 388 | 0 | 0 | 0 | 0 | 0 |  |
|  |  |  | CACTAATTCCTGCAGCAGGCTG | 62 |  |  |  |  |  |  |  |
| Pr032286267 | BP-487 | (AC)_10_ | GCTTTCCCCATAGGACTGTGTA | 60 | 297 | 300 | 300 | 300 | 300 | 300 | 3--3 |
|  |  |  | CCTAAGAATGTACAAGCAGGGC | 60 |  |  |  |  |  |  |  |
| Pr032286268 | BP-488 | (AC)_10_ | CGGCAGCCATCATGTAGCTCAA | 62 | 192 | 0 | 0 | 0 | 0 | 0 |  |
|  |  |  | GGTTGTCCAAAGACGGGTATGC | 62 |  |  |  |  |  |  |  |
| Pr032286269 | BP-489 | (AC)_10_ | CGCTCGCATACGGCTAGGGTTT | 64 | 227 | 225 | 225 | 225 | 225 | 225 | 4--4 |
|  |  |  | CTCTTCCCCCACTCTGCTTCTC | 64 |  |  |  |  |  |  |  |
| Pr032286270 | BP-490 | (AC)_10_ | CTCTCTCCCTCTTATCGCTCAC | 62 | 170 | 0 | 0 | 0 | 0 | 0 |  |
|  |  |  | TAAGGGCCTTTCAGTGCTTGCG | 62 |  |  |  |  |  |  |  |
| Pr032286271 | BP-491 | (TG)_10_ | CTCAACCAGATCAACTCGTCCA | 60 | 310 | 300 | 300 | 300 | 300 | 300 | 3--3 |
|  |  |  | GGACACAAGGTAGATATAGGGG | 60 |  |  |  |  |  |  |  |
| Pr032286272 | BP-492 | (TG)_10_ | CCAACATGCTGTGGGAAAGTTC | 60 | 149 | 0 | 0 | 0 | 0 | 0 |  |
|  |  |  | TATATAGACACACACCCGCGTG | 60 |  |  |  |  |  |  |  |
| Pr032286273 | BP-493 | (TG)_10_ | CATTGGGTGGGACATTAGCAACTG | 62 | 247 | 250 | 250 | 250 | 250 | 250 | 3--3 |
|  |  |  | GGGGTTTGCACAATGGGTTGATTC | 62 |  |  |  |  |  |  |  |
| Pr032286274 | BP-494 | (TG)_10_ | CATTGGGCTGATCGACTGGGAT | 62 | 179 | 0 | 0 | 0 | 0 | 0 |  |
|  |  |  | GAACCAACACCTGTACCTGTGG | 62 |  |  |  |  |  |  |  |
| Pr032286275 | BP-495 | (AGG)_7_ | CTAGCTTCCCAACTGAACTCAC | 60 | 147 | 145 | 145 | 145 | 145 | 145 | 2--2 |
|  |  |  | GGTGGAACTGTAAGTTTGTGGG | 60 |  |  |  |  |  |  |  |
| Pr032286276 | BP-496 | (AGG)_7_ | GTTGGGGTCCTATGCCAACTTT | 60 | 138 | 0 | 0 | 0 | 0 | 0 |  |
|  |  |  | GTTGCTTCCTGCTAAAAAGCCG | 60 |  |  |  |  |  |  |  |
| Pr032286277 | BP-497 | (AGG)_7_ | GCAGGGCAGAGGCATCTATTAA | 60 | 194 | 195 | 195 | 195 | 195 | 195 | 3--3 |
|  |  |  | CTCAACCTCAGAATTTCTGCCG | 60 |  |  |  |  |  |  |  |
| Pr032286278 | BP-498 | (AGG)_7_ | CCCCAAGACTCTTTGCCGGAGT | 64 | 215 | 215 | 215 | 215 | 215 | 215 | 2--2 |
|  |  |  | CTCGAAGCTGTCGTCGTCTTCG | 64 |  |  |  |  |  |  |  |
| Pr032286279 | BP-499 | (AGG)_7_ | GAAAGAGAGGTGGAGGAGGTGG | 64 | 173 | 0 | 0 | 0 | 0 | 0 |  |
|  |  |  | CCCCGACAAGAGACTCTCACTC | 64 |  |  |  |  |  |  |  |
| Pr032286280 | BP-500 | (AGG)_7_ | CAAGTGACCCATGCATCTGATG | 60 | 233 | 235 | 235 | 235 | 235 | 235 | 3--3 |
|  |  |  | ATAGTTGGGGCCCAGCATATTG | 60 |  |  |  |  |  |  |  |
| Pr032286281 | BP-501 | (AGG)_7_ | CCAGACCGCAACTCCTCCAGAG | 66 | 229 | 230 | 230 | 230 | 230 | 230 | 4--4 |
|  |  |  | TGGGTTTGGGAGGAGTGGTGCG | 66 |  |  |  |  |  |  |  |
| Pr032286282 | BP-502 | (GAA)_10_ | CCCCATGGATAGGATCAAGAGC | 62 | 172 | 0 | 0 | 0 | 0 | 0 |  |
|  |  |  | CACCTCTCTTGGGTTCCTCTTG | 62 |  |  |  |  |  |  |  |
| Pr032286283 | BP-503 | (TC)_13_ | CTCTCTCTTACGACATGGCAGT | 60 | 174 | 175 | 175 | 175 | 175 | 175 | 1--1 |
|  |  |  | GTGGAAGGCATTTTGGGAAGAG | 60 |  |  |  |  |  |  |  |
| Pr032286284 | BP-504 | (TC)_13_ | CGATGGTGGTTACTCGCATCTAGA | 62 | 234 | 0 | 0 | 0 | 0 | 0 |  |
|  |  |  | CCTTGAGATGTTCTCACCCACAAC | 62 |  |  |  |  |  |  |  |
| Pr032286285 | BP-505 | (TC)_13_ | CCACTGCATCTGATTCTCTCTC | 60 | 170 | 170 | 170 | 170 | 170 | 170 | 2--2 |
|  |  |  | GATGAGAGAGAGACAGAGAGAC | 60 |  |  |  |  |  |  |  |
| Pr032286286 | BP-506 | (TC)_13_ | CTGATTTCTTCCCCTCTCTCTC | 60 | 209 | 210 | 210 | 210 | 210 | 210 | 3--3 |
|  |  |  | TTTTTAGTGGGTTGCTGGGTGC | 60 |  |  |  |  |  |  |  |
| Pr032286287 | BP-507 | (TC)_13_ | GGGGAATGGTTGGAAACCGAGA | 62 | 262 | 260 | 260 | 260 | 260 | 260 | 5--5 |
|  |  |  | CGGGGATATTTTAGCCTGCCAG | 62 |  |  |  |  |  |  |  |
| Pr032286288 | BP-508 | (TC)_13_ | CTAGAAGGATGGCTAGTTGCCA | 60 | 259 | 260 | 260 | 260 | 260 | 260 | 5--5 |
|  |  |  | GTACGTACCTTTAGTGTCGAGG | 60 |  |  |  |  |  |  |  |
| Pr032286289 | BP-509 | (TC)_13_ | CGGGAACGCGTCTTGGGACAGC | 68 | 227 | 0 | 0 | 0 | 0 | 0 |  |
|  |  |  | CCATGGCGTCTCTCCCGCAGAG | 68 |  |  |  |  |  |  |  |
| Pr032286290 | BP-510 | (TC)_13_ | CACTCATGTCAGCTGATTCTCC | 60 | 189 | 190 | 190 | 190 | 190 | 190 | 3--5 |
|  |  |  | TCTGAGAGACTGAGAGAGAGAG | 60 |  |  |  |  |  |  |  |
| Pr032286291 | BP-511 | (TC)_13_ | CAAAGGTGAGGTATGTGTGGAC | 60 | 297 | 0 | 0 | 0 | 0 | 0 |  |
|  |  |  | AGGGGTAGGGATTACTACAGTG | 60 |  |  |  |  |  |  |  |
| Pr032286292 | BP-512 | (TC)_13_ | GGACCCAAACCTGATTCAAGCA | 60 | 240 | 240 | 240 | 240 | 240 | 240 | 3--3 |
|  |  |  | GCATATCCTGCCTCTTTCTAGC | 60 |  |  |  |  |  |  |  |
| Pr032286293 | BP-513 | (TC)_13_ | CATGCAATCAAGCGTGTTGAGG | 60 | 252 | 0 | 0 | 0 | 0 | 0 |  |
|  |  |  | TCTATGATAGCCAGTCTGGCAG | 60 |  |  |  |  |  |  |  |
| Pr032286294 | BP-514 | (TC)_13_ | CAGCTGGTCAGCTTCTTCAAGT | 60 | 278 | 280 | 280 | 280 | 280 | 280 | 4--4 |
|  |  |  | CACAATTTGCAGGCCATTGGAG | 60 |  |  |  |  |  |  |  |
| Pr032286295 | BP-515 | (TC)_13_ | CTTTCTCTCTCTCTCTCCATCG | 60 | 248 | 245 | 245 | 245 | 245 | 245 | 3--3 |
|  |  |  | TGTACATGGGTTGCCCTGTTTG | 60 |  |  |  |  |  |  |  |
| Pr032286296 | BP-516 | (TC)_13_ | CCATGTCCATGAGAATTGGCTC | 60 | 197 | 0 | 0 | 0 | 0 | 0 |  |
|  |  |  | TCAACAAAGGAAACCCCGAGAG | 60 |  |  |  |  |  |  |  |
| Pr032286297 | BP-517 | (TC)_13_ | GCAATCTCTCTCTCTCTCACCT | 60 | 193 | 195 | 195 | 195 | 195 | 195 | 2--2 |
|  |  |  | AACGTGTGTGTGAGAGAGAGAG | 60 |  |  |  |  |  |  |  |
| Pr032286298 | BP-518 | (TC)_13_ | CCAAATTTTCCCCTCCACCACT | 60 | 151 | 150 | 150 | 150 | 150 | 150 | 4--4 |
|  |  |  | TTCTATGGCGTCACCAATCTGG | 60 |  |  |  |  |  |  |  |
| Pr032286299 | BP-519 | (TC)_13_ | CAGCAAGTTTCCTCATGACTCC | 60 | 250 | 250 | 250 | 250 | 250 | 250 | 3--3 |
|  |  |  | CCTCGTGCAATAGCTAAGGAAG | 60 |  |  |  |  |  |  |  |
| Pr032286300 | BP-520 | (TC)_13_ | CCCTTCTGCATATATCCCAATCCC | 62 | 196 | 0 | 0 | 0 | 0 | 0 |  |
|  |  |  | ATGCACGCTCAAGCCTTGACATTG | 62 |  |  |  |  |  |  |  |
| Pr032286301 | BP-521 | (TC)_13_ | CACTTGCGAGCATGGATCTGTA | 60 | 183 | 0 | 0 | 0 | 0 | 0 |  |
|  |  |  | ACCCCACACACAAAACCTATGG | 60 |  |  |  |  |  |  |  |
| Pr032286302 | BP-522 | (TC)_13_ | CTCTGGCAATTTGGCCATTGCA | 60 | 208 | 205 | 205 | 205 | 205 | 205 | 2--2 |
|  |  |  | CAACACCTGACTTTCGATGCTG | 60 |  |  |  |  |  |  |  |
| Pr032286303 | BP-523 | (TC)_13_ | GGGCTTCCTTCCTGTATTTCAG | 60 | 180 | 180 | 180 | 180 | 180 | 180 | 4--4 |
|  |  |  | CCCTTGTCTGAAGCAACAGTAC | 60 |  |  |  |  |  |  |  |
| Pr032286304 | BP-524 | (TC)_13_ | CAGCTCTCCCCTCTCCTTTCTT | 62 | 191 | 190 | 190 | 190 | 190 | 190 | 6--6 |
|  |  |  | AGGAGGCTTCACGTGAAACCTG | 62 |  |  |  |  |  |  |  |
| Pr032286305 | BP-525 | (TC)_13_ | CGTCTTCACCAAGTAAGAACCC | 60 | 187 | 0 | 0 | 0 | 0 | 0 |  |
|  |  |  | AGGGTTCCATGTTAAGCCCTTC | 60 |  |  |  |  |  |  |  |
| Pr032286306 | BP-526 | (TC)_13_ | CCAATAGGCTGTGGAAGGCACA | 62 | 247 | 245 | 245 | 245 | 245 | 245 | 5--5 |
|  |  |  | CATCTGCGTCAAATCTGGTGGG | 62 |  |  |  |  |  |  |  |
| Pr032286307 | BP-527 | (TC)_13_ | GGCCTCTTCATGAGGAAAGACT | 60 | 221 | 220 | 220 | 220 | 220 | 220 | 3--3 |
|  |  |  | GTGGTAGGTGAAAACAGACGAC | 60 |  |  |  |  |  |  |  |
| Pr032286308 | BP-528 | (TC)_13_ | CATCAATACAGTACACGCGACG | 60 | 207 | 205 | 205 | 205 | 205 | 205 | 2--2 |
|  |  |  | CTCTGATCTCCTCTGCCTATTG | 60 |  |  |  |  |  |  |  |
| Pr032286309 | BP-529 | (TC)_13_ | CGAACTAGCCAAGTCTCACTTAGC | 62 | 193 | 0 | 0 | 0 | 0 | 0 |  |
|  |  |  | GCGATTTTGAGGATCCTTCCCTTG | 62 |  |  |  |  |  |  |  |
| Pr032286310 | BP-530 | (TC)_13_ | CTCCCTCATCACCCGTGCAACT | 64 | 225 | 225 | 225 | 225 | 225 | 225 | 3--3 |
|  |  |  | CACACACACACGCATGCGTTGG | 64 |  |  |  |  |  |  |  |
| Pr032286311 | BP-531 | (TC)_13_ | CAACGCAGACTCACAGCTATTA | 58 | 197 | 0 | 0 | 0 | 0 | 0 |  |
|  |  |  | TTTACGGACACAAGAGGCACTG | 60 |  |  |  |  |  |  |  |
| Pr032286312 | BP-532 | (TC)_13_ | CCATCTGAACTACCTGGAGCAG | 62 | 155 | 155 | 155 | 155 | 155 | 155 | 6--6 |
|  |  |  | TGTCGATTCCCCCTTAGATGGC | 62 |  |  |  |  |  |  |  |
| Pr032286313 | BP-533 | (TC)_13_ | CAGATGGTAGCTACCTAGCTAG | 60 | 306 | 0 | 0 | 0 | 0 | 0 |  |
|  |  |  | ACCCCTTGTCCAAGACTAATGG | 60 |  |  |  |  |  |  |  |
| Pr032286314 | BP-534 | (TC)_13_ | CTCTCACTGTAGTGGCTTCAGA | 60 | 381 | 0 | 0 | 0 | 0 | 0 |  |
|  |  |  | GAGTTTGTTCGCAGTTGGTTCG | 60 |  |  |  |  |  |  |  |
| Pr032286315 | BP-535 | (TC)_13_ | CGCCGTAAAGTATATCCCACCC | 62 | 236 | 235 | 235 | 235 | 235 | 235 | 2--2 |
|  |  |  | GCTGGATCGATCACCAGTGATC | 62 |  |  |  |  |  |  |  |
| Pr032286316 | BP-536 | (TC)_13_ | CAGTCAGACGATCGCCTTCACA | 62 | 194 | 195 | 195 | 195 | 195 | 195 | 3--3 |
|  |  |  | AGGCTTTATTGGGTTGCCTGGG | 62 |  |  |  |  |  |  |  |
| Pr032286317 | BP-537 | (TC)_13_ | GCTTAACCTCACCTACTCTTAGGG | 62 | 194 | 195 | 195 | 195 | 195 | 195 | 3--3 |
|  |  |  | TTCAACAAACATGGCTGGAGCCTG | 62 |  |  |  |  |  |  |  |
| Pr032286318 | BP-538 | (TC)_13_ | CAGAAAGGCATTCCCTCCAACT | 60 | 222 | 0 | 0 | 0 | 0 | 0 |  |
|  |  |  | ATGAAGCACTGGAGTCCCTTTG | 60 |  |  |  |  |  |  |  |
| AF310856 | AF310856 | (GA)_10_ | ACGCTTTCTTGATGTCAGCC | 58 | 189 | 190 | 190 | 190 | 190 | 190 | 4--9 |
|  |  |  | TCACCAAGTTCCTGGTGGAT | 58 |  |  |  |  |  |  |  |
| AF310847 | AF310847 | (AG)_16_ | CAGTGTTTGGACGGTGAGAA | 58 | 209 | 210 | 210 | 210 | 210 | 210 | 3--6 |
|  |  |  | CGGGTGAAGTAGACGGAACT | 60 |  |  |  |  |  |  |  |
| AF310866 | AF310866 | (CT)_11_ | GGCCAACAGATATAAAACGACG | 58 | 301 | 315 | 300 | 315 | 315 | 315 | 1--6 |
|  |  |  | TTTTAAATGCCCACCTTCCC | 56 |  |  |  |  |  |  |  |
| AF310858 | AF310858 | (AG)_17_ | TTGAGATAGACGATAGAGGTAAAGCA | 59 | 278 | 0 | 0 | 0 | 0 | 0 |  |
|  |  |  | AGGCATTTCTCCAA TTTTCTT | 52 |  |  |  |  |  |  |  |
| AF310868 | AF310868 | (AT)_8_ | AGCGACCCAATGCAGTTATC | 58 | 255 | 255 | 255 | 255 | 255 | 255 | 5--5 |
|  |  |  | CCGGCCACTCTTAGGTTTTT | 58 |  |  |  |  |  |  |  |
| AF310869 | AF310869 | (AT)_8_ | TTGAGCATTTCAACCACATCTT | 54 | 272 | 0 | 0 | 0 | 0 | 0 |  |
|  |  |  | TGTCTAAAGCTTGGGTGGCT | 58 |  |  |  |  |  |  |  |
